# Silver-Catalyzed Radical Umpolung Cross-Coupling of Silyl Enol Ethers with Activated Methylene Compounds: Access to Diverse Tricarbonyl Derivatives

**DOI:** 10.1021/acs.joc.4c00310

**Published:** 2024-06-15

**Authors:** Tongwei Liang, Qingjia Yuan, Li Xu, Jian-Quan Liu, Markus D. Kärkäs, Xiang-Shan Wang

**Affiliations:** † School of Chemistry and Materials Science, Jiangsu Key Laboratory of Green Synthesis for Functional Materials, Jiangsu Normal University, Xuzhou, Jiangsu 221116, China; ‡ Department of Chemistry, KTH Royal Institute of Technology, SE-100 44 Stockholm, Sweden

## Abstract

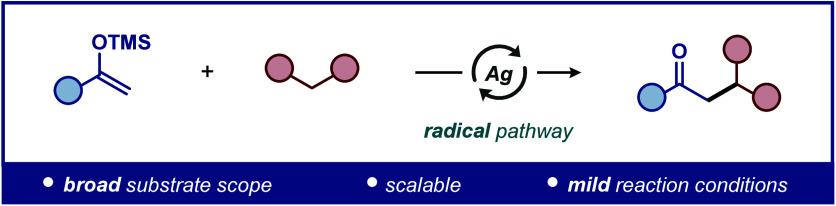

A silver-catalyzed protocol for the intermolecular radical umpolung cross-coupling protocol of silyl enol ethers with activated methylene compounds is disclosed. The protocol exhibits excellent functional group tolerance, enabling the expedient preparation of a variety of tricarbonyl compounds. Preliminary mechanistic investigations suggest that the reaction proceeds through a process involving free radicals in which silver oxide has a dual role, acting as both a catalyst and a base.

## Introduction

Catalytic radical carbon–carbon (C–C) cross-coupling reactions constitute privileged platforms in chemical synthesis, especially for the synthesis of natural products, pharmaceuticals, and materials.^1,2^ Although significant progress has been achieved over the past two decades, there is still strong interest in developing novel radical C–C coupling reactions using platform chemicals.

**Figure 1:**
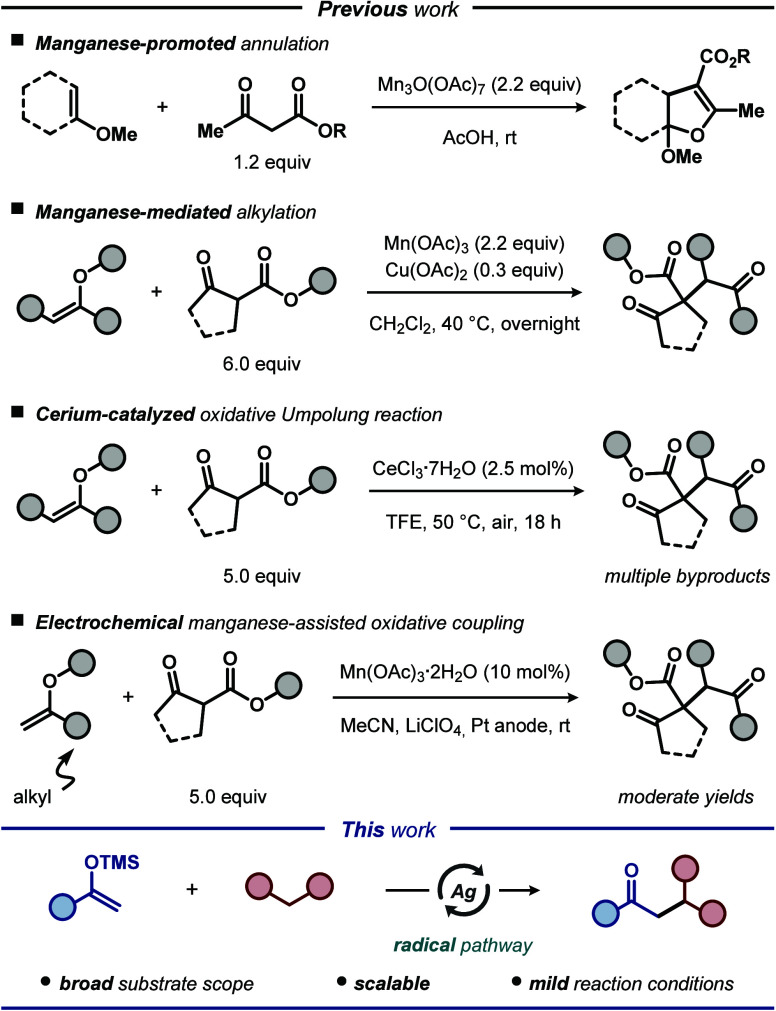
Cross-coupling reactions between enolates and β-dicarbonyl compounds.

Enolates and β-dicarbonyl compounds are versatile building blocks in organic synthesis, commonly used in cross-coupling reactions.^3,4^ Thus far, several notable transformations between these coupling partners have been disclosed, providing access to a wide range of tricarbonyl compounds (Figure 1). Unfortunately, several of the existing protocols suffer from significant drawbacks, including the need for stoichiometric quantities of chemical oxidants, limited functional group tolerance, and a propensity for competitive side reactions or low yields. In 1987, Corey and Chosh reported an innovative approach for accessing 1-alkoxy-1,2-dihydrofurans, involving the intermolecular cross-coupling of enol ethers with β-dicarbonyl compounds.^5^ Later, Parsons and co-workers employed this strategy for achieving alkylation of enol ethers or enol esters with 2-methyl-1,3-dicarbonyl compounds, providing tricarbonyl compounds bearing a quaternary carbon center.^6^ However, these approaches rely on the utilization of superstoichiometric amounts of chemical oxidants, such as manganese(III) acetate, and highly elaborate starting materials to form the intended products. In 2016, Christoffers and co-workers utilized a novel umpolung approach for the oxidative cross-coupling reaction of β-dicarbonyl compounds with enol acetates by employing catalytic amounts of cerium trichloride hydrate as a one-electron oxidant.^7^ In this strategy, although the issues of employing stoichiometric metal-based oxidants were circumvented, a large excess of one of the coupling partners is required, thus significantly reducing the atom economy of the developed protocol. In 2019, Hilt and co-workers demonstrated the electrochemical synthesis of C–C bonds between β-dicarbonyl compounds and an excess of the silyl enol ether coupling partner (5.0 equiv) in the presence of catalytic amounts of manganese salts.^8^ However, the developed methodology is limited to alkyl alkenyl silyl ethers and typically results in moderate yields. In 2015, we disclosed that silver demonstrates remarkable catalytic activity in triggering the free radical coupling between activated methylene compounds and isocyanides.^9^ Furthermore, we recently reported a silver-catalyzed protocol for the controlled intermolecular cross-coupling between various silyl enol ethers that proceeds through a process involving free radicals.^10,11^ Inspired by these earlier synthetic protocols, we herein address the limitations of the previously disclosed cross-coupling manifolds involving silyl enol ethers and activated methylene compounds by employing silver catalysis (Figure 1). The developed approach offers a convenient method for the selective synthesis of diverse tricarbonyl scaffolds under mild reaction conditions while utilizing nearly stoichiometric quantities of the two coupling partners.

## Results and Discussion

At the onset of our investigations, silyl enol ether **1a** and ethyl acetoacetate (**2a**) were used as model substrates for the optimization of the oxidative cross-coupling platform (see Table 1). To our delight, conducting the reaction in MeCN at ambient temperature and under an argon atmosphere in the presence of AgF (20 mol %) and bromobenzene (2 equiv) yielded the desired tricarbonyl compound **3a** in 33% isolated yield after 6 h (Table 1, entry 1). Encouraged by these results, we proceeded to conduct a comprehensive examination of additional silver-based precursors encompassing Ag_2_O, Ag_2_CO_3_, AgOTf, AgBF_4_, and AgOAc. This demonstrated that the reactivity of Ag_2_O was superior to those of the other silver salts (Table 1, entries 2–6, respectively). Other metal-based precursors, including CuI and Pd(OAc)_2_, did not afford any detectable amounts of product **3a** (Table 1, entries 7 and 8, respectively). Subsequently, the yield of cross-coupling product **3a** was significantly enhanced to 53% by substituting MeCN as the solvent with 1,4-dioxane (Table 1, entry 9). Conversely, the application of nonane or polar solvents, such as DMF, toluene, and DCE, had an adverse impact on the reaction (Table 1, entries 10–12, respectively). However, when EtOH was used as a protic solvent, the corresponding ketone was exclusively produced from the silyl enol ether (Table 1, entry 13). Control experiments validate that molecular oxygen is essential for the reaction, increasing the yield to 83% (Table 1, entry 14). Reducing the catalyst loading from 30 to 20 and 10 mol % significantly impacts the reaction outcomes; a catalyst loading of 20 mol % is adequate for the transformation (Table 1, entries 15 and 16, respectively). According to our prior experience and the control experiments, we concluded that bromobenzene does not have a substantial impact on the developed reaction (Table 1, entries 17 and 18). Additionally, a significant quantity of cross-coupling product **3a** can also be produced. The optimal reaction conditions are highlighted in entry 18 of Table 1.

**Table 1:**
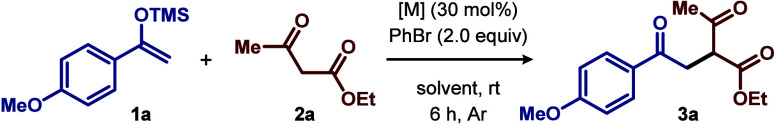
Optimization of the Reaction Conditions^a^,^b^

entry	[M]	solvent	yield (%)^b^
1	AgF	MeCN	33
2	Ag_2_O	MeCN	48
3	Ag_2_CO_3_	MeCN	46
4	AgOTf	MeCN	0
5	AgBF_4_	MeCN	0
6	AgOAc	MeCN	26
7	CuI	MeCN	0
8	Pd(OAc)_2_	MeCN	0
9	Ag_2_O	1,4-dioxane	53
10	Ag_2_O	DMF	42
11	Ag_2_O	toluene	47
12	Ag_2_O	DCE	31
13	Ag_2_O	EtOH	0
14^c^	Ag_2_O	1,4-dioxane	83
15^c^,^d^	Ag_2_O	1,4-dioxane	86
16^c^,^e^	Ag_2_O	1,4-dioxane	57
17^c^,^f^	Ag_2_O	1,4-dioxane	82
18^c^,^d^,^f^	Ag_2_O	1,4-dioxane	85

a Reactions were carried out with **1a** (1.0 mmol), **2a** (0.5 mmol), a catalyst (30 mol %), and PhBr (1.0 mmol) in a solvent (2.0 mL) at ambient temperature under argon for 6 h.

b Isolated yields of **3a** after purification by column chromatography.

c Reactions under air.

d With 20 mol % catalyst.

e With 10 mol % catalyst.

f Reaction in the absence of PhBr.

**Scheme 1:**
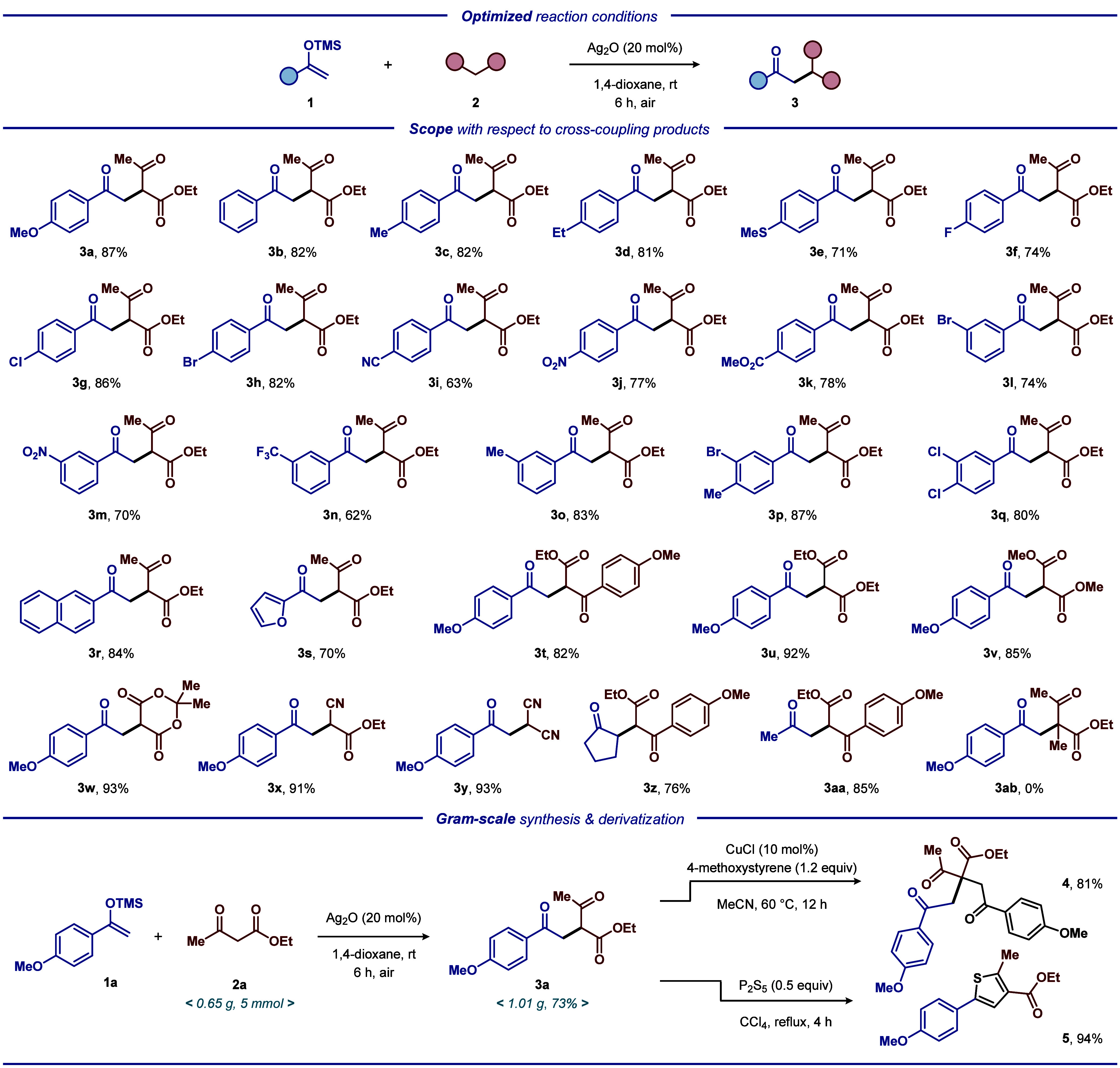
Silver-Catalyzed Synthesis of Tricarbonyl Derivatives, All reactions were carried out with **1a** (1.0 mmol), **2a** (0.5 mmol), and Ag_2_O (20 mol %) in 1,4-dioxane (2.0 mL) at room temperature under air for 6 h. Yields are of isolated products after purification by column chromatography.

Upon identifying the optimal reaction conditions for the developed transformation (Table 1, entry 18), we commenced our investigations by examining whether the protocol could be applied to various coupling partners (Scheme 1). A collection of differently functionalized silyl enol ethers **1** participated in the cross-coupling reaction with activated methylene compounds **2**, resulting in the formation of the corresponding products **3** in good to excellent yields (Scheme 1). For example, aryl-based silyl enol ether motifs containing electron-donating or electron-withdrawing moieties exhibited good tolerance, resulting in the formation of the corresponding products **3b**–**3q** in high yields. Gratifyingly, the successful utilization of heteroaryl silyl ethers and aromatic ring silyl ethers, such as 2-naphthyl and 2-furyl, resulted in the generation of the corresponding adducts **3r** and **3s**, demonstrating the compatibility of the established protocol. Additionally, other activated methylene compounds, such as dimethyl/diethyl malonate, 2,2-dimethyl-1,3-dioxane-4,6-dione, and malononitrile, were also suitable coupling partners, providing the corresponding products **3t**–**3y** in yields ranging from 82% to 93%. Gratifyingly, subjecting alkyl-based silyl enol ethers to ethyl (4-methoxybenzoyl)acetate afforded the desired products **3z** and **3aa** in 76% and 85% yields, respectively. Unfortunately, 2-methyl-1,3-dicarbonyl compounds, which would allow the formation of tricarbonyl compounds (**3ab**) featuring a quaternary carbon center, are not tolerated by the protocol. To further explore the synthetic utility of the developed protocol, the applicability of the silver-catalyzed approach was highlighted by conducting the reaction at a 5 mmol scale with **1a** and **2a**, thus leading to the successful synthesis of product **3a** (1.01 g, 73%) in a straightforward fashion (see Scheme 1). The conceived protocol successfully provides expedient access to highly decorated tricarbonyl derivatives, which can be used for further diverse synthetic manipulations. For example, tricarbonyl compound **3a** was employed as an entry to tetracarbonyl compound **4** and elaborate thiophene **5** upon reaction of **3a** with 4-methoxystyrene and diphosphorus pentasulfide, respectively (see Scheme 1).^12,13^

**Scheme 2:**
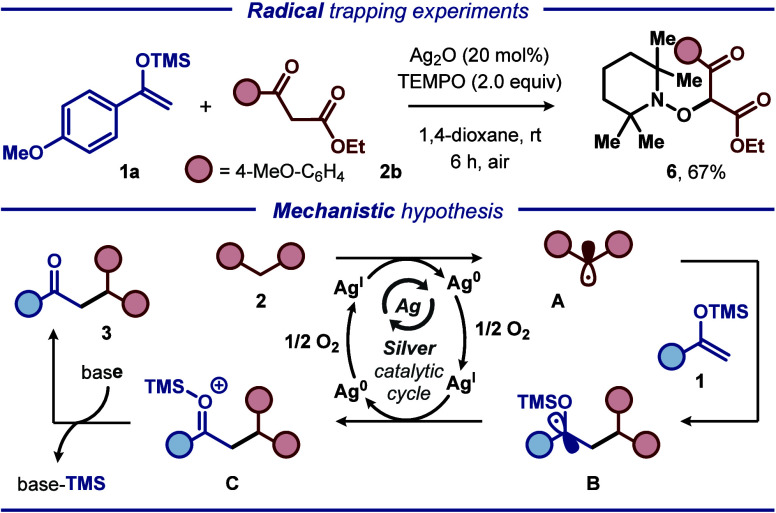
Radical Trapping Experiments and Mechanistic Hypothesis

To gain insight into the operating mechanism, radical inhibitor (2,2,6,6-tetramethylpiperidin-1-yl)oxyl (TEMPO) was added to the reaction mixture. TEMPO was shown to effectively suppress the silver-mediated cross-coupling reaction between silyl enol ethers **1a** and **2b**. Instead, adduct **6** could be isolated in 67% yield, indicating that the reaction proceeds through a free radical pathway.^14,15^ On the basis of the experimental results and literature precedent,^3,9,16,17,18^ a plausible mechanism for the cross-coupling reaction is proposed (Scheme 2). Initially, deprotonation and one-electron oxidation of activated methylene substrate **2** by Ag^I^ result in the formation of α-carbonyl radical **A**. The oxidizing behavior of silver can be explained by the frequently observed silver mirror during the reactions. Then, α-carbonyl radical **A** undergoes radical addition to silyl enol ether **1** to form carbon-centered radical **B**, which participates in a second single-electron transfer (SET) event to furnish cross-coupling product **3** along with regeneration of Ag^I^ upon reoxidation by O_2_,^19^ thereby closing the catalytic cycle. In the envisioned mechanism, Ag_2_O has a dual role and functions as both a base and a catalyst.

## Conclusions

In conclusion, we devised a silver-catalyzed intermolecular cross-coupling reaction between silyl enol ethers and activated methylene compounds. The protocol demonstrates excellent functional group tolerance, enabling expedient access to a variety of synthetically valuable tricarbonyl derivatives. Preliminary mechanistic investigations suggest that the reaction proceeds through a free radical-based pathway. The disclosed method offers a versatile framework for the oxidative formation of carbon–carbon bonds from simple starting materials.

## Experimental Section

### General Information

All reagents were purchased from commercial sources and used without treatment, unless otherwise indicated. The products were purified by column chromatography over silica gel. ^1^H nuclear magnetic resonance (NMR) and ^13^C NMR spectra were recorded at 25 °C on a Varian spectrometer at 400 and 101 MHz, respectively, with TMS as the internal standard. Mass spectra were recorded on a BRUKER AutoflexIII Smartbeam MS spectrometer. High-resolution mass spectra (HRMS) were recorded on a Bruker microTof instrument using ESI-TOF. Infrared spectroscopy was performed on a ThermoFisher Scientific Nicolet iS10 FTIR spectrometer.

### General Procedure for the Preparation of Silyl Enol Ethers^20^

NaI (1.4 mmol, 1.4 equiv) was placed in a tube and dried under vacuum using a heat gun. Upon being cooled to room temperature, the tube was filled with argon. Then, dry CH_3_CN (1.0 mL), ketone (1.0 mmol, 1.0 equiv), and Et_3_N (1.5 mmol, 1.5 equiv) were successively added. The mixture was cooled with an ice/water bath, and TMSCl (1.3 mmol, 1.3 equiv) was added at 0 °C. The cooling bath was removed, and the mixture was stirred at room temperature for 12 h. Then, the volatile components were evaporated under a vacuum. The solid residue was washed with petroleum ether (3 × 15 mL), and the petroleum ether layers were decanted and filtered through a cotton plug. The combined filtrates were concentrated on a rotary evaporator, furnishing the silyl enol ether, which was used without further purification.

### General Procedure for the Preparation of Products **3**

To a 10 mL Schlenk tube equipped with a magnetic stir bar were added silyl enol ether **1** (1 mmol, 2.0 equiv), compound **2** (0.5 mmol, 1.0 equiv), Ag_2_O (0.1 mmol, 0.2 equiv), and 1,4-dioxane (2.0 mL). The reaction mixture was stirred at room temperature in air for ∼6 h. The resulting mixture was concentrated, and the residue was taken up in ethyl acetate. The organic layer was washed with brine, dried over Na_2_SO_4_, and concentrated. Purification of the crude product by column chromatography (silica gel; petroleum ether/ethyl acetate) afforded product **3**.

## Data Availability

The data underlying this study are available in the published article and its Supporting Information.

## Supporting Information Available

Experimental procedures, characterization data, and copies of NMR spectra for all obtained products (PDF)
FAIR data, including the primary NMR FID files, for compounds **3a**–**3z**, **3aa**, and **4**–**6** (ZIP)
