# Supplementary material for: Silver-Catalyzed Radical Umpolung Cross-Coupling of Silyl Enol Ethers with Activated Methylene Compounds: Access to Diverse Tricarbonyl Derivatives
Source: J Org Chem. 2024 Jun 15;89(13):9298–302. doi: 10.1021/acs.joc.4c00310 (PMC11232002; doi:10.1021/acs.joc.4c00310)
Supplement: Supplementary file 1 — jo4c00310_si_001.pdf [file jo4c00310_si_001.pdf]

Supporting Information

Silver-Catalyzed Radical Umpolung Cross-Coupling of Silyl Enol Ethers with Activated Methylene Compounds: Access to Diverse Tricarbonyl Derivatives

Tongwei Liang,[†,‖] Qingjia Yuan,[†,‖] Li Xu,[†] Jian-Quan Liu,[†,*] ✉ Markus D. Kärkäs,[‡,*] ✉ Xiang-Shan Wang[†,*] ✉

[†] School of Chemistry and Materials Science, Jiangsu Key Laboratory of Green Synthesis for Functional Materials, Jiangsu Normal University, Xuzhou, Jiangsu 221116, China

[‡] Department of Chemistry, KTH Royal Institute of Technology, SE-100 44 Stockholm, Sweden

[‖] These authors contributed equally

✉ E-mail: liujq316@jsnu.edu.cn (J.-Q.L.); karkas@kth.se (M.D.K.); xswang@jsnu.edu.cn (X.-S.W)

Table of Contents

I. General information **S1**

II. Synthesis of compounds 1 .... **S1**

III. Synthesis and analytical data of compounds 3, 4 ,5 and 6 .... **S2**

Ethyl 2-acetyl-4-(4-methoxyphenyl)-4-oxobutanoate (**3a**) S2

Gram-scale synthesis of ethyl 2-acetyl-4-(4-methoxyphenyl)-4-oxobutanoate (**3a**) S2

Ethyl 2-acetyl-4-oxo-4-phenylbutanoate (**3b**) S3

Ethyl 2-acetyl-4-oxo-4-(*p*-tolyl)butanoate (**3c**) S3

Ethyl 2-acetyl-4-(4-ethylphenyl)-4-oxobutanoate (**3d**) S4

Ethyl 2-acetyl-4-(4-(methylthio)phenyl)-4-oxobutanoate (**3e**) S4

Ethyl 2-acetyl-4-(4-fluorophenyl)-4-oxobutanoate (**3f**) S5

Ethyl 2-acetyl-4-(4-chlorophenyl)-4-oxobutanoate (**3g**) S6

Ethyl 2-acetyl-4-(4-bromophenyl)-4-oxobutanoate (**3h**) S6

Ethyl 2-acetyl-4-(4-cyanophenyl)-4-oxobutanoate (**3i**) S7

Ethyl 2-acetyl-4-(4-nitrophenyl)-4-oxobutanoate (**3j**) S8

Methyl 4-(3-(ethoxycarbonyl)-4-oxopentanoyl)benzoate (**3k**) S8

Ethyl 2-acetyl-4-(3-bromophenyl)-4-oxobutanoate (**3l**) S9

Ethyl 2-acetyl-4-(3-nitrophenyl)-4-oxobutanoate (**3m**) S10

Ethyl 2-acetyl-4-oxo-4-(3-(trifluoromethyl)phenyl)butanoate (**3n**) S10

Ethyl 2-acetyl-4-oxo-4-(*m*-tolyl)butanoate (**3o**) S11

Ethyl 2-acetyl-4-(3-bromo-4-methylphenyl)-4-oxobutanoate (**3p**) S12

Ethyl 2-acetyl-4-(3,4-dichlorophenyl)-4-oxobutanoate (**3q**) S12

Ethyl 2-acetyl-4-(naphthalen-2-yl)-4-oxobutanoate (**3r**) S13

Ethyl 2-acetyl-4-(furan-2-yl)-4-oxobutanoate (**3s**) S14

Ethyl 2-(4-methoxybenzoyl)-4-(4-methoxyphenyl)-4-oxobutanoate (**3t**) S14

Diethyl 2-(2-(4-methoxyphenyl)-2-oxoethyl)malonate (**3u**) S15

Dimethyl 2-(2-(4-methoxyphenyl)-2-oxoethyl)malonate (**3v**) S16

5-(2-(4-Methoxyphenyl)-2-oxoethyl)-2,2-dimethyl-1,3-dioxane-4,6-dione (**3w**) S16

Ethyl 2-cyano-4-(4-methoxyphenyl)-4-oxobutanoate (**3x**) S17

2-(2-(4-Methoxyphenyl)-2-oxoethyl)malononitrile (**3y**) S18

Ethyl 3-(4-methoxyphenyl)-3-oxo-2-(2-oxocyclopentyl)propanoate (**3z**) S18

Ethyl 2-(4-methoxybenzoyl)-4-oxopentanoate (**3aa**) S19

Ethyl 2-acetyl-4-(4-methoxyphenyl)-2-(2-(4-methoxyphenyl)-2-oxoethyl)-4-oxobutanoate (**4**) S20

Ethyl 5-(4-methoxyphenyl)-2-methylthiophene-3-carboxylate (**5**) S20

Ethyl 3-(4-methoxyphenyl)-3-oxo-2-((2,2,6,6-tetramethylpiperidin-1-yl)oxy)propanoate (**6**) ... S21

IV. NMR spectra S22

Ethyl 2-acetyl-4-(4-methoxyphenyl)-4-oxobutanoate (**3a**) S22

Ethyl 2-acetyl-4-oxo-4-phenylbutanoate (**3b**) S23

Ethyl 2-acetyl-4-oxo-4-(*p*-tolyl)butanoate (**3c**) S24

Ethyl 2-acetyl-4-(4-ethylphenyl)-4-oxobutanoate (**3d**) S25

Ethyl 2-acetyl-4-(4-(methylthio)phenyl)-4-oxobutanoate (**3e**) S26

Ethyl 2-acetyl-4-(4-fluorophenyl)-4-oxobutanoate (**3f**) S27

Ethyl 2-acetyl-4-(4-chlorophenyl)-4-oxobutanoate (**3g**) S29

Ethyl 2-acetyl-4-(4-bromophenyl)-4-oxobutanoate (**3h**) S30

Ethyl 2-acetyl-4-(4-cyanophenyl)-4-oxobutanoate (**3i**) S31

Ethyl 2-acetyl-4-(4-nitrophenyl)-4-oxobutanoate (**3j**) S32

Methyl 4-(3-(ethoxycarbonyl)-4-oxopentanoyl)benzoate (**3k**) S33

Ethyl 2-acetyl-4-(3-bromophenyl)-4-oxobutanoate (**3l**) S34

Ethyl 2-acetyl-4-(3-nitrophenyl)-4-oxobutanoate (**3m**) S35

Ethyl 2-acetyl-4-oxo-4-(3-(trifluoromethyl)phenyl)butanoate (**3n**) S36

Ethyl 2-acetyl-4-oxo-4-(*m*-tolyl)butanoate (**3o**) S38

Ethyl 2-acetyl-4-(3-bromo-4-methylphenyl)-4-oxobutanoate (**3p**) S39

Ethyl 2-acetyl-4-(3,4-dichlorophenyl)-4-oxobutanoate (**3q**) S40

Ethyl 2-acetyl-4-(naphthalen-2-yl)-4-oxobutanoate (**3r**) S41

Ethyl 2-acetyl-4-(furan-2-yl)-4-oxobutanoate (**3s**) S42

3-(4-Methoxybenzoyl)-1-(4-methoxyphenyl)pentane-1,4-dione (**3t**) S43

Diethyl 2-(2-(4-methoxyphenyl)-2-oxoethyl)malonate (**3u**) S44

Dimethyl 2-(2-(4-methoxyphenyl)-2-oxoethyl)malonate (**3v**) S45

5-(2-(4-Methoxyphenyl)-2-oxoethyl)-2,2-dimethyl-1,3-dioxane-4,6-dione (**3w**) S46

Ethyl 2-cyano-4-(4-methoxyphenyl)-4-oxobutanoate (**3x**) S47

2-(2-(4-Methoxyphenyl)-2-oxoethyl)malononitrile (**3y**) S48

Ethyl 3-(4-methoxyphenyl)-3-oxo-2-(2-oxocyclopentyl)propanoate (**3z**) S49

Ethyl 2-(4-methoxybenzoyl)-4-oxopentanoate (**3aa**) S50

Ethyl 2-acetyl-4-(4-methoxyphenyl)-2-(2-(4-methoxyphenyl)-2-oxoethyl)-4-oxobutanoate (**4**) S51

Ethyl 5-(4-methoxyphenyl)-2-methylthiophene-3-carboxylate (**5**) S52

Ethyl 3-(4-methoxyphenyl)-3-oxo-2-((2,2,6,6-tetramethylpiperidin-1-yl)oxy)propanoate (**6**) ... S53

V. References S54

I. General information

All reagents were purchased from commercial sources and used without treatment unless otherwise indicated. The products were purified by column chromatography over silica gel. ^{1}H NMR and ^{13}C NMR spectra were recorded at 25 °C on a Varian spectrometer at 400 MHz and 101 MHz, respectively, with TMS as the internal standard. Mass spectra were recorded on a BRUKER AutoflexIII Smartbeam MS-spectrometer. High resolution mass spectra (HRMS) were recorded on Bruck microTof using ESI-TOF. Infrared spectroscopy was recorded on a Thermo Fisher Scientific Nicolet iS 10 FTIR spectrometer.

II. Synthesis of compounds 1

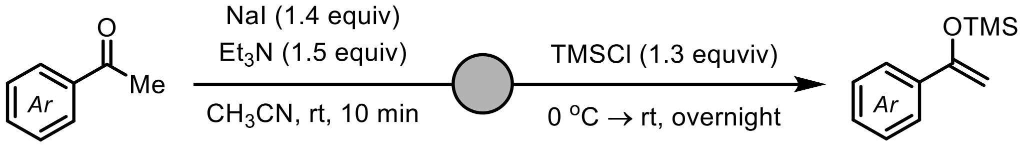

General procedure for the preparation of aryl silyl enol ethers:[1] NaI (210 mg, 1.4 mmol, 1.4 equiv) was placed in a tube and dried under vacuum using a heat gun. Upon cooling to room temperature, the tube was filled with argon. Then, dry CH_3CN (1.0 mL), ketone (1 mmol, 1.0 equiv), and Et_3N (210 µL, 1.5 mmol, 1.5 equiv) were successively added. The mixture was cooled with an ice/water bath, and TMSCl (166 µL, 1.3 mmol, 1.3 equiv) was added at 0 °C. The cooling bath was removed, and the mixture was stirred at room temperature for 12 h. Then, the volatile components were evaporated under vacuum. The solid residue was washed with petroleum ether (3 × 15 mL), the petroleum ether layers were decanted and filtered through a cotton plug. The combined filtrates were concentrated on a rotary evaporator, furnishing the silyl enol ether which was used without further purification.

NOTE: All active methylene compounds are commercially available.

III. Synthesis and analytical data of compounds 3, 4 ,5 and 6

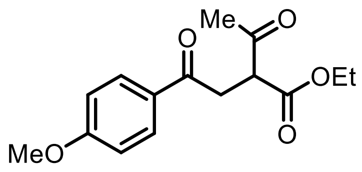

Ethyl 2-acetyl-4-(4-methoxyphenyl)-4-oxobutanoate (3a)[2]

To a 10 mL Schlenk tube equipped with a magnetic stir bar was added ((1-(4-methoxyphenyl)vinyl)oxy)trimethylsilane **1a** (222 mg, 1 mmol), ethyl acetoacetate **2a** (63 μL, 0.5 mmol), Ag_2O (23 mg, 0.1 mmol), and 1,4-dioxane (2.0 mL). The reaction mixture was stirred at room temperature for about 6 h. The resulting mixture was concentrated and the residue was taken up in ethyl acetate. The organic layer was washed with brine, dried over Na_2SO_4 and concentrated. Purification of the crude product by column chromatography (silica gel; petroleum ether/ethyl acetate 10:1; R_f = 0.18) afforded **3a** in 87% yield (121 mg).

Gram-scale synthesis of ethyl 2-acetyl-4-(4-methoxyphenyl)-4-oxobutanoate (3a)

To a 50 mL Schlenk tube equipped with a magnetic stir bar was added ((1-(4-methoxyphenyl)vinyl)oxy)trimethylsilane **1a** (2.23 g, 10 mmol), ethyl acetoacetate **2a** (630 μL, 5 mmol), Ag_2O (230 mg, 1 mmol), and 1,4-dioxane (20.0 mL). The reaction mixture was stirred at room temperature for about 12 h. The resulting mixture was concentrated and the residue was taken up in ethyl acetate. The organic layer was washed with brine, dried over Na_2SO_4 and concentrated. Purification of the crude product by column chromatography (silica gel; petroleum ether/ethyl acetate 10:1; R_f = 0.18) afforded **3a** in 73% yield (1.01 g).

Colorless oil; ^{1}H NMR ($CDCl_3$, 400 MHz): δ_H 7.96 (d, *J* = 9.2 Hz, 2H), 6.96 (d, *J* = 6.8 Hz, 2H), 4.25–4.20 (m, 3H), 3.87 (s, 3H), 3.71–3.64 (m, 1H), 3.51–3.45 (m, 1H), 2.44 (s, 3H), 1.30 (t, *J* = 7.2 Hz, 3H); $^{13}C\{^1H\}$ NMR ($CDCl_3$, 101 MHz): δ_C 202.6, 195.6, 169.0, 163.8, 130.4, 129.2, 113.8, 61.7, 55.5, 53.9, 37.1, 30.3, 14.0; HRMS (ESI-TOF, *m/z*): calcd for $C_{15}H_{19}O_5$ $[M + H]^+$, 279.1227; found, 279.1229; IR νmax (film, cm^{-1}): 3053, 2960, 1742, 1719, 1679, 1600, 1575, 1421, 1251, 1217, 1084, 1026, 832.

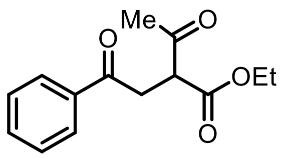

Ethyl 2-acetyl-4-oxo-4-phenylbutanoate (3b)[2]

To a 10 mL Schlenk tube equipped with a magnetic stir bar was added trimethyl((1-phenylvinyl)oxy)silane **1b** (192 mg, 1 mmol), ethyl acetoacetate **2a** (63 µL, 0.5 mmol), Ag_2O (23 mg, 0.1 mmol), and 1,4-dioxane (2.0 mL). The reaction mixture was stirred at room temperature for about 6 h. The resulting mixture was concentrated and the residue was taken up in ethyl acetate. The organic layer was washed with brine, dried over Na_2SO_4 and concentrated. Purification of the crude product by column chromatography (silica gel; petroleum ether/ethyl acetate 10:1; R_f = 0.25) afforded **3b** in 82% yield (102 mg).

Colorless oil; ^{1}H NMR ($CDCl_3$, 400 MHz): δ_H 7.98 (d, J = 7.6 Hz, 2H), 7.60–7.57 (m, 1H), 7.50–7.46 (m, 2H), 4.26-4.21 (m, 3H), 3.76–3.70 (m, 1H), 3.56–3.50 (m, 1H), 2.45 (s, 3H), 1.30 (t, J = 7.2 Hz, 3H); ^{13}C{^{1}H} NMR ($CDCl_3$, 101 MHz): δ_C 202.4, 197.1, 168.9, 136.0, 133.5, 128.6, 128.1, 61.7, 53.8, 37.4, 30.3, 14.0; HRMS (ESI-TOF, *m/z*): calcd for $C_{14}H_{17}O_4$ [M + H]$^+$, 249.1121; found, 249.1125; IR ν max (film, cm^{-1}): 2984, 2927, 1741, 1716, 1686, 1597, 1581, 1449, 1360, 1263, 1174, 1022, 755, 691.

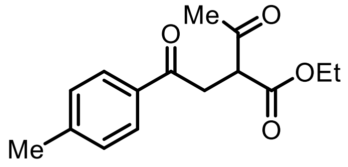

Ethyl 2-acetyl-4-oxo-4-(*p*-tolyl)butanoate (3c)[2]

To a 10 mL Schlenk tube equipped with a magnetic stir bar was added trimethyl((1-(*p*-tolyl)vinyl)oxy)silane **1c** (206 mg, 1 mmol), ethyl acetoacetate **2a** (63 µL, 0.5 mmol), Ag_2O (23 mg, 0.1 mmol), and 1,4-dioxane (2.0 mL). The reaction mixture was stirred at room temperature for about 6 h. The resulting mixture was concentrated and the residue was taken up in ethyl acetate. The organic layer was washed with brine, dried over Na_2SO_4 and concentrated. Purification of the crude product by column chromatography (silica gel; petroleum ether/ethyl acetate 10:1; R_f = 0.27) afforded **3c** in 82% yield (108 mg).

Colorless oil; ^{1}H NMR ($CDCl_3$, 400 MHz): δ_H 7.88 (d, J = 6.0 Hz, 2H), 7.26 (d, J = 6.0 Hz, 2H), 4.26–4.20 (m, 3H), 3.73–3.67 (m, 1H), 3.54–3.47 (m, 1H), 2.44 (s, 3H), 2.41 (s, 3H), 1.29 (t, J = 8.0 Hz, 3H); ^{13}C{^{1}H}

NMR ($CDCl_3$, 101 MHz): δ_C 202.5, 196.7, 168.9, 144.3, 133.6, 129.3, 128.2, 61.7, 53.9, 37.3, 30.3, 21.6, 14.0; HRMS (ESI-TOF, *m/z*): calcd for $C_{15}H_{19}O_4$ $[M + H]^+$, 263.1278; found, 263.1281; IR νmax (film, cm^{-1}): 2927, 1733, 1711, 1690, 1611, 1527, 1477, 1349, 1267, 1186, 1105, 801.

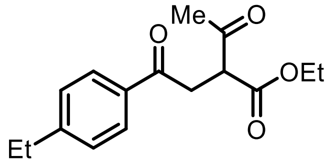

Ethyl 2-acetyl-4-(4-ethylphenyl)-4-oxobutanoate (3d)

To a 10 mL Schlenk tube equipped with a magnetic stir bar was added ((1-(4-ethylphenyl)vinyl)oxy)trimethylsilane **1d** (220 mg, 1 mmol), ethyl acetoacetate **2a** (63 µL, 0.5 mmol), Ag_2O (23 mg, 0.1 mmol), and 1,4-dioxane (2.0 mL). The reaction mixture was stirred at room temperature for about 6 h. The resulting mixture was concentrated and the residue was taken up in ethyl acetate. The organic layer was washed with brine, dried over Na_2SO_4 and concentrated. Purification of the crude product by column chromatography (silica gel; petroleum ether/ethyl acetate 10:1; R_f = 0.24) afforded **3d** in 81% yield (112 mg).

Colorless oil; ^1H NMR ($CDCl_3$, 400 MHz): δ_H 7.91 (d, *J* = 8.0 Hz, 2H), 7.30–7.27 (m, 2H), 4.25–4.20 (m, 3H), 3.73–3.67 (m, 1H), 3.54–3.48 (m, 1H), 2.71 (q, *J* = 7.6 Hz, 2H), 2.44 (s, 3H), 1.31–1.24 (m, 6H); $^{13}C\{^1H\}$ NMR ($CDCl_3$, 101 MHz): δ_C 202.5, 196.8, 169.0, 150.5, 133.8, 128.3, 128.1, 61.7, 53.9, 37.3, 30.3, 28.9, 15.1, 14.0; HRMS (ESI-TOF, *m/z*): calcd for $C_{16}H_{21}O_4$ $[M + H]^+$, 277.1434; found, 277.1441; IR ν max (film, cm^{-1}): 2969, 2934, 1744, 1717, 1682, 1607, 1570, 1462, 1359, 1266, 1174, 1023, 831.

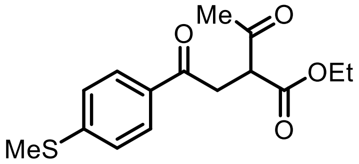

Ethyl 2-acetyl-4-(4-(methylthio)phenyl)-4-oxobutanoate (3e)

To a 10 mL Schlenk tube equipped with a magnetic stir bar was added trimethyl((1-(4-(methylthio)phenyl)vinyl)oxy)silane **1e** (238 mg, 1 mmol), ethyl acetoacetate **2a** (63 µL, 0.5 mmol), Ag_2O (23 mg, 0.1 mmol), and 1,4-dioxane (2.0 mL). The reaction mixture was stirred at room temperature for about 6 h. The resulting mixture was concentrated and the residue was taken up in

ethyl acetate. The organic layer was washed with brine, dried over Na_2SO_4 and concentrated. Purification of the crude product by column chromatography (silica gel; petroleum ether/ethyl acetate 10:1; R_f = 0.22) afforded **3e** in 71% yield (105 mg).

Colorless oil; ^{1}H NMR ($CDCl_3$, 400 MHz): δ_H 7.88 (d, J = 8.0 Hz, 2H), 7.26 (d, J = 8.0 Hz, 2H), 4.25–4.20 (m, 3H), 3.70–3.64 (m, 1H), 3.51–3.45 (m, 1H), 2.52 (s, 3H), 2.44 (s, 3H), 1.29 (t, J = 7.2 Hz, 3H); ^{13}C{^{1}H} NMR ($CDCl_3$, 101 MHz): δ_C 202.4, 196.1, 168.9, 146.5, 132.3, 128.5, 124.9, 61.7, 53.8, 37.1, 30.3, 14.7, 14.0; HRMS (ESI-TOF, *m/z*): calcd for $C_{15}H_{19}O_4S$ [M + H]$^+$, 295.0999; found, 295.0998; IR ν max (film, cm^{-1}): 2983, 2923, 1740, 1717, 1677, 1590, 1557, 1490, 1358, 1263, 1210, 1093, 819.

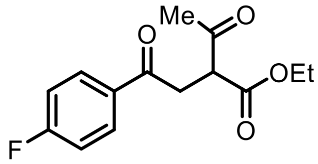

Ethyl 2-acetyl-4-(4-fluorophenyl)-4-oxobutanoate (3f)[2]

To a 10 mL Schlenk tube equipped with a magnetic stir bar was added ((1-(4-fluorophenyl)vinyl)oxy)trimethylsilane **1f** (210 mg, 1 mmol), ethyl acetoacetate **2a** (63 µL, 0.5 mmol), Ag_2O (23 mg, 0.1 mmol), and 1,4-dioxane (2.0 mL). The reaction mixture was stirred at room temperature for about 6 h. The resulting mixture was concentrated and the residue was taken up in ethyl acetate. The organic layer was washed with brine, dried over Na_2SO_4 and concentrated. Purification of the crude product by column chromatography (silica gel; petroleum ether/ethyl acetate 10:1; R_f = 0.24) afforded **3f** in 74% yield (99 mg).

Colorless oil; ^{1}H NMR ($CDCl_3$, 400 MHz): δ_H 8.03–8.00 (m, 2H), 7.16–7.12 (m, 2H), 4.26–4.20 (m, 3H), 3.72–3.65 (m, 1H), 3.51–3.45 (m, 1H), 2.45 (s, 3H), 1.30 (t, J = 7.2 Hz, 3H); ^{13}C{^{1}H} NMR ($CDCl_3$, 101 MHz): δ_C 202.3, 195.6, 168.8, 166.0 (d, J_{C-F} = 253.9 Hz), 132.5 (d, J_{C-F} = 2.5 Hz), 130.8 (d, J_{C-F} = 9.2 Hz), 115.8 (d, J_{C-F} = 21.8 Hz), 61.8, 53.9, 37.2, 30.3, 14.0; ^{19}F NMR ($CDCl_3$, 376 MHz): δ_F –104.4; HRMS (ESI-TOF, *m/z*): calcd for $C_{14}H_{16}FO_4$ [M + H]$^+$, 267.1027; found, 267.1028; IR ν max (film, cm^{-1}): 2981, 2931, 1737, 1718, 1683, 1594, 1507, 1472, 1365, 1270, 1209, 1176, 1022, 859.

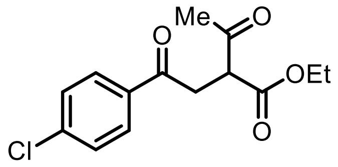

Ethyl 2-acetyl-4-(4-chlorophenyl)-4-oxobutanoate (3g)[2]

To a 10 mL Schlenk tube equipped with a magnetic stir bar was added ((1-(4-chlorophenyl)vinyl)oxy)trimethylsilane **1g** (226 mg, 1 mmol), ethyl acetoacetate **2a** (63 µL, 0.5 mmol), Ag_2O (23 mg, 0.1 mmol), and 1,4-dioxane (2.0 mL). The reaction mixture was stirred at room temperature for about 6 h. The resulting mixture was concentrated and the residue was taken up in ethyl acetate. The organic layer was washed with brine, dried over Na_2SO_4 and concentrated. Purification of the crude product by column chromatography (silica gel; petroleum ether/ethyl acetate 10:1; R_f = 0.25) afforded **3g** in 86% yield (122 mg).

White solid; mp 57.6–58.6 °C; ^{1}H NMR ($CDCl_3$, 400 MHz): δ_H 7.92 (d, J = 7.6 Hz, 2H), 7.45 (d, J = 7.6 Hz, 2H), 4.26–4.20 (m, 3H), 3.71–3.65 (m, 1H), 3.50–3.44 (m, 1H), 2.44 (s, 3H), 1.30 (t, J = 7.2 Hz, 3H); ^{13}C{^{1}H} NMR ($CDCl_3$, 101 MHz): δ_C 202.2, 196.0, 168.7, 140.0, 134.4, 129.5, 129.0, 61.9, 53.8, 37.3, 30.3, 14.0; HRMS (ESI-TOF, *m/z*): calcd for $C_{14}H_{16}ClO_4$ [M + H]$^+$, 283.0732; found, 283.0736; IR ν max (KBr, cm^{-1}): 3092, 2978, 2936, 1737, 1718, 1685, 1588, 1570, 1489, 1364, 1268, 1177, 1089, 856.

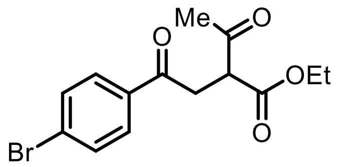

Ethyl 2-acetyl-4-(4-bromophenyl)-4-oxobutanoate (3h)[3]

To a 10 mL Schlenk tube equipped with a magnetic stir bar was added ((1-(4-bromophenyl)vinyl)oxy)trimethylsilane **1h** (270 mg, 1 mmol), ethyl acetoacetate **2a** (63 µL, 0.5 mmol), Ag_2O (23 mg, 0.1 mmol), and 1,4-dioxane (2.0 mL). The reaction mixture was stirred at room temperature for about 6 h. The resulting mixture was concentrated and the residue was taken up in ethyl acetate. The organic layer was washed with brine, dried over Na_2SO_4 and concentrated. Purification of the crude product by column chromatography (silica gel; petroleum ether/ethyl acetate 10:1; R_f = 0.23) afforded **3h** in 82% yield (134 mg).

White solid; mp 58.7–59.7 °C; ^{1}H NMR ($CDCl_3$, 400 MHz): δ_H 7.84 (d, J = 8.0 Hz, 2H), 7.62 (d, J = 8.0 Hz,

2H), 4.26–4.20 (m, 3H), 3.71–3.64 (m, 1H), 3.50–3.44 (m, 1H), 2.44 (s, 3H), 1.30 (t, J = 7.2 Hz, 3H); $^{13}C\{^{1}H\}$ NMR ($CDCl_3$, 101 MHz): δ_C 202.2, 196.2, 168.7, 134.8, 132.0, 129.6, 128.7, 61.9, 53.8, 37.3, 30.3, 14.0; HRMS (ESI-TOF, *m/z*): calcd for $C_{14}H_{16}BrO_4$ $[M + H]^+$, 327.0226; found, 327.0234; IR ν max (KBr, cm^{-1}): 2978, 2935, 1736, 1717, 1686, 1584, 1567, 1486, 1400, 1267, 1177, 1021, 862.

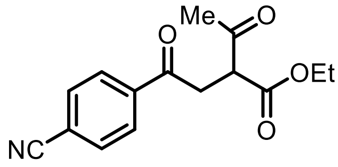

Ethyl 2-acetyl-4-(4-cyanophenyl)-4-oxobutanoate (3i)[4]

To a 10 mL Schlenk tube equipped with a magnetic stir bar was added 4-(1-((trimethylsilyl)oxy)vinyl)benzonitrile **1i** (217 mg, 1 mmol), ethyl acetoacetate **2a** (63 µL, 0.5 mmol), Ag_2O (23 mg, 0.1 mmol), and 1,4-dioxane (2.0 mL). The reaction mixture was stirred at room temperature for about 6 h. The resulting mixture was concentrated and the residue was taken up in ethyl acetate. The organic layer was washed with brine, dried over Na_2SO_4 and concentrated. Purification of the crude product by column chromatography (silica gel; petroleum ether/ethyl acetate 10:1; R_f = 0.27) afforded **3i** in 63% yield (86 mg).

White solid; mp 95.9–96.9 °C; ^{1}H NMR ($CDCl_3$, 400 MHz): δ_H 8.07 (d, J = 8.0 Hz, 2H), 7.79 (d, J = 8.0 Hz, 2H), 4.27–4.22 (m, 3H), 3.74–3.68 (m, 1H), 3.52–3.46 (m, 1H), 2.45 (s, 3H), 1.31 (t, J = 7.2 Hz, 3H); $^{13}C\{^{1}H\}$ NMR ($CDCl_3$, 101 MHz): δ_C 201.9, 196.0, 168.5, 139.0, 132.5, 128.5, 117.8, 116.7, 62.0, 53.8, 37.4, 30.2, 14.0; HRMS (ESI-TOF, *m/z*): calcd for $C_{15}H_{16}NO_4$ $[M + H]^+$, 274.1074; found, 274.1076; IR ν max (KBr, cm^{-1}): 2987, 2923, 2230, 1736, 1718, 1686, 1606, 1568, 1470, 1321, 1264, 1201, 1182, 1016, 850.

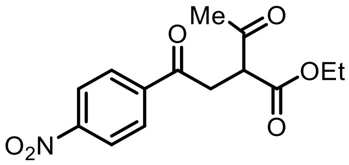

Ethyl 2-acetyl-4-(4-nitrophenyl)-4-oxobutanoate (3j)[3]

To a 10 mL Schlenk tube equipped with a magnetic stir bar was added trimethyl((1-(4-nitrophenyl)vinyl)oxy)silane **1j** (237 mg, 1 mmol), ethyl acetoacetate **2a** (63 µL, 0.5 mmol), Ag_2O (23 mg, 0.1 mmol), and 1,4-dioxane (2.0 mL). The reaction mixture was stirred at room temperature for about 6 h. The resulting mixture was concentrated and the residue was taken up in ethyl acetate. The organic layer was washed with brine, dried over Na_2SO_4 and concentrated. Purification of the crude product by column chromatography (silica gel; petroleum ether/ethyl acetate 10:1; R_f = 0.24) afforded **3j** in 77% yield (113 mg).

Yellow oil; ^{1}H NMR ($CDCl_3$, 400 MHz): δ_H 8.33 (d, J = 8.4 Hz, 2H), 8.14 (d, J = 8.4 Hz, 2H), 4.28–4.23 (m, 3H), 3.78–3.71 (m, 1H), 3.55–3.49 (m, 1H), 2.46 (s, 3H), 1.31 (t, J = 7.2 Hz, 3H); $^{13}C\{^1H\}$ NMR ($CDCl_3$, 101 MHz): δ_C 201.8, 195.8, 168.5, 150.5, 140.5, 129.2, 123.9, 62.0, 53.9, 38.7, 30.2, 14.0; HRMS (ESI-TOF, *m/z*): calcd for $C_{14}H_{16}NO_6$ $[M + H]^+$, 294.0972; found, 294.0985; IR νmax (film, cm^{-1}): 2984, 2924, 1740, 1719, 1683, 1607, 1574, 1447, 1359, 1324, 1263, 1178, 1023, 816.

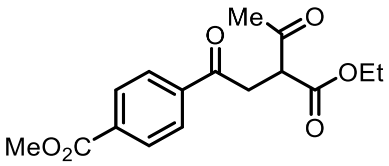

Methyl 4-(3-(ethoxycarbonyl)-4-oxopentanoyl)benzoate (3k)

To a 10 mL Schlenk tube equipped with a magnetic stir bar was added methyl methyl 4-(1-((trimethylsilyl)oxy)vinyl)benzoate **1k** (250 mg, 1 mmol), ethyl acetoacetate **2a** (63 µL, 0.5 mmol), Ag_2O (23 mg, 0.1 mmol), and 1,4-dioxane (2.0 mL). The reaction mixture was stirred at room temperature for about 6 h. The resulting mixture was concentrated and the residue was taken up in ethyl acetate. The organic layer was washed with brine, dried over Na_2SO_4 and concentrated. Purification of the crude product by column chromatography (silica gel; petroleum ether/ethyl acetate 10:1; R_f = 0.26) afforded **3k** in 78% yield (120 mg).

White solid; mp 57.4–58.4 °C; ^{1}H NMR ($CDCl_3$, 400 MHz): δ_H 8.13 (d, J = 7.8 Hz, 2H), 8.03 (d, J = 7.8 Hz,

2H), 4.27–4.21 (m, 3H), 3.96 (s, 3H), 3.77–3.70 (m, 1H) 3.56–3.50 (m, 1H), 2.45 (s, 3H), 1.30 (t, J = 7.2 Hz, 3H); $^{13}C\{^1H\}$ NMR ($CDCl_3$, 101 MHz): δ_C 202.1, 196.7, 168.7, 166.1, 139.2, 134.2, 129.9, 128.1, 61.9, 53.8, 52.5, 37.6, 30.3, 14.0; HRMS (ESI-TOF, *m/z*): calcd for $C_{16}H_{19}O_6$ $[M + H]^+$, 307.1176; found, 307.1182; IR ν max (KBr, cm^{-1}): 2981, 2930, 1733, 1716, 1679, 1572, 1505, 1477, 1363, 1291, 1263, 1181, 1017, 867.

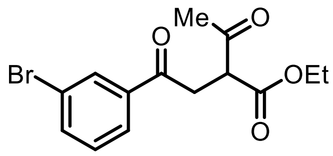

Ethyl 2-acetyl-4-(3-bromophenyl)-4-oxobutanoate (3l)[3]

To a 10 mL Schlenk tube equipped with a magnetic stir bar was added added ((1-(3-bromophenyl)vinyl)oxy)trimethylsilane **1l** (270 mg, 1 mmol), ethyl acetoacetate **2a** (63 µL, 0.5 mmol), Ag_2O (23 mg, 0.1 mmol), and 1,4-dioxane (2.0 mL). The reaction mixture was stirred at room temperature for about 6 h. The resulting mixture was concentrated and the residue was taken up in ethyl acetate. The organic layer was washed with brine, dried over Na_2SO_4 and concentrated. Purification of the crude product by column chromatography (silica gel; petroleum ether/ethyl acetate 10:1; R_f = 0.24) afforded **3l** in 74% yield (121 mg).

Colorless oil; ^1H NMR ($CDCl_3$, 400 MHz): δ_H 8.11 (s, 1H), 7.91 (d, J = 8.0 Hz, 1H), 7.71 (d, J = 7.6 Hz, 1H), 7.38–7.34 (m, 1H), 4.26–4.20 (m, 3H), 3.71–3.65 (m, 1H), 3.50–3.44 (m, 1H), 2.44 (s, 3H), 1.31 (t, J = 7.2 Hz, 3H); $^{13}C\{^1H\}$ NMR ($CDCl_3$, 101 MHz): δ_C 202.1, 195.9, 168.7, 137.8, 136.3, 131.2, 130.3, 126.7, 123.0, 61.9, 53.8, 37.3, 30.2, 14.0; HRMS (ESI-TOF, *m/z*): calcd for $C_{14}H_{16}BrO_4$ $[M + H]^+$, 327.0226; found, 327.0234; IR ν max (film, cm^{-1}): 2979, 2930, 1737, 1717, 1687, 1589, 1563, 1471, 1407, 1363, 1267, 1208, 1176, 1022, 792, 683.

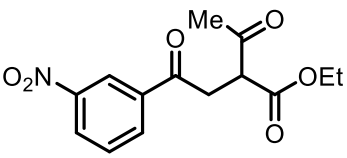

Ethyl 2-acetyl-4-(3-nitrophenyl)-4-oxobutanoate (3m)

To a 10 mL Schlenk tube equipped with a magnetic stir bar was added trimethyl((1-(3-nitrophenyl)vinyl)oxy)silane **1m** (237 mg, 1 mmol), ethyl acetoacetate **2a** (63 µL, 0.5 mmol), Ag_2O (23 mg, 0.1 mmol), and 1,4-dioxane (2.0 mL). The reaction mixture was stirred at room temperature for about 6 h. The resulting mixture was concentrated and the residue was taken up in ethyl acetate. The organic layer was washed with brine, dried over Na_2SO_4 and concentrated. Purification of the crude product by column chromatography (silica gel; petroleum ether/ethyl acetate 10:1; R_f = 0.26) afforded **3m** in 70% yield (103 mg).

Yellow oil; ^{1}H NMR ($CDCl_3$, 400 MHz): δ_H 8.81 (s, 1H), 8.45 (d, J = 8.4 Hz, 1H), 8.31 (d, J = 7.6 Hz, 1H), 7.72–7.68 (m, 1H), 4.28–4.23 (m, 3H), 3.79–3.72 (m, 1H), 3.56–3.50 (m, 1H), 2.46 (s, 3H), 1.32 (t, J = 7.2 Hz, 3H); $^{13}C\{^1H\}$ NMR ($CDCl_3$, 101 MHz): δ_C 201.9, 195.2, 168.5, 148.5, 137.3, 133.7, 130.0, 127.7, 123.1, 62.0, 53.9, 37.4, 30.2, 14.0; HRMS (ESI-TOF, *m/z*): calcd for $C_{14}H_{16}NO_6$ [M + H]$^+$, 294.0972; found, 294.0985; IR νmax (film, cm^{-1}): 2993, 2927, 1733, 1711, 1690, 1611, 1527, 1477, 1399, 1349, 1267, 1185, 1150, 1015, 735, 675.

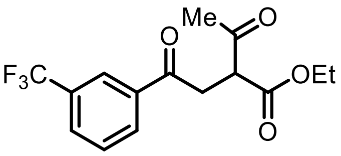

Ethyl 2-acetyl-4-oxo-4-(3-(trifluoromethyl)phenyl)butanoate (3n)

To a 10 mL Schlenk tube equipped with a magnetic stir bar was added trimethyl((1-(3-(trifluoromethyl)phenyl)vinyl)oxy)silane **1n** (260 mg, 1 mmol), ethyl acetoacetate **2a** (63 µL, 0.5 mmol), Ag_2O (23 mg, 0.1 mmol), and 1,4-dioxane (2.0 mL). The reaction mixture was stirred at room temperature for about 6 h. The resulting mixture was concentrated and the residue was taken up in ethyl acetate. The organic layer was washed with brine, dried over Na_2SO_4 and concentrated. Purification of the crude product by column chromatography (silica gel; petroleum ether/ethyl acetate 10:1; R_f = 0.22) afforded **3n** in 62% yield (98 mg).

White solid; mp 63.8–64.8 °C; ^{1}H NMR ($CDCl_3$, 400 MHz): δ_H 8.23 (s, 1H), 8.17 (d, J = 8.0 Hz, 1H), 7.87–

7.83 (m, 1H), 7.67–7.61 (m, 1H), 4.27–4.22 (m, 3H), 3.72–3.70 (m, 1H), 3.55–3.50 (m, 1H), 2.46 (s, 3H), 1.31 (t, J = 7.2 Hz, 3H); $^{13}C\{^{1}H\}$ NMR ($CDCl_3$, 101 MHz): δ_C 202.0, 195.9, 168.6, 137.1 (d, $J_{C–F}$ = 52.6 Hz), 131.4 (d, $J_{C–F}$ = 32.8 Hz), 131.3, 129.9 (d, $J_{C–F}$ = 3.5 Hz), 129.7 (d, $J_{C–F}$ = 3.4 Hz), 129.4, 125.0 (d, $J_{C–F}$ = 3.7 Hz), 122.2, 61.9, 53.8, 37.3, 30.2, 14.0; ^{19}F NMR ($CDCl_3$, 376 MHz): δ_F –62.75, –62.80; HRMS (ESI-TOF, *m/z*): calcd for $C_{15}H_{16}F_3O_4$ $[M + H]^+$, 317.0995; found, 317.0990; IR νmax (KBr, cm^{-1}): 3001, 2929, 1739, 1718, 1686, 1613, 1437, 1366, 1335, 1269 1165, 1125, 1072, 806, 696.

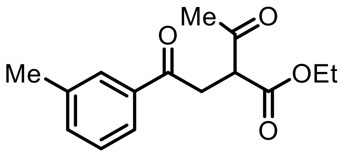

Ethyl 2-acetyl-4-oxo-4-(*m*-tolyl)butanoate (3o)[5]

To a 10 mL Schlenk tube equipped with a magnetic stir bar was added trimethyl((1-(*m*-tolyl)vinyl)oxy)silane **1o** (206 mg, 1 mmol), ethyl acetoacetate **2a** (63 µL, 0.5 mmol), Ag_2O (23 mg, 0.1 mmol), and 1,4-dioxane (2.0 mL). The reaction mixture was stirred at room temperature for about 6 h. The resulting mixture was concentrated and the residue was taken up in ethyl acetate. The organic layer was washed with brine, dried over Na_2SO_4 and concentrated. Purification of the crude product by column chromatography (silica gel; petroleum ether/ethyl acetate 10:1; R_f = 0.28) afforded **3o** in 83% yield (109 mg).

Colorless oil; ^{1}H NMR ($CDCl_3$, 400 MHz): δ_H 7.79–7.77 (m, 2H), 7.40–7.33 (m, 2H), 4.26–4.20 (m, 3H), 3.74–3.67 (m, 1H), 3.54–3.48 (m, 1H), 2.44 (s, 3H), 2.41 (s, 3H), 1.30 (t, J = 7.2 Hz, 3H); $^{13}C\{^{1}H\}$ NMR ($CDCl_3$, 101 MHz): δ_C 202.4, 197.3, 168.9, 138.4, 136.1, 134.2, 128.7, 128.5, 125.3, 61.7, 53.9, 37.4, 30.3, 21.3, 14.0; HRMS (ESI-TOF, *m/z*): calcd for $C_{15}H_{19}O_4$ $[M + H]^+$, 263.1278; found, 263.1281; IR ν max (film, cm^{-1}): 2979, 2924, 1741, 1717, 1684, 1604, 1586, 1403, 1359, 1267, 1158, 1038, 786, 691.

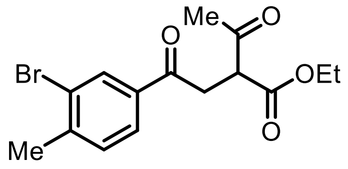

Ethyl 2-acetyl-4-(3-bromo-4-methylphenyl)-4-oxobutanoate (3p)

To a 10 mL Schlenk tube equipped with a magnetic stir bar was added ((1-(3-bromo-4-methylphenyl)vinyl)oxy)trimethylsilane **1p** (284 mg, 1 mmol), ethyl acetoacetate **2a** (63 µL, 0.5 mmol), Ag_2O (23 mg, 0.1 mmol), and 1,4-dioxane (2.0 mL). The reaction mixture was stirred at room temperature for about 6 h. The resulting mixture was concentrated and the residue was taken up in ethyl acetate. The organic layer was washed with brine, dried over Na_2SO_4 and concentrated. Purification of the crude product by column chromatography (silica gel; petroleum ether/ethyl acetate 10:1; R_f = 0.23) afforded **3p** in 87% yield (148 mg).

White solid; mp 69.2–70.2 °C; ^{1}H NMR ($CDCl_3$, 400 MHz): δ_H 8.13 (s, 1H), 7.81 (d, *J* = 8.0 Hz, 1H), 7.33 (d, *J* = 8.0 Hz, 1H), 4.26–4.19 (m, 3H), 3.69–3.63 (m, 1H), 3.49–3.43 (m, 1H), 2.46 (s, 3H), 2.44 (s, 3H), 1.30 (t, *J* = 6.8 Hz, 3H); ^{13}C{^{1}H} NMR ($CDCl_3$, 101 MHz): δ_C 202.2, 195.6, 168.8, 144.0, 135.4, 132.1, 130.9, 126.9, 125.3, 61.8, 53.8, 37.2, 30.2, 23.2, 14.0; HRMS (ESI-TOF, *m/z*): calcd for $C_{15}H_{18}BrO_4$ [M + H]$^+$, 341.0383; found, 341.0392; IR ν max (KBr, cm^{-1}): 2992, 2975, 2935, 1740, 1717, 1683, 1598, 1474, 1401, 1361, 1262, 1207, 1175, 1146, 1036, 790, 680, 607.

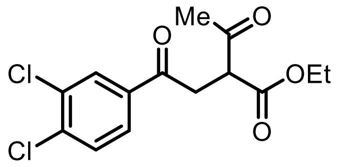

Ethyl 2-acetyl-4-(3,4-dichlorophenyl)-4-oxobutanoate (3q)[3]

To a 10 mL Schlenk tube equipped with a magnetic stir bar was added ((1-(3,4-dichlorophenyl)vinyl)oxy)trimethylsilane **1q** (260 mg, 1 mmol), ethyl acetoacetate **2a** (63 µL, 0.5 mmol), Ag_2O (23 mg, 0.1 mmol), and 1,4-dioxane (2.0 mL). The reaction mixture was stirred at room temperature for about 6 h. The resulting mixture was concentrated and the residue was taken up in ethyl acetate. The organic layer was washed with brine, dried over Na_2SO_4 and concentrated. Purification of the crude product by column chromatography (silica gel; petroleum ether/ethyl acetate 10:1; R_f = 0.21) afforded **3q** in 80% yield (127 mg).

White solid; mp 59.4–60.4 °C; ^{1}H NMR ($CDCl_3$, 400 MHz): δ_H 8.06 (s, 1H), 7.81 (d, J = 8.4 Hz, 1H), 7.56 (d, J = 8.4 Hz, 1H), 4.26–4.19 (m, 3H), 3.69–3.62 (m, 1H), 3.47–3.41 (m, 1H), 2.44 (s, 3H), 1.31 (t, J = 6.8 Hz, 3H); $^{13}C\{^1H\}$ NMR ($CDCl_3$, 101 MHz): δ_C 202.0, 195.1, 168.6, 138.1, 135.6, 133.4, 130.8, 130.2, 127.1, 61.9, 53.8, 37.2, 30.2, 14.0; HRMS (ESI-TOF, *m/z*): calcd for $C_{14}H_{15}Cl_2O_4$ [M + H]$^+$, 317.0342; found, 317.0344; IR ν max (KBr, cm^{-1}): 2977, 2932, 1739, 1716, 1686, 1585, 1557, 1472, 1380, 1259, 1178, 1145, 1028, 856, 804.

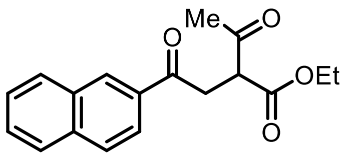

Ethyl 2-acetyl-4-(naphthalen-2-yl)-4-oxobutanoate (3r)[3]

To a 10 mL Schlenk tube equipped with a magnetic stir bar was added trimethyl((1-(naphthalen-2-yl)vinyl)oxy)silane **1r** (242 mg, 1 mmol), ethyl acetoacetate **2a** (63 µL, 0.5 mmol), Ag_2O (23 mg, 0.1 mmol), and 1,4-dioxane (2.0 mL). The reaction mixture was stirred at room temperature for about 6 h. The resulting mixture was concentrated and the residue was taken up in ethyl acetate. The organic layer was washed with brine, dried over Na_2SO_4 and concentrated. Purification of the crude product by column chromatography (silica gel; petroleum ether/ethyl acetate 10:1; R_f = 0.29) afforded **3r** in 84% yield (126 mg).

Colorless oil; ^{1}H NMR ($CDCl_3$, 400 MHz): δ_H 8.54 (s, 1H), 8.02–7.97 (m, 2H), 7.91–7.87 (m, 2H), 7.63–7.55 (m, 2H), 4.31–4.23 (m, 3H), 3.90–3.83 (m, 1H), 3.70–3.64 (m, 1H), 2.48 (s, 3H), 1.31 (t, J = 7.2 Hz, 3H); $^{13}C\{^1H\}$ NMR ($CDCl_3$, 101 MHz): δ_C 202.5, 197.1, 169.0, 135.8, 133.4, 132.5, 130.1, 129.6, 128.7, 128.5, 127.8, 126.9, 123.6, 61.8, 54.0, 37.4, 30.3, 14.0; HRMS (ESI-TOF, *m/z*): calcd for $C_{18}H_{19}O_4$ [M + H]$^+$, 299.1278; found, 299.1277; IR νmax (film, cm^{-1}): 2977, 2922, 1737, 1714, 1681, 1601, 1472, 1402, 1362, 1264, 1208, 1171, 1019, 840, 869, 813, 789.

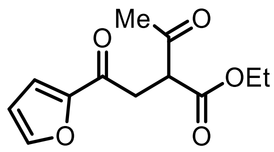

Ethyl 2-acetyl-4-(furan-2-yl)-4-oxobutanoate (3s)[3]

To a 10 mL Schlenk tube equipped with a magnetic stir bar was added ((1-(furan-2-yl)vinyl)oxy)trimethylsilane **1s** (182 mg, 1 mmol), ethyl acetoacetate **2a** (63 µL, 0.5 mmol), Ag_2O (23 mg, 0.1 mmol), and 1,4-dioxane (2.0 mL). The reaction mixture was stirred at room temperature for about 6 h. The resulting mixture was concentrated and the residue was taken up in ethyl acetate. The organic layer was washed with brine, dried over Na_2SO_4 and concentrated. Purification of the crude product by column chromatography (silica gel; petroleum ether/ethyl acetate 10:1; R_f = 0.24) afforded **3s** in 70% yield (84 mg).

Colorless oil; ^{1}H NMR ($CDCl_3$, 400 MHz): δ_H 7.80 (d, *J* = 3.6 Hz, 1H), 7.66 (d, *J* = 4.8 Hz, 1H), 7.16–7.14 (m, 1H), 4.25–4.20 (m, 3H), 3.69–3.63 (m, 1H), 3.50–3.44 (m, 1H), 2.43 (s, 3H), 1.29 (t, *J* = 7.2 Hz, 3H); ^{13}C{^{1}H} NMR ($CDCl_3$, 101 MHz): δ_C 202.2, 190.0, 168.7, 143.0, 134.0, 132.5, 128.2, 61.8, 53.7, 37.6, 30.3, 14.0; HRMS (ESI-TOF, *m/z*): calcd for $C_{12}H_{15}O_5$ [M + H]$^+$, 239.0914; found, 239.0918; IR νmax (film, cm^{-1}): 2983, 2927, 1740, 1717, 1663, 1519, 1417, 1358, 1264, 1208, 1174, 1096, 1020, 729.

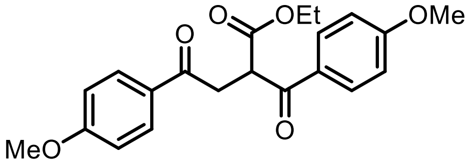

Ethyl 2-(4-methoxybenzoyl)-4-(4-methoxyphenyl)-4-oxobutanoate (3t)

To a 10 mL Schlenk tube equipped with a magnetic stir bar was added ((1-(4-methoxyphenyl)vinyl)oxy)trimethylsilane **1a** (222 mg, 1 mmol), ethyl 3-(4-methoxyphenyl)-3-oxopropanoate **2b** (96 mg, 0.5 mmol), Ag_2O (23 mg, 0.1 mmol), and 1,4-dioxane (2.0 mL). The reaction mixture was stirred at room temperature for about 6 h. The resulting mixture was concentrated and the residue was taken up in ethyl acetate. The organic layer was washed with brine, dried over Na_2SO_4 and concentrated. Purification of the crude product by column chromatography (silica gel; petroleum ether/ethyl acetate 10:1; R_f = 0.16) afforded **3t** in 82% yield (140 mg).

Colorless oil; ^{1}H NMR ($CDCl_3$, 400 MHz): δ_H 8.09 (d, *J* = 8.4 Hz, 2H), 7.98 (d, *J* = 8.4 Hz, 2H), 6.98–6.92 (m, 4H), 5.08 (t, *J* = 6.8 Hz, 1H), 4.16 (q, *J* = 7.2 Hz, 2H), 3.88 (s, 3H), 3.86 (s, 3H), 3.70 (d, *J* = 6.0 Hz, 2H),

1.18 (t, J = 7.2 Hz, 3H); $^{13}C\{^1H\}$ NMR ($CDCl_3$, 101 MHz): δ_C 195.4, 193.2, 169.5, 163.7, 131.3, 130.4, 129.2, 129.0, 113.8, 113.7, 61.6, 55.5, 48.6, 37.8, 13.9; HRMS (ESI-TOF, m/z): calcd for $C_{21}H_{23}O_6$ [M + H]$^+$, 371.1489; found, 371.1491; IR ν max (film, cm^{-1}): 2979, 2937, 2841, 1731, 1718, 1666, 1596, 1511, 1463, 1421, 1354, 1245, 1172, 1030, 838.

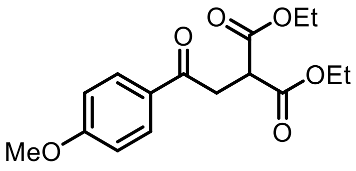

Diethyl 2-(2-(4-methoxyphenyl)-2-oxoethyl)malonate (3u)[6]

To a 10 mL Schlenk tube equipped with a magnetic stir bar was added ((1-(4-methoxyphenyl)vinyl)oxy)trimethylsilane **1a** (222 mg, 1 mmol), diethyl malonate **2c** (76 µL, 0.5 mmol), Ag_2O (23 mg, 0.1 mmol), and 1,4-dioxane (2.0 mL). The reaction mixture was stirred at room temperature for about 6 h. The resulting mixture was concentrated and the residue was taken up in ethyl acetate. The organic layer was washed with brine, dried over Na_2SO_4 and concentrated. Purification of the crude product by column chromatography (silica gel; petroleum ether/ethyl acetate 10:1; R_f = 0.22) afforded **3u** in 92% yield (142 mg).

Colorless oil; ^1H NMR ($CDCl_3$, 400 MHz): δ_H 7.97 (d, J = 8.0 Hz, 2H), 6.94 (d, J = 8.0 Hz, 2H), 4.25–4.22 (m, 4H), 4.05 (t, J = 6.8 Hz, 1H), 3.87 (s, 3H), 3.58 (d, J = 7.2 Hz, 2H), 1.29 (t, J = 7.2 Hz, 6H); $^{13}C\{^1H\}$ NMR ($CDCl_3$, 101 MHz): δ_C 195.0, 169.1, 163.7, 130.4, 129.2, 113.8, 61.7, 55.5, 47.2, 37.4, 14.0; HRMS (ESI-TOF, m/z): calcd for $C_{16}H_{21}O_6$ [M + H]$^+$, 309.1333; found, 309.1329; IR ν max (film, cm^{-1}): 2981, 2938, 1735, 1714, 1676, 1600, 1577, 1512, 1465, 1420, 1368, 1260, 1167, 1032, 835.

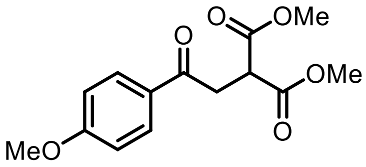

Dimethyl 2-(2-(4-methoxyphenyl)-2-oxoethyl)malonate (3v)[7]

To a 10 mL Schlenk tube equipped with a magnetic stir bar was added ((1-(4-methoxyphenyl)vinyl)oxy)trimethylsilane **1a** (222 mg, 1 mmol), dimethyl malonate **2d** (57 µL, 0.5 mmol), Ag_2O (23 mg, 0.1 mmol), and 1,4-dioxane (2.0 mL). The reaction mixture was stirred at room temperature for about 6 h. The resulting mixture was concentrated and the residue was taken up in ethyl acetate. The organic layer was washed with brine, dried over Na_2SO_4 and concentrated. Purification of the crude product by column chromatography (silica gel; petroleum ether/ethyl acetate 10:1; R_f = 0.21) afforded **3v** in 85% yield (119 mg).

Colorless oil; ^{1}H NMR ($CDCl_3$, 400 MHz): δ_H 7.96 (d, J = 8.4 Hz, 2H), 7.94 (d, J = 8.4 Hz, 2H), 4.08 (t, J = 7.2 Hz, 1H), 3.87 (s, 3H), 3.78 (s, 6H), 3.60 (d, J = 7.2 Hz, 2H); ^{13}C{^{1}H} NMR ($CDCl_3$, 101 MHz): δ_C 194.8, 169.5, 163.8, 130.4, 129.0, 113.8, 55.5, 52.8, 46.8, 37.5; HRMS (ESI-TOF, *m/z*): calcd for $C_{14}H_{17}O_6$ [M + H]$^+$, 281.1020; found, 281.1026; IR νmax (film, cm^{-1}): 2956, 2894, 1747, 1735, 1677, 1601, 1576, 1436, 1421, 1342, 1262, 1221, 1170, 1029, 833.

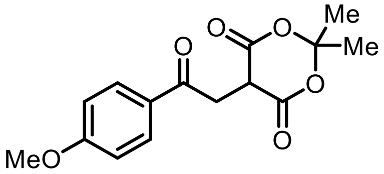

5-(2-(4-Methoxyphenyl)-2-oxoethyl)-2,2-dimethyl-1,3-dioxane-4,6-dione (3w)

To a 10 mL Schlenk tube equipped with a magnetic stir bar was added ((1-(4-methoxyphenyl)vinyl)oxy)trimethylsilane **1a** (222 mg, 1 mmol), 2,2-dimethyl-1,3-dioxane-4,6-dione **2e** (72 mg, 0.5 mmol), Ag_2O (23 mg, 0.1 mmol), and 1,4-dioxane (2.0 mL). The reaction mixture was stirred at room temperature for about 6 h. The resulting mixture was concentrated and the residue was taken up in ethyl acetate. The organic layer was washed with brine, dried over Na_2SO_4 and concentrated. Purification of the crude product by column chromatography (silica gel; petroleum ether/ethyl acetate 6:1; R_f = 0.16) afforded **3w** in 93% yield (136 mg).

White solid; mp 91.2–92.2 °C; ^{1}H NMR ($CDCl_3$, 400 MHz): δ_H 8.03 (d, J = 6.8 Hz, 2H), 6.95 (d, J = 6.8 Hz,

2H), 3.88 (s, 3H), 3.84-3.76 (m, 1H), 3.40 (s, 2H), 1.56 (s, 6H); $^{13}C\{^1H\}$ NMR ($CDCl_3$, 101 MHz): δ_C 190.0, 164.1, 162.8, 131.3, 126.9, 114.0, 106.2, 55.6, 36.1, 30.7, 27.6; HRMS (ESI-TOF, *m/z*): calcd for $C_{15}H_{17}O_6$ $[M + H]^+$, 293.1020; found, 293.1023; IR νmax (KBr, cm^{-1}): 2957, 2923, 1712, 1697, 1651, 1605, 1514, 1469, 1391, 1317, 1257, 1220, 1178, 1031, 847.

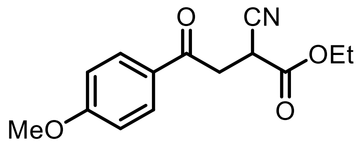

Ethyl 2-cyano-4-(4-methoxyphenyl)-4-oxobutanoate (3x)[8]

To a 10 mL Schlenk tube equipped with a magnetic stir bar was added ((1-(4-methoxyphenyl)vinyl)oxy)trimethylsilane **1a** (222 mg, 1 mmol), ethyl 2-cyanoacetate **2f** (57 mg, 0.5 mmol), Ag_2O (23 mg, 0.1 mmol), and 1,4-dioxane (2.0 mL). The reaction mixture was stirred at room temperature for about 6 h. The resulting mixture was concentrated and the residue was taken up in ethyl acetate. The organic layer was washed with brine, dried over Na_2SO_4 and concentrated. Purification of the crude product by column chromatography (silica gel; petroleum ether/ethyl acetate 10:1; R_f = 0.26) afforded **3x** in 91% yield (119 mg).

Colorless oil; ^1H NMR ($CDCl_3$, 400 MHz): δ_H 7.94 (d, *J* = 6.8 Hz, 2H), 6.95 (d, *J* = 6.8 Hz, 2H), 4.29 (q, *J* = 7.2 Hz, 2H), 4.14 (t, *J* = 5.6 Hz, 1H), 3.87 (s, 3H), 3.74–3.68 (m, 1H), 3.56–3.50 (m, 1H), 1.33 (t, *J* = 7.2 Hz, 3H); $^{13}C\{^1H\}$ NMR ($CDCl_3$, 101 MHz): δ_C 192.5, 165.4, 164.1, 130.4, 128.2, 116.4, 113.9, 63.0, 55.4, 37.5, 31.8, 13.8; HRMS (ESI-TOF, *m/z*): calcd for $C_{14}H_{16}NO_4$ $[M + H]^+$, 262.1074; found, 262.1076; IR ν max (film, cm^{-1}): 2960, 2938, 2251, 1724, 1712, 1678, 1603, 1574, 1511, 1350, 1266, 1231, 1171, 1038, 847.

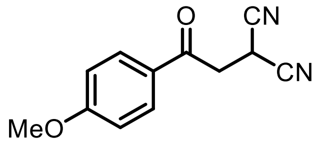

2-(2-(4-Methoxyphenyl)-2-oxoethyl)malononitrile (3y)[9]

To a 10 mL Schlenk tube equipped with a magnetic stir bar was added ((1-(4-methoxyphenyl)vinyl)oxy)trimethylsilane **1a** (222 mg, 1 mmol), malononitrile **2g** (33.3 mg, 0.5 mmol), Ag_2O (23 mg, 0.1 mmol), and 1,4-dioxane (2.0 mL). The reaction mixture was stirred at room temperature for about 6 h. The resulting mixture was concentrated and the residue was taken up in ethyl acetate. The organic layer was washed with brine, dried over Na_2SO_4 and concentrated. Purification of the crude product by column chromatography (silica gel; petroleum ether/ethyl acetate 10:1; R_f = 0.24) afforded **3y** in 93% yield (99 mg).

Colorless oil; ^{1}H NMR ($CDCl_3$, 400 MHz): δ_H 7.62 (d, J = 8.4 Hz, 2H), 6.99 (d, J = 6.8 Hz, 2H), 3.88 (s, 3H), 2.62 (s, 3H); $^{13}C\{^1H\}$ NMR ($CDCl_3$, 101 MHz): δ_C 174.0, 163.1, 129.8, 127.9, 114.4, 113.6, 113.4, 82.0, 55.6, 23.8; HRMS (ESI-TOF, *m/z*): calcd for $C_{12}H_{11}N_2O_2$ $[M + H]^+$, 215.0815; found, 215.0815; IR νmax (film, cm^{-1}): 2959, 2928, 2251, 1733, 1717, 1691, 1584, 1542, 1472, 1362, 1259, 1207, 1163, 1028, 859.

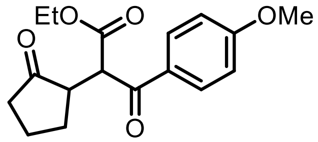

Ethyl 3-(4-methoxyphenyl)-3-oxo-2-(2-oxocyclopentyl)propanoate (3z)

To a 10 mL Schlenk tube equipped with a magnetic stir bar was added (cyclopent-1-en-1-yloxy)trimethylsilane **1t** (178 µL, 1 mmol), ethyl 3-(4-methoxyphenyl)-3-oxopropanoate **2b** (96 mg, 0.5 mmol), Ag_2O (23 mg, 0.1 mmol), and 1,4-dioxane (2.0 mL). The reaction mixture was stirred at room temperature for about 6 h. The resulting mixture was concentrated and the residue was taken up in ethyl acetate. The organic layer was washed with brine, dried over Na_2SO_4 and concentrated. Purification of the crude product by column chromatography (silica gel; petroleum ether/ethyl acetate 10:1; R_f = 0.25) afforded **3z** in 76% yield (105 mg).

Colorless oil; ^{1}H NMR ($CDCl_3$, 400 MHz): δ_H 7.98–7.94 (m, 2H), 6.97–6.92 (m, 2H), 4.80–4.59 (m, 1H), 4.20–4.12 (m, 2H), 3.88–3.87 (m, 3H), 3.01–2.80 (m, 1H), 2.40–2.24 (m, 2H), 2.21–2.07 (m, 2H), 2.00–1.75 (m, 2H), 1.20 (t, J = 8.4 Hz, 3H); $^{13}C\{^1H\}$ NMR ($CDCl_3$, 101 MHz): δ_C 220.7, 191.0, 167.7, 164.0, 130.9,

129.1, 127.7, 113.9, 61.4, 55.5, 45.8, 38.3, 23.2, 14.0; HRMS (ESI-TOF, *m/z*): calcd for $C_{17}H_{21}O_5$ [M + H]$^+$, 305.1384; found, 305.1389; IR νmax (film, cm^{-1}): 2974, 2938, 1747, 1736 1678, 1603, 1574, 1512, 1422, 1318, 1262, 1229, 1172, 1029, 834.

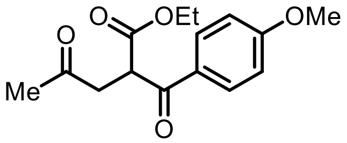

Ethyl 2-(4-methoxybenzoyl)-4-oxopentanoate (3aa)[10]

To a 10 mL Schlenk tube equipped with a magnetic stir bar was added trimethyl(prop-1-en-2-yloxy)silane **1u** (167 μL, 1 mmol), ethyl 3-(4-methoxyphenyl)-3-oxopropanoate **2b** (96 mg, 0.5 mmol), Ag_2O (23 mg, 0.1 mmol), and 1,4-dioxane (2.0 mL). The reaction mixture was stirred at room temperature for about 6 h. The resulting mixture was concentrated and the residue was taken up in ethyl acetate. The organic layer was washed with brine, dried over Na_2SO_4 and concentrated. Purification of the crude product by column chromatography (silica gel; petroleum ether/ethyl acetate 10:1; R_f = 0.27) afforded **3aa** in 85% yield (106 mg).

Colorless oil; ^{1}H NMR ($CDCl_3$, 400 MHz): δ_H 8.02 (d, *J* = 8.8 Hz, 2H), 6.95 (d, *J* = 6.8 Hz, 2H), 4.86 (t, *J* = 6.8 Hz, 1H), 4.13 (q, *J* = 6.8 Hz, 2H), 3.88 (s, 3H), 3.22–3.10 (m, 2H), 2.23 (s, 3H), 1.17 (t, *J* = 7.2 Hz, 3H); ^{13}C{^{1}H} NMR ($CDCl_3$, 101 MHz): δ_C 199.5, 190.0, 177.0, 164.3, 130.2, 127.1, 114.2, 103.6, 69.6, 55.6, 36.7, 28.7, 21.1; HRMS (ESI-TOF, *m/z*): calcd for $C_{15}H_{19}O_5$ [M + H]$^+$, 279.1227; found, 279.1225; IR ν max (film, cm^{-1}): 2982, 2943, 1743, 1719, 1676, 1601, 1575, 1512, 1421, 1366, 1261, 1170, 1027, 847.

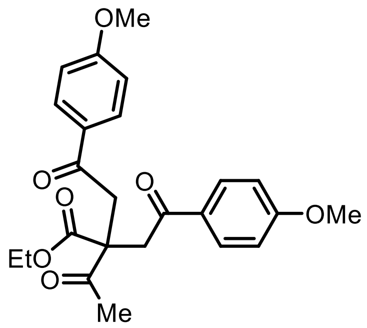

Ethyl 2-acetyl-4-(4-methoxyphenyl)-2-(2-(4-methoxyphenyl)-2-oxoethyl)-4-oxobutanoate (4)[11]

To a 10 mL Schlenk tube equipped with a magnetic stir bar was added ethyl 2-acetyl-4-(4-methoxyphenyl)-4-oxobutanoate **3a** (28 mg, 0.1 mmol), 1-methoxy-4-vinylbenzene (27 mg, 0.2 mmol), CuCl (10 mg, 0.01 mmol), and MeCN (5.0 mL). The reaction mixture was stirred at 60 °C in an oil bath for about 12 h. Then, the reaction mixture was cooled down to rt. The resulting mixture was concentrated and the residue was taken up in ethyl acetate. The organic layer was washed with brine, dried over Na_2SO_4 and concentrated. Purification of the crude product by column chromatography (silica gel; petroleum ether/ethyl acetate 4:1; R_f = 0.17) afforded **4** in 81% yield (34 mg).

Colorless oil; ^{1}H NMR ($CDCl_3$, 400 MHz): δ_H 7.93 (d, J = 7.2 Hz, 4H), 6.89 (d, J = 6.8 Hz, 4H), 4.19 (q, J = 7.2 Hz, 2H), 4.05–4.00 (m, 2H), 3.95–3.91 (m, 2H), 3.851 (s, 3H), 3.850 (s, 3H), 2.34 (s, 3H), 1.18 (t, J = 7.2 Hz, 3H); ^{13}C{^{1}H} NMR ($CDCl_3$, 101 MHz): δ_C 203.3, 196.2, 170.6, 163.8, 130.4, 129.3, 113.7, 61.9, 59.5, 55.5, 41.9, 26.3, 13.8; HRMS (ESI-TOF, *m/z*): calcd for $C_{24}H_{27}O_7$ [M + H]$^+$, 427.1751; found, 427.1752; IR ν max (film, cm^{-1}): 2981, 2928, 1741, 1717, 1684, 1599, 1576, 1449, 1357, 1264, 1172, 1023, 832.

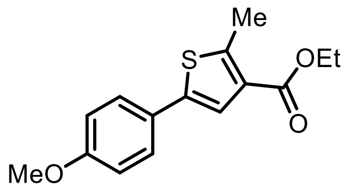

Ethyl 5-(4-methoxyphenyl)-2-methylthiophene-3-carboxylate (5)[12]

To a 10 mL Schlenk tube equipped with a magnetic stir bar was added ethyl 2-acetyl-4-(4-methoxyphenyl)-4-oxobutanoate **3a** (28 mg, 0.1 mmol), P_2S_5 (36 mg, 0.16 mmol), and DCE (5.0 mL). The reaction mixture was refluxed in an oil bath for 4 h. Then, the reaction mixture was cooled down to room temperature. The resulting mixture was concentrated and the residue was taken up in ethyl acetate. The organic layer was washed with brine, dried over Na_2SO_4 and concentrated. Purification

of the crude product by column chromatography (silica gel; petroleum ether/ethyl acetate 10:1; R_f = 0.31) afforded **5** in 94% yield (26 mg).

Colorless oil; ^{1}H NMR (CDCl$_3$, 400 MHz): δ_H 7.57 (d, J = 6.8 Hz, 2H), 6.91 (d, J = 6.8 Hz, 2H), 6.74 (s, 1H), 4.31 (q, J = 8.8 Hz, 2H), 3.83 (s, 3H), 2.63 (s, 3H), 1.37 (t, J = 7.2 Hz, 3H); ^{13}C{^{1}H} NMR (CDCl$_3$, 101 MHz): δ_C 164.2, 159.2, 158.0, 151.7, 125.1, 123.1, 115.2, 114.1, 103.8, 60.1, 55.3, 14.4, 13.8; HRMS (ESI-TOF, *m/z*): calcd for $C_{15}H_{17}O_3S$ [M + H]$^+$, 277.0893; found, 277.0898; IR νmax (film, cm^{-1}): 2986, 2931, 1705, 1606, 1516, 1478, 1318, 1248, 1176, 1031, 830, 780.

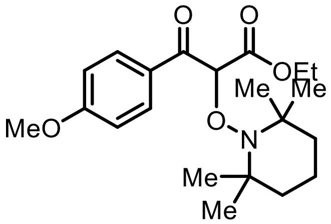

Ethyl 3-(4-methoxyphenyl)-3-oxo-2-((2,2,6,6-tetramethylpiperidin-1-yl)oxy)propanoate (6)

To a 10 mL Schlenk tube equipped with a magnetic stir bar was added ((1-(4-methoxyphenyl)vinyl)oxy)trimethylsilane **1a** (222 mg, 1 mmol), ethyl 3-(4-methoxyphenyl)-3-oxopropanoate **2b** (96 mg, 0.5 mmol), TEMPO (312.5 mg, 2 mmol), Ag_2O (23 mg, 0.1 mmol), and 1,4-dioxane (2.0 mL). The reaction mixture was stirred at room temperature for about 6 h. The resulting mixture was concentrated and the residue was taken up in ethyl acetate. The organic layer was washed with brine, dried over Na_2SO_4 and concentrated. Purification of the crude product by column chromatography (silica gel; petroleum ether/ethyl acetate 8:1; R_f = 0.18) afforded **6** in 67% yield (126 mg).

Colorless oil; ^{1}H NMR (CDCl$_3$, 400 MHz): δ_H 8.15 (d, *J* = 7.2 Hz, 2H), 6.95 (d, *J* = 7.2 Hz, 2H), 5.36 (s, 1H), 4.23-4.14 (m, 2H), 3.88 (s, 3H), 1.57-1.48 (m, 3H), 1.44-1.38 (m, 3H), 1.29 (s, 3H), 1.19-1.16 (m, 6H), 1.00 (s, 3H), 0.84 (s, 3H); ^{13}C{^{1}H} NMR (CDCl$_3$, 101 MHz): δ_C 192.1, 168.5, 163.9, 132.3, 127.4, 113.7, 93.1, 61.5, 60.3, 59.9, 55.4, 40.1, 39.9, 33.1, 32.4, 20.2, 16.9, 14.0; HRMS (ESI-TOF, *m/z*): calcd for $C_{21}H_{32}NO_5$ [M + H]$^+$, 378.2275; found, 378.2277; IR νmax (film, cm^{-1}): 2975, 2936, 1749, 1683, 1601, 1575, 1513, 1465, 1376, 1261, 1173, 1087, 1025, 851.

IV. NMR spectra

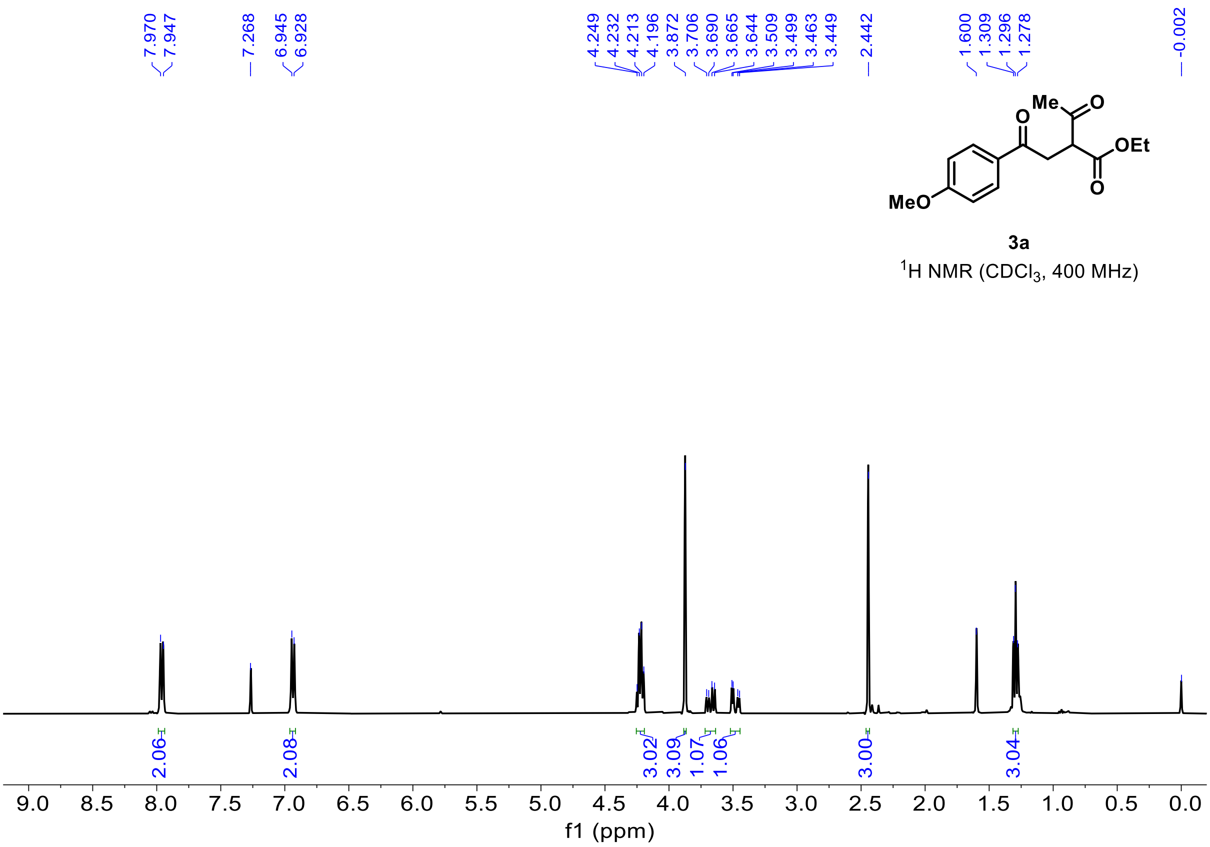

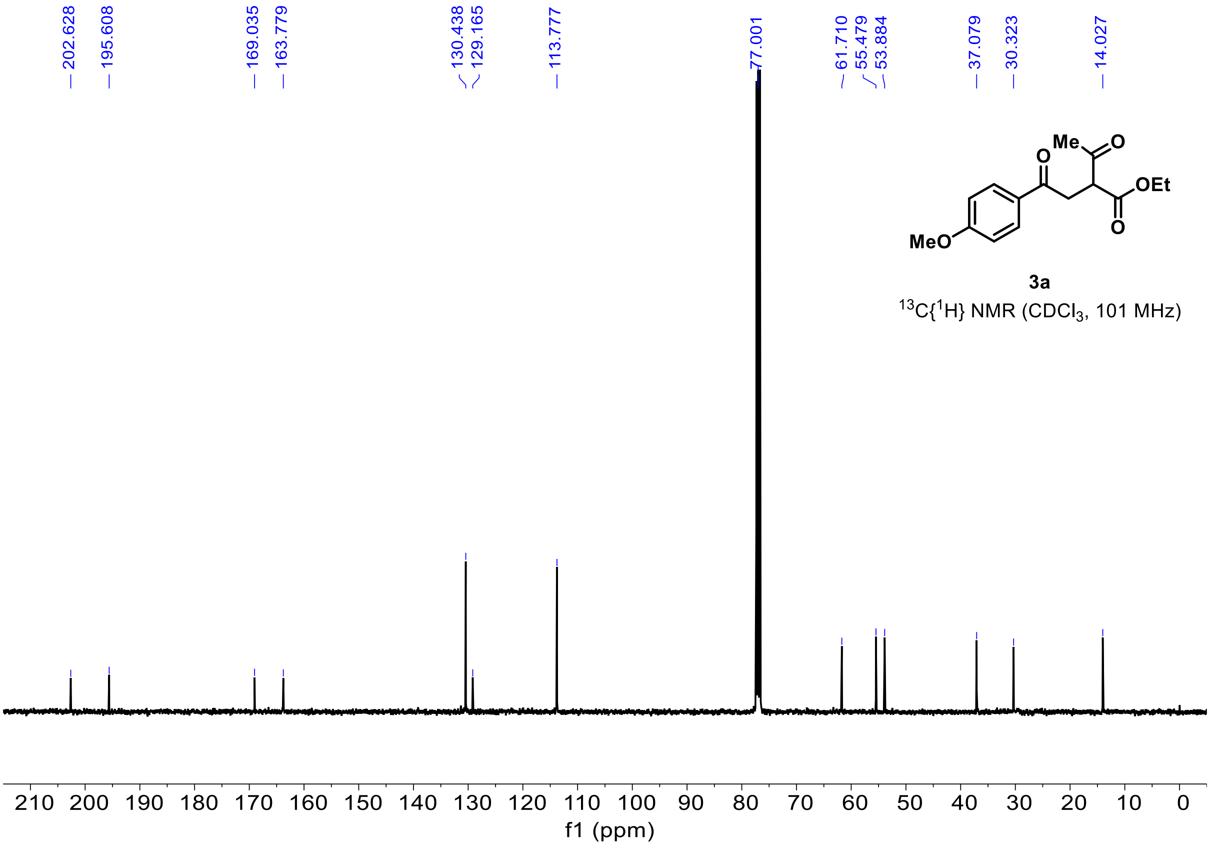

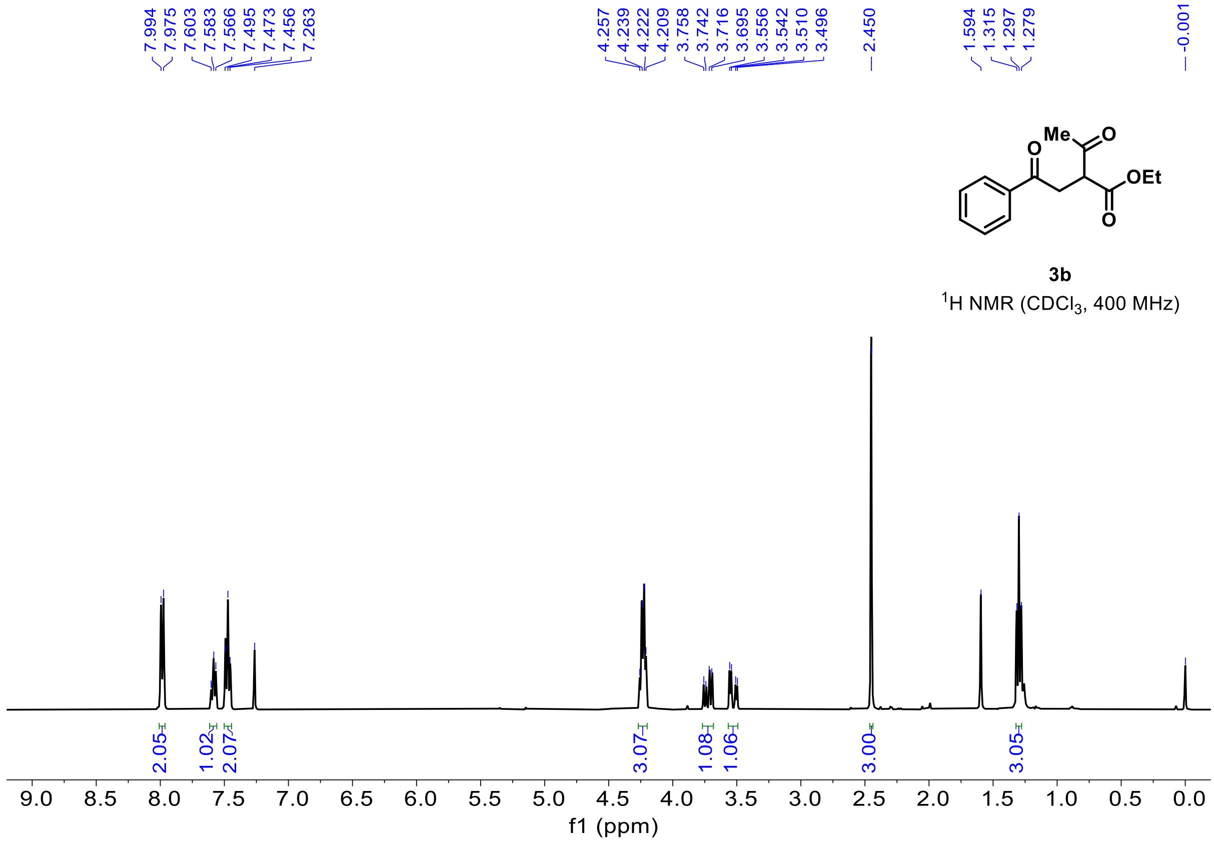

3b

^1H NMR ($CDCl_3$, 400 MHz)

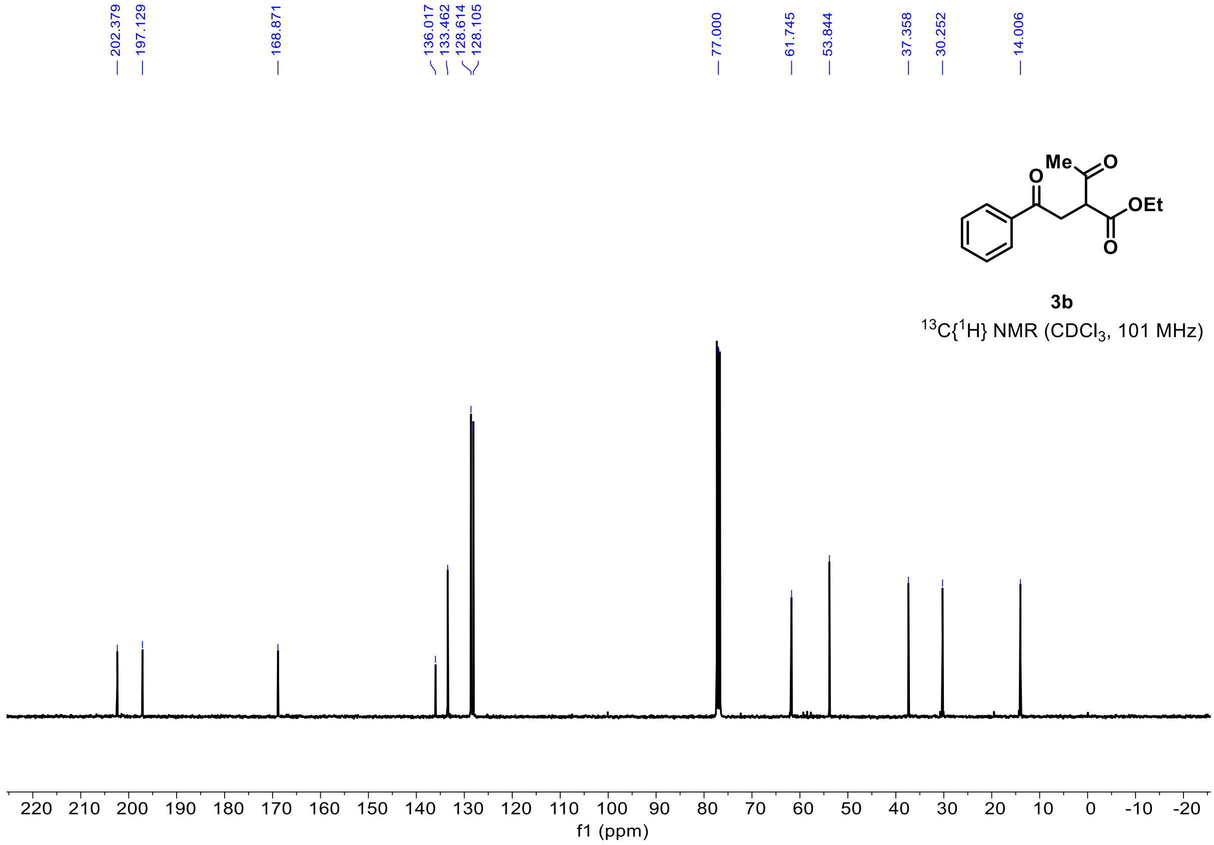

3b

$^{13}C\{^1H\}$ NMR ($CDCl_3$, 101 MHz)

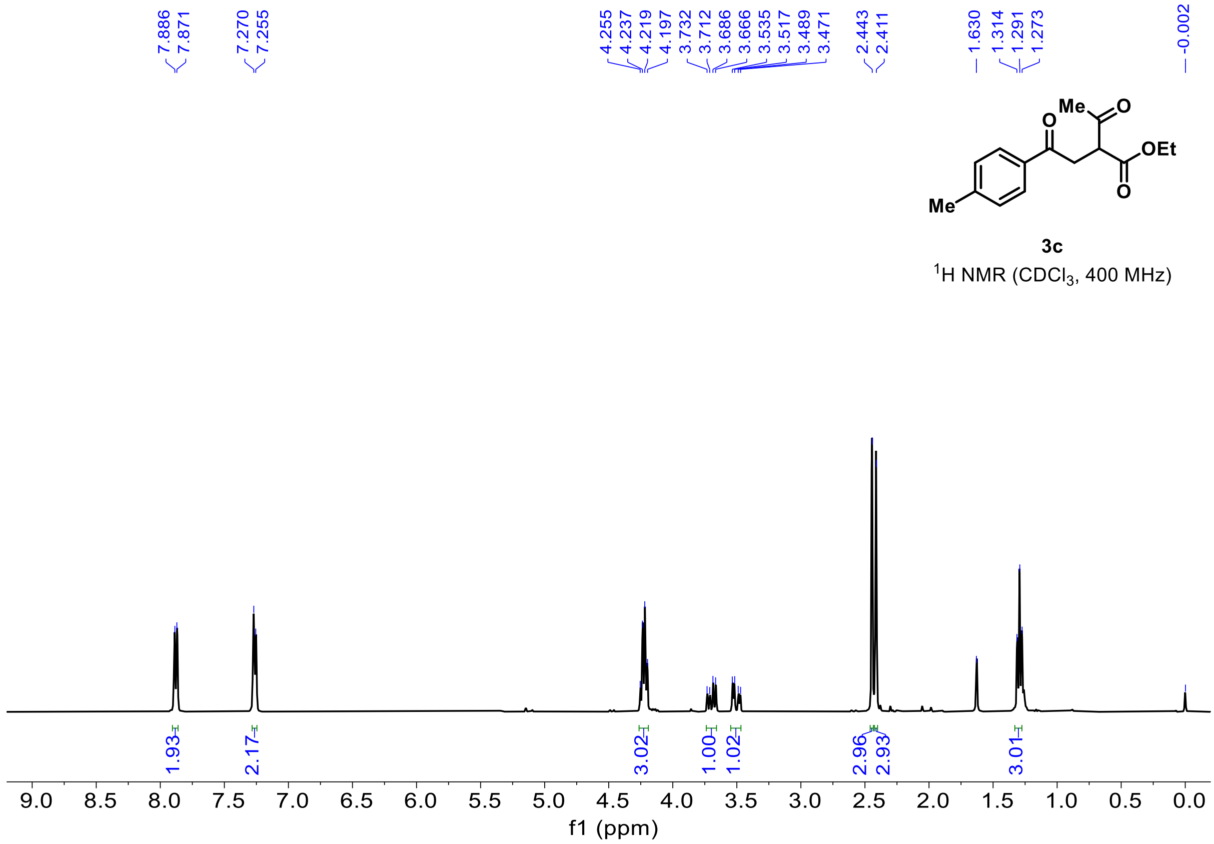

3c

^{1}H NMR ($CDCl_3$, 400 MHz)

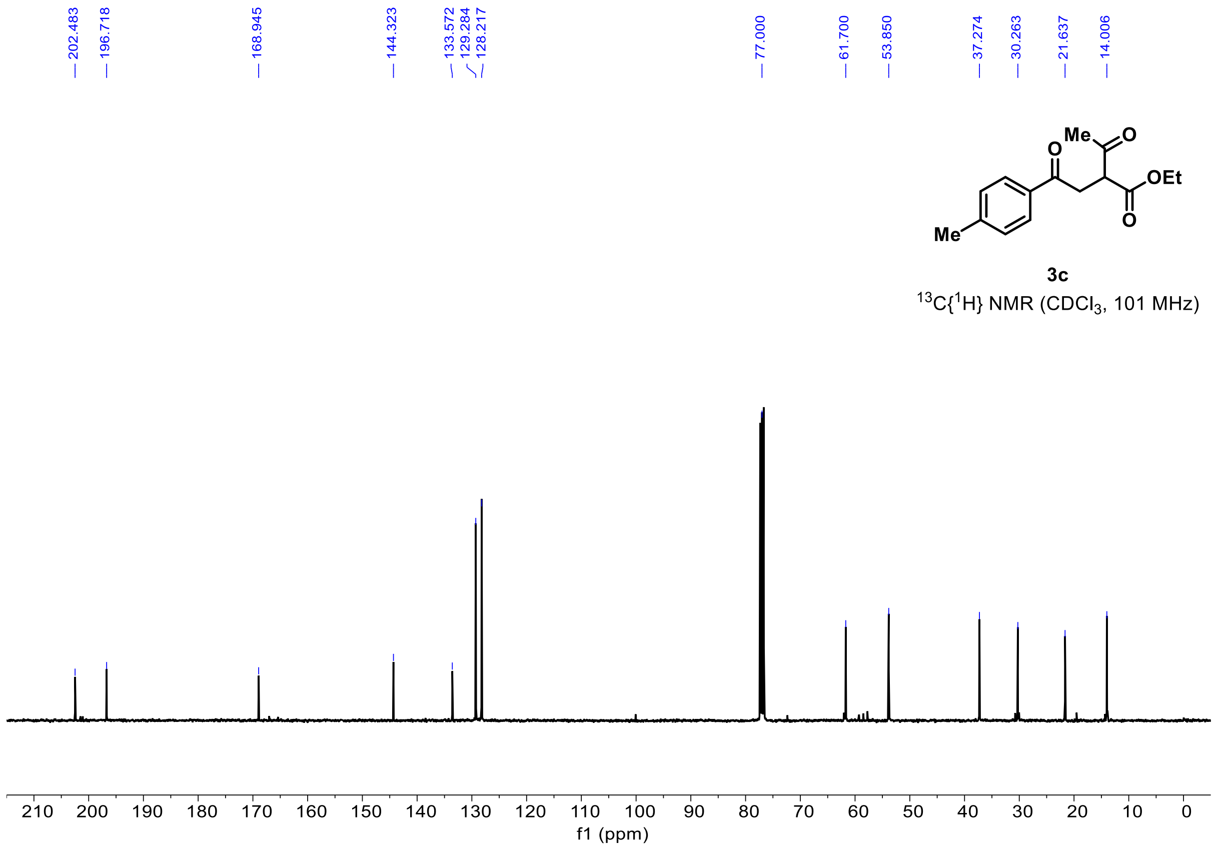

3c

$^{13}C\{^{1}H\}$ NMR ($CDCl_3$, 101 MHz)

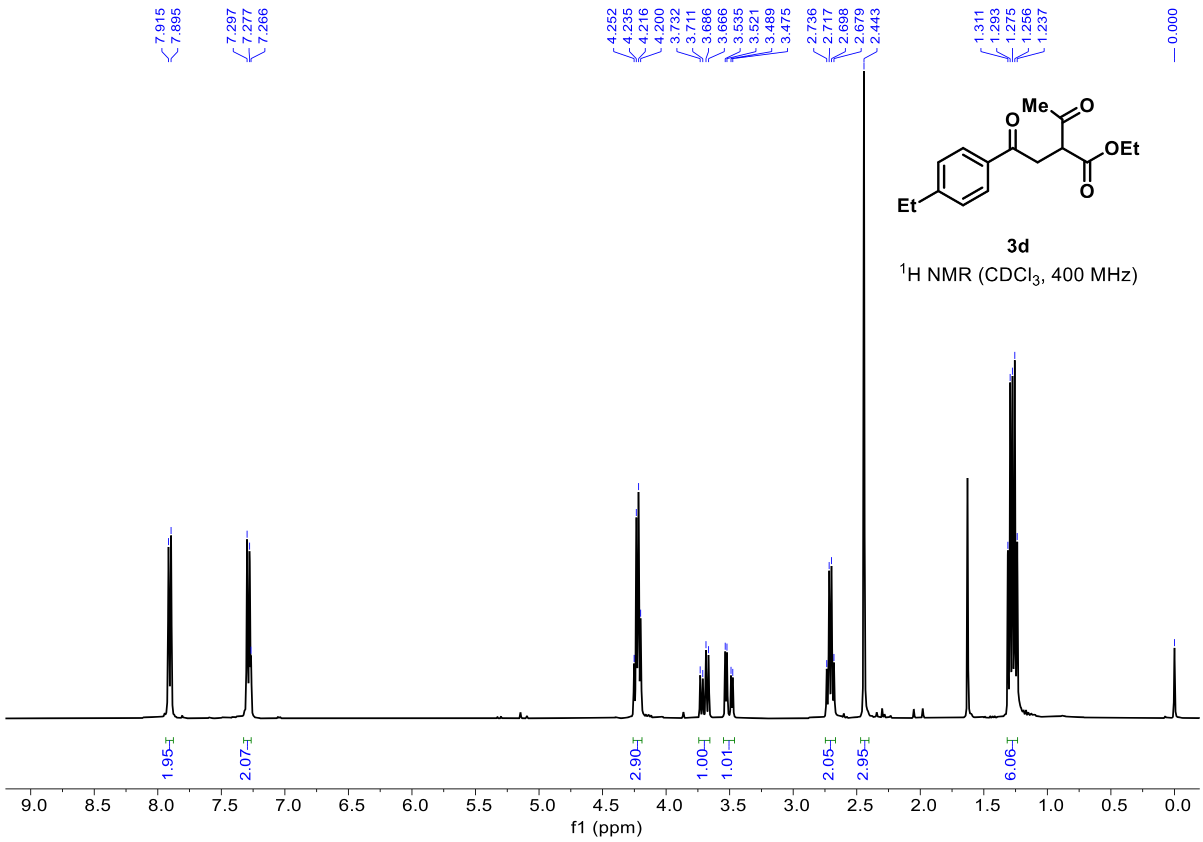
7.915
7.895
7.297
7.277
7.266
4.252
4.235
4.216
4.200
3.732
3.711
3.686
3.666
3.535
3.521
3.489
3.475
2.736
2.717
2.698
2.679
2.443
1.311
1.293
1.275
1.256
1.237
0.000
Me
O
O
OEt
O
Et
3d
1H NMR (CDCl3, 400 MHz)
1.95
2.07
2.90
1.00
1.01
2.05
2.95
6.06
9.0
8.5
8.0
7.5
7.0
6.5
6.0
5.5
5.0
4.5
4.0
3.5
3.0
2.5
2.0
1.5
1.0
0.5
0.0
f1 (ppm)

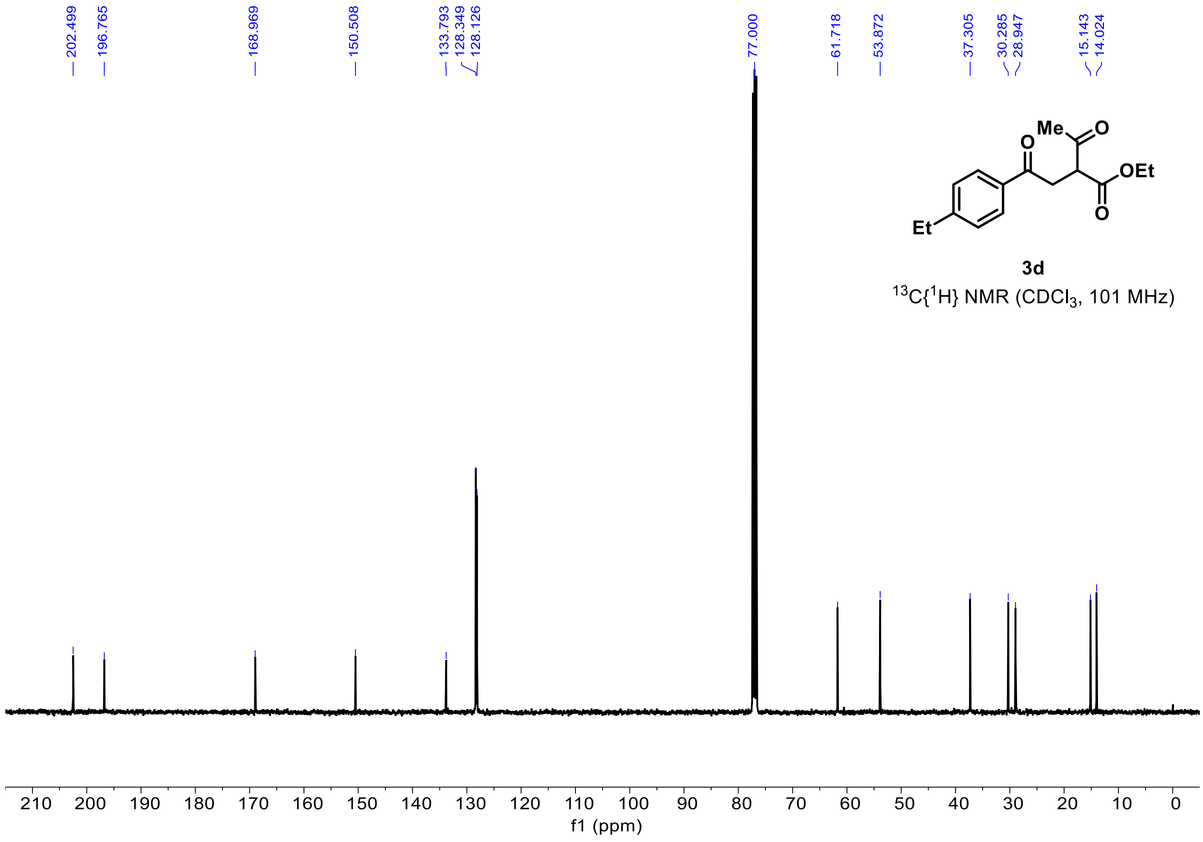
202.499
196.765
168.969
150.508
133.793
128.349
128.126
77.000
61.718
53.872
37.305
30.285
28.947
15.143
14.024
Me
O
O
OEt
O
Et
3d
13C{1H} NMR (CDCl3, 101 MHz)
210
200
190
180
170
160
150
140
130
120
110
100
90
80
70
60
50
40
30
20
10
0
f1 (ppm)

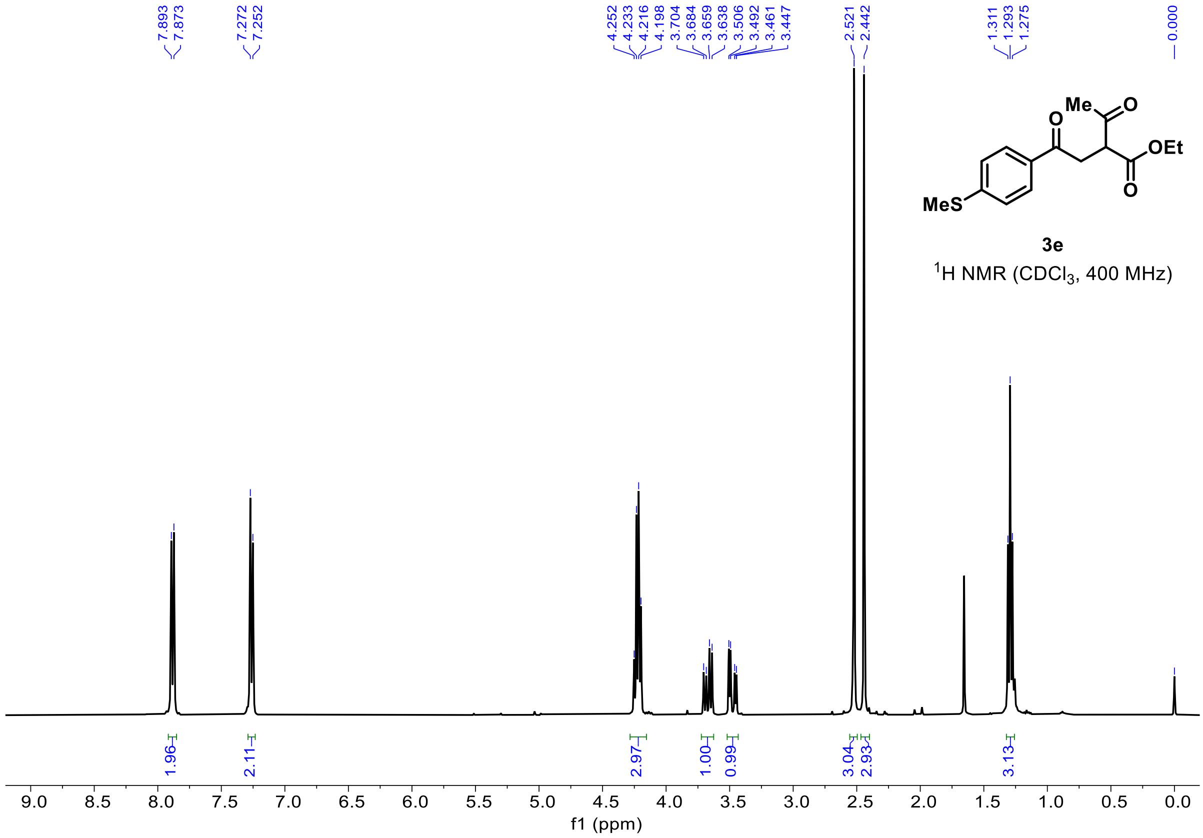
7.893
7.873
7.272
7.252
4.252
4.233
4.216
4.198
3.704
3.684
3.659
3.638
3.506
3.492
3.461
3.447
2.521
2.442
1.311
1.293
1.275
0.000
Me
O
O
OEt
O
MeS
3e
^{1}H NMR (CDCl$_3$, 400 MHz)
1.96
2.11
2.97
1.00
0.99
3.04
2.93
3.13
f1 (ppm)

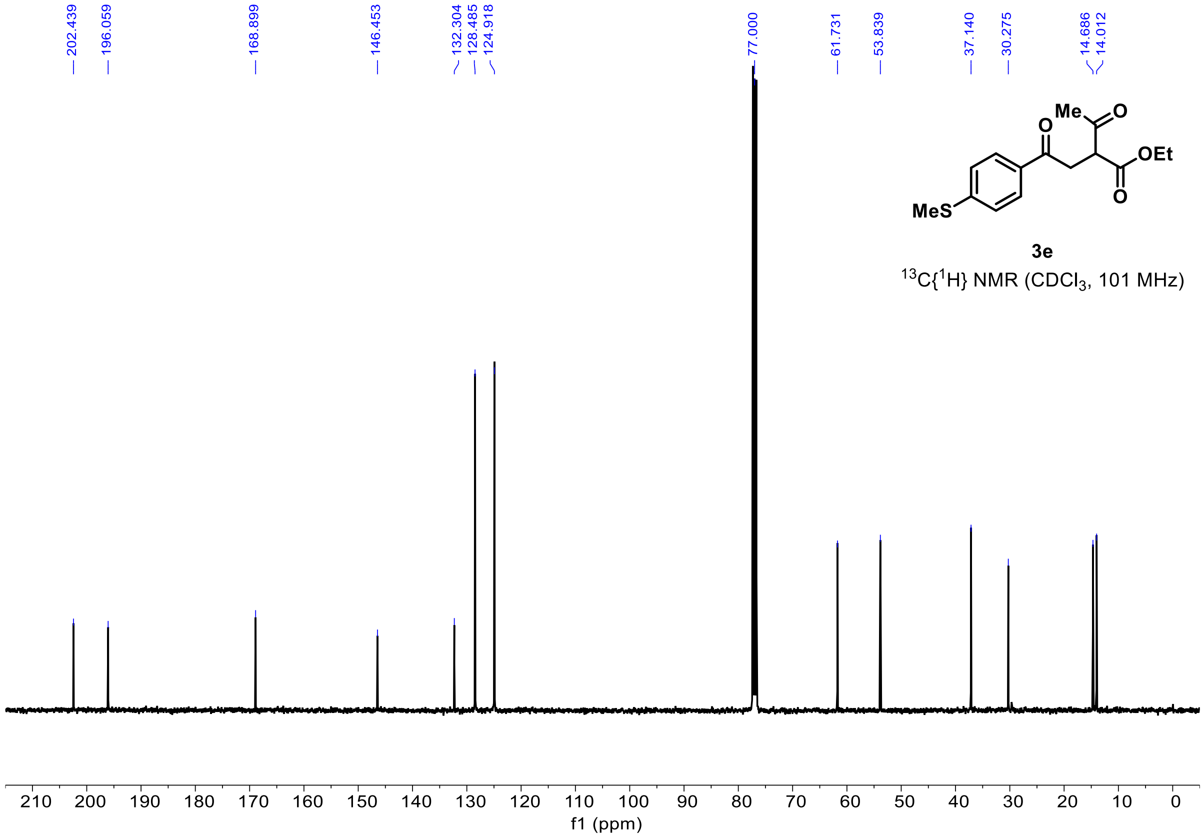
202.439
196.059
168.899
146.453
132.304
128.485
124.918
77.000
61.731
53.839
37.140
30.275
14.686
14.012
Me
O
O
OEt
O
MeS
3e
^{13}C{^{1}H} NMR (CDCl$_3$, 101 MHz)
f1 (ppm)

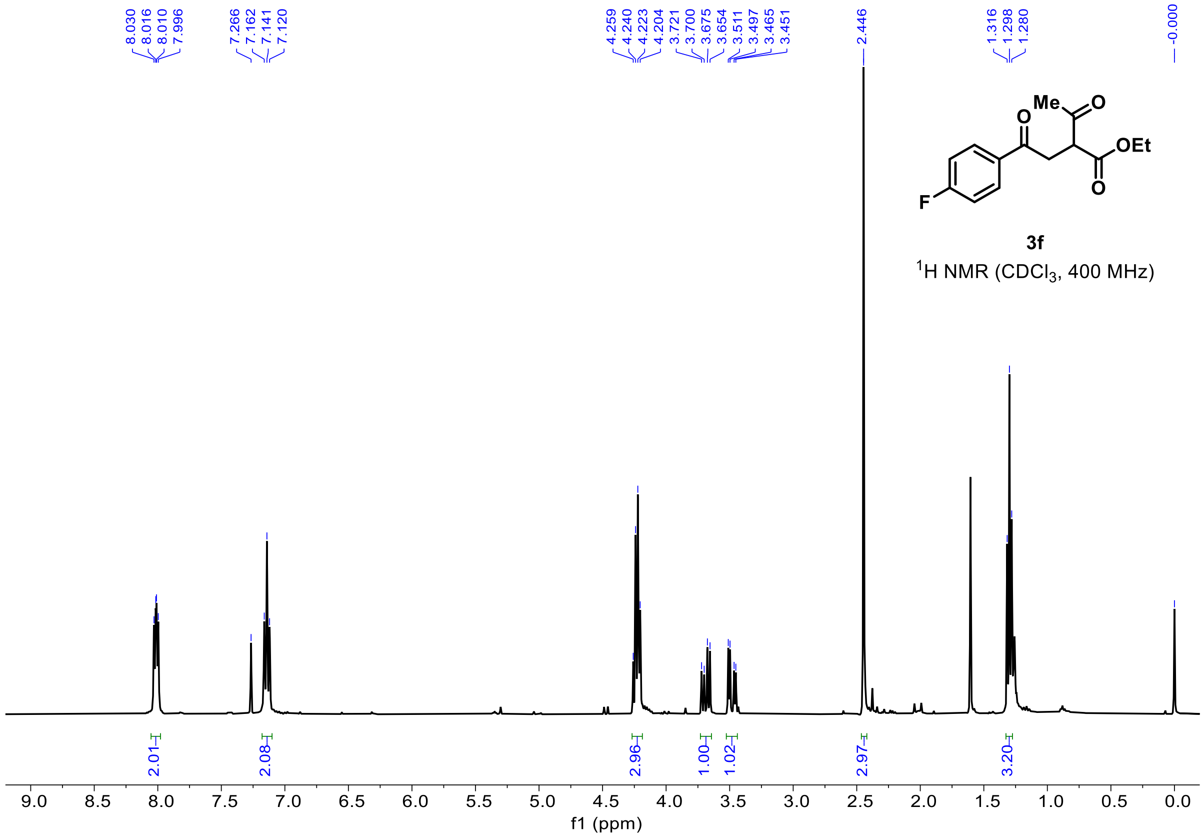
Me
O
O
OEt
O
F
3f
1H NMR (CDCl3, 400 MHz)
f1 (ppm)

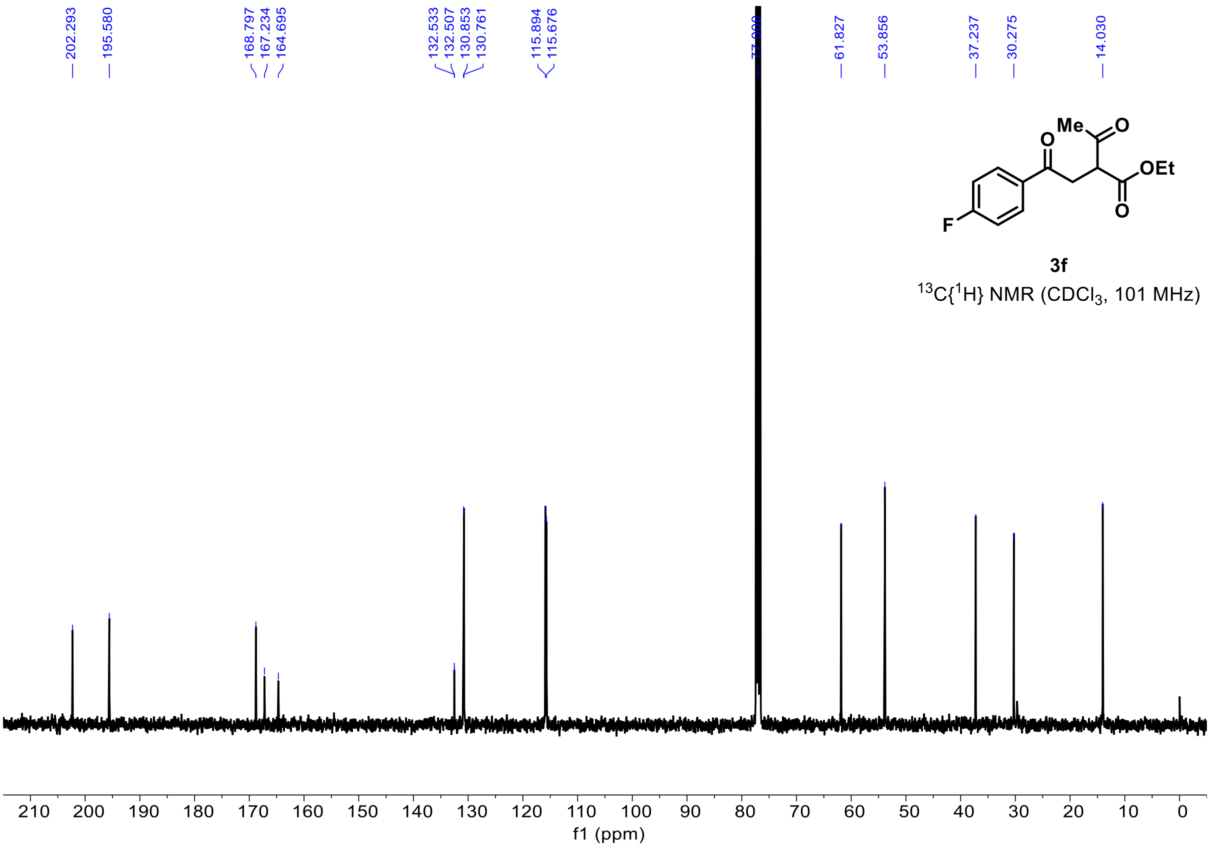
Me
O
O
OEt
O
F
3f
13C{1H} NMR (CDCl3, 101 MHz)
f1 (ppm)

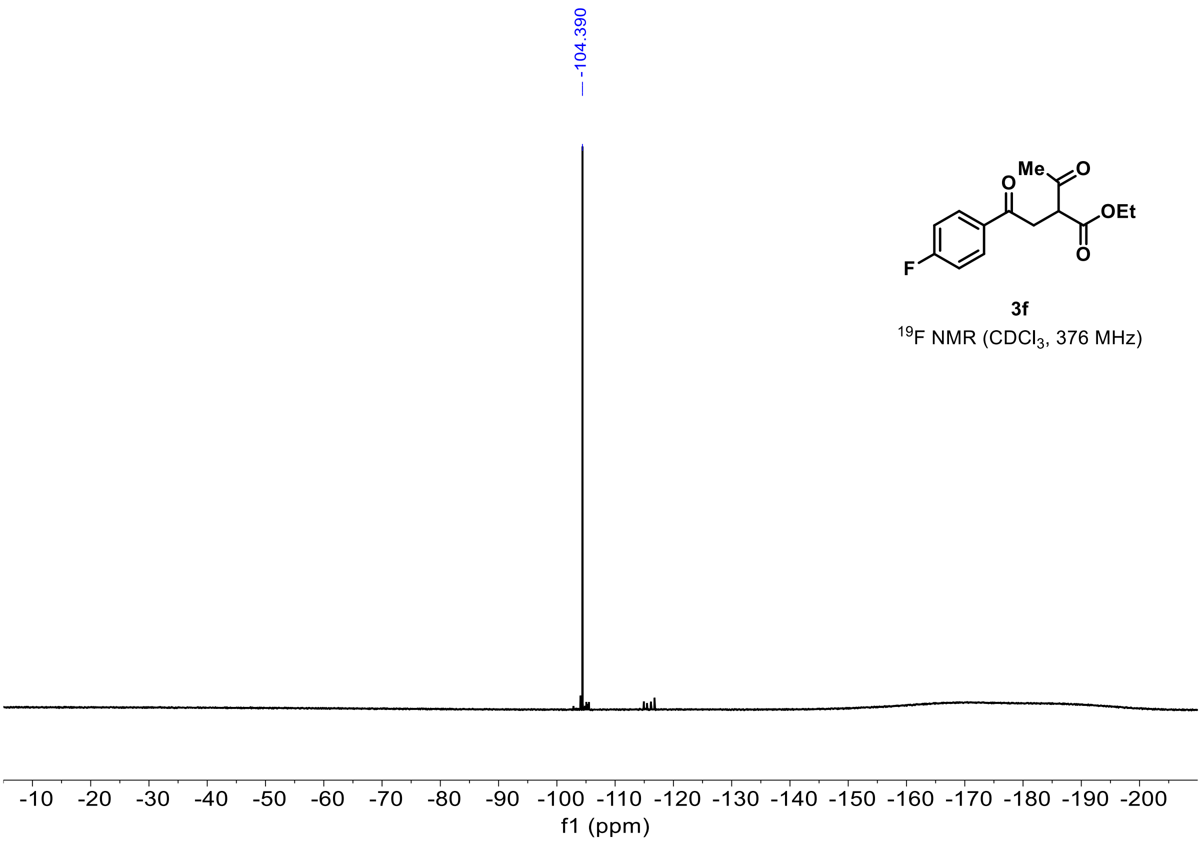
-104.390
Me
O
O
OEt
O
F
3f
^{19}F NMR (CDCl$_3$, 376 MHz)
-10 -20 -30 -40 -50 -60 -70 -80 -90 -100 -110 -120 -130 -140 -150 -160 -170 -180 -190 -200
f1 (ppm)

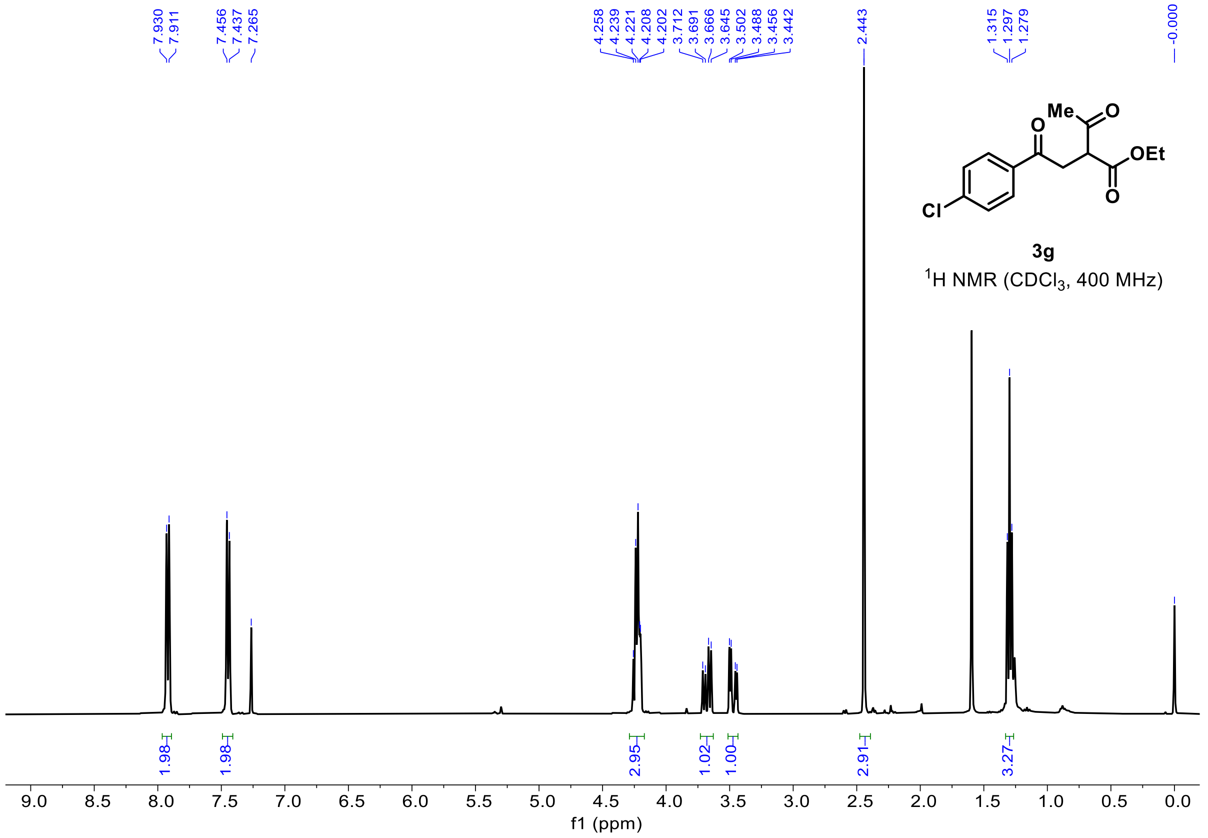

3g
^{1}H NMR ($CDCl_3$, 400 MHz)

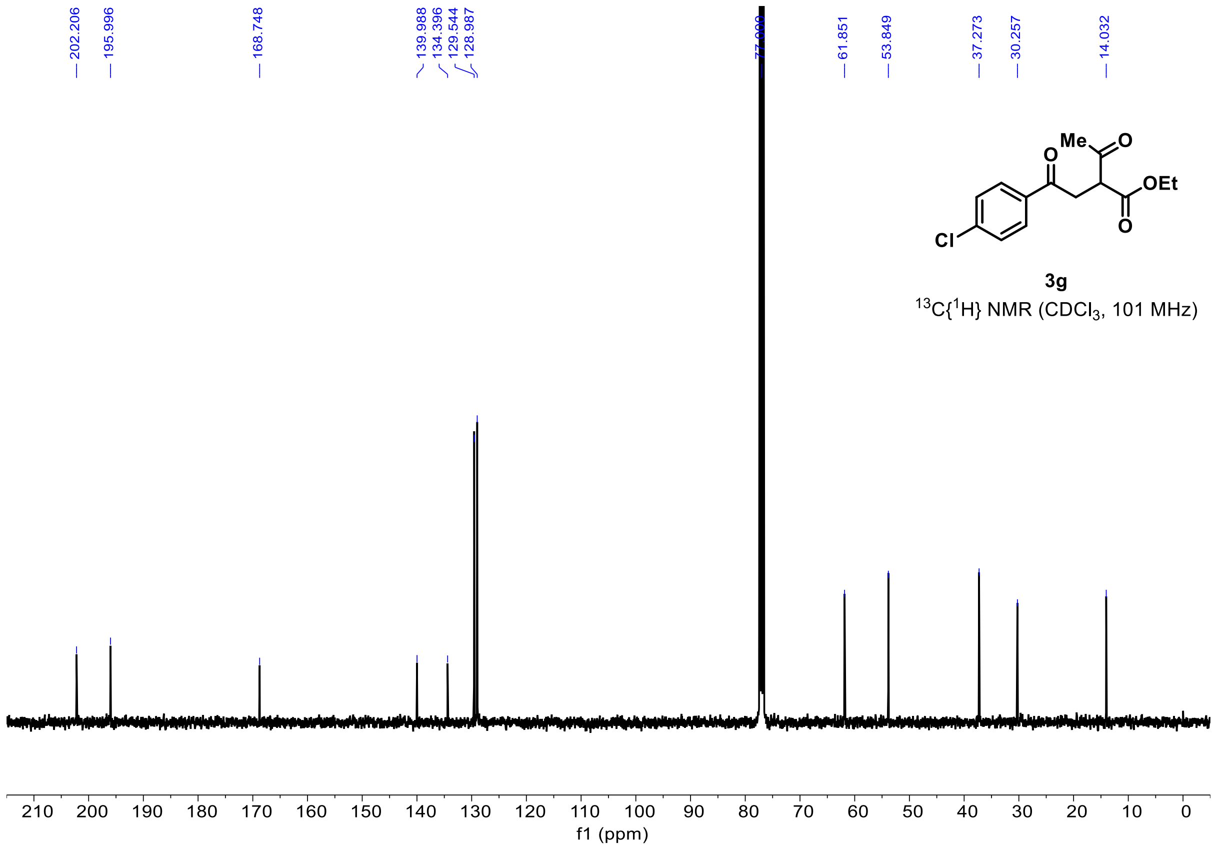

3g
$^{13}C\{^{1}H\}$ NMR ($CDCl_3$, 101 MHz)

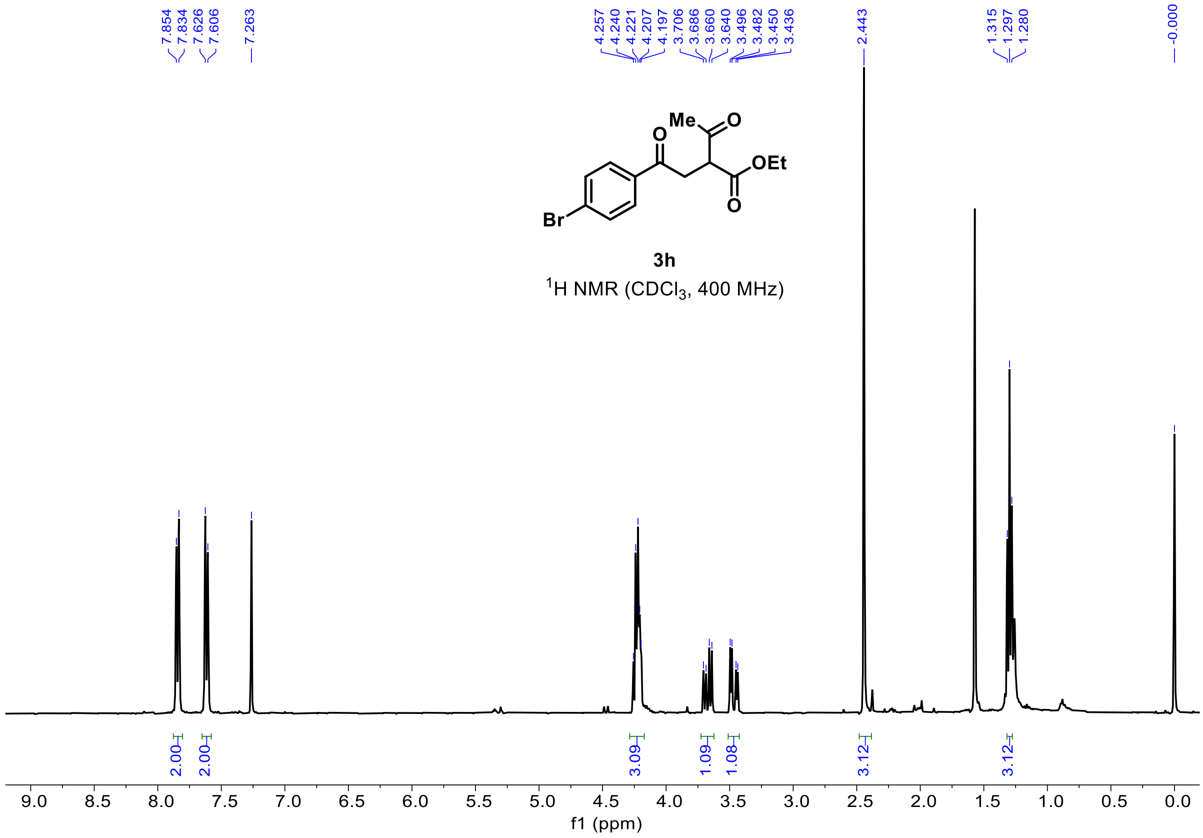
7.854
7.834
7.626
7.606
7.263
4.257
4.240
4.221
4.207
4.197
3.706
3.686
3.660
3.640
3.496
3.482
3.450
3.436
2.443
1.315
1.297
1.280
-0.000
Me
O
O
OEt
O
Br
3h
1H NMR (CDCl3, 400 MHz)
2.00
2.00
3.09
1.09
1.08
3.12
3.12
f1 (ppm)

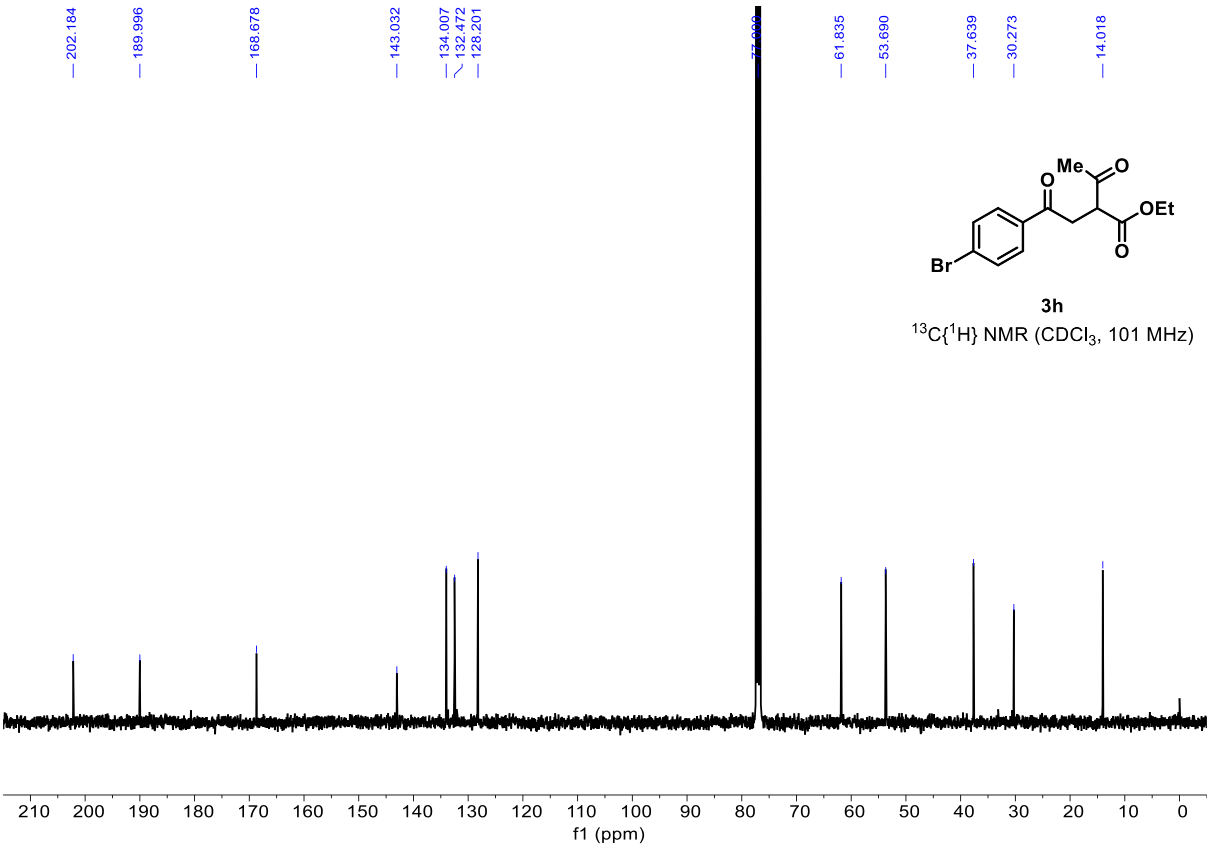
202.184
189.996
168.678
143.032
134.007
132.472
128.201
61.835
53.690
37.639
30.273
14.018
Me
O
O
OEt
O
Br
3h
13C{1H} NMR (CDCl3, 101 MHz)
f1 (ppm)

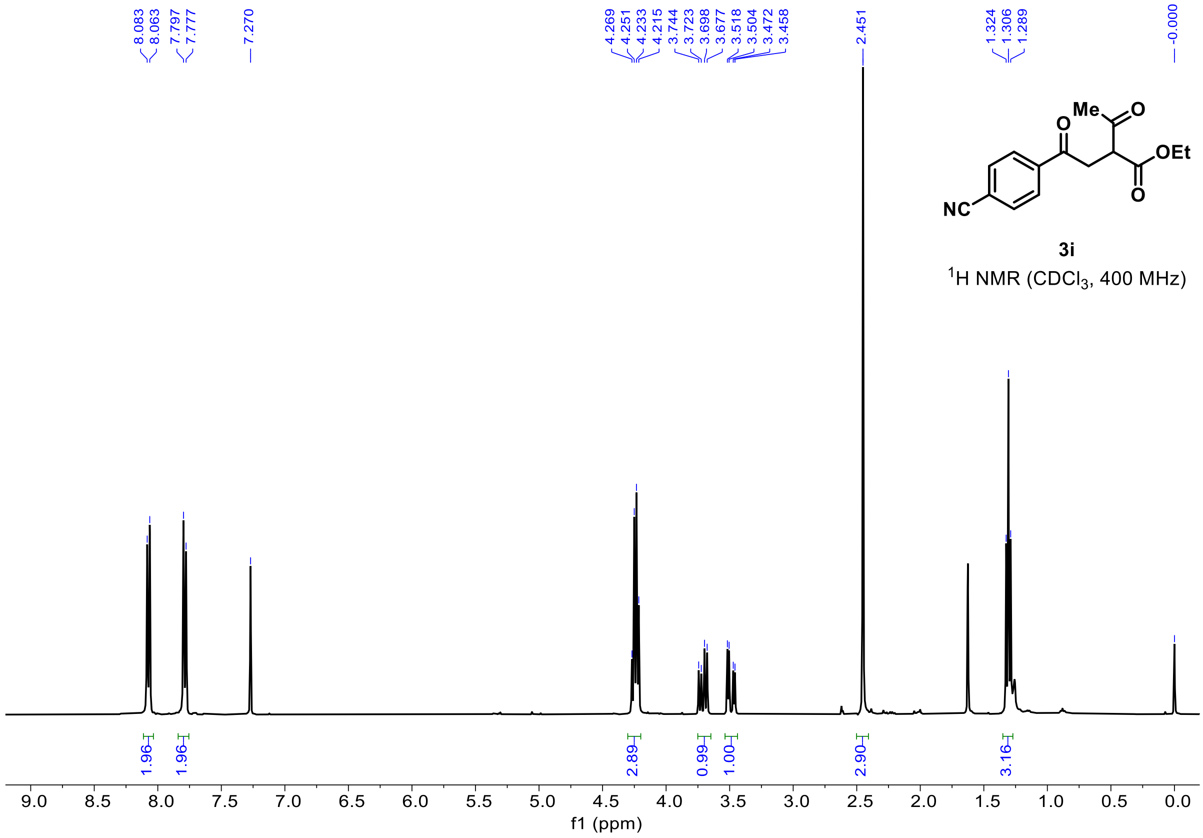

Me
O
O
OEt
O
NC
3i
^{1}H NMR (CDCl$_3$, 400 MHz)
f1 (ppm)

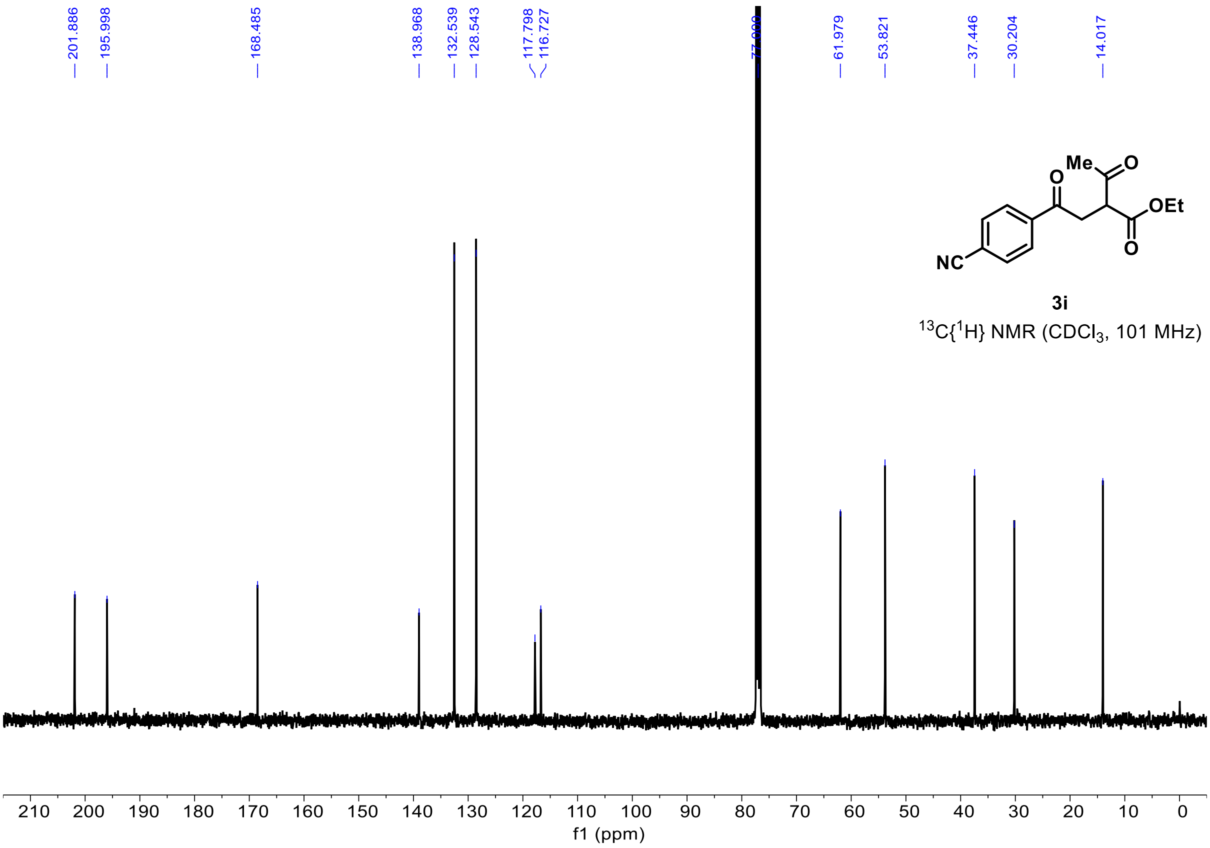

Me
O
O
OEt
O
NC
3i
^{13}C{^{1}H} NMR (CDCl$_3$, 101 MHz)
f1 (ppm)

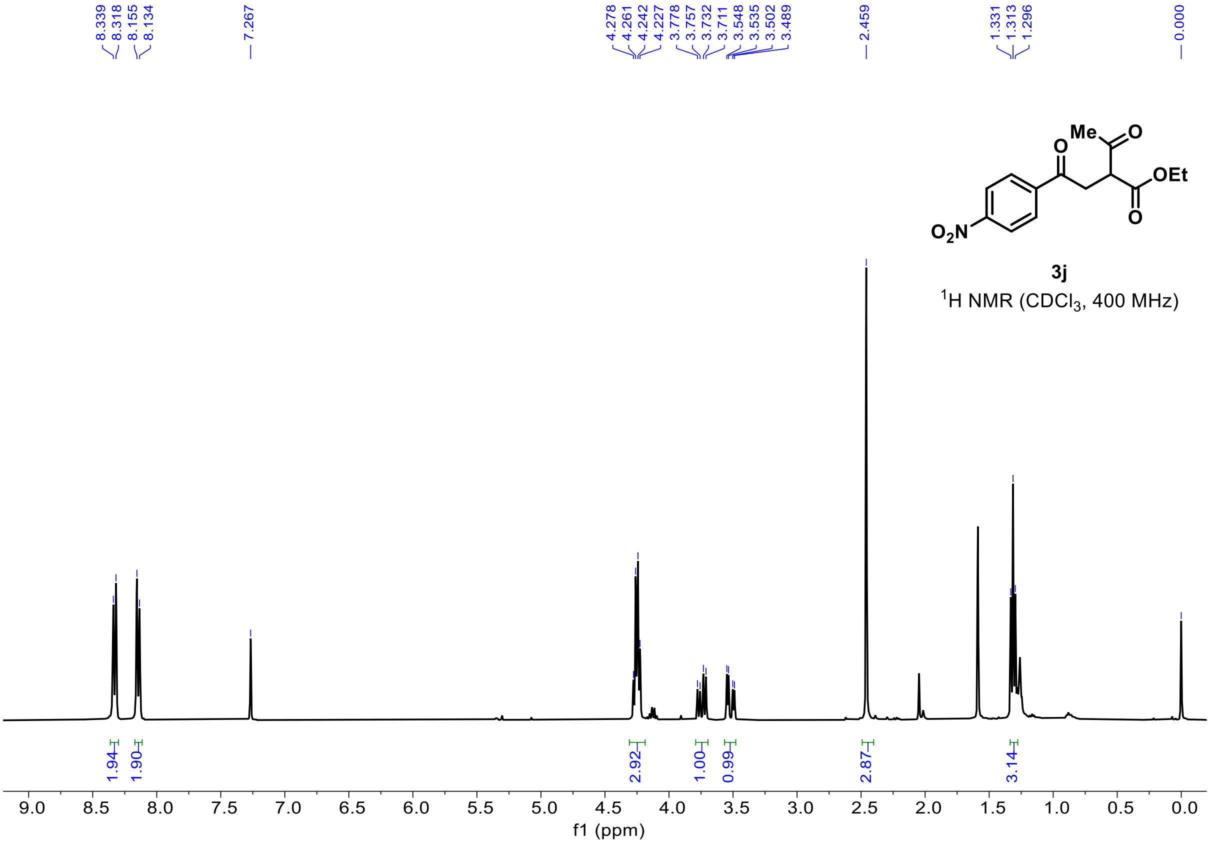
8.339
8.318
8.155
8.134
7.267
4.278
4.261
4.242
4.227
3.778
3.757
3.732
3.711
3.548
3.535
3.502
3.489
2.459
1.331
1.313
1.296
0.000
Me
O
O
OEt
O
O2N
3j
1H NMR (CDCl3, 400 MHz)
1.94
1.90
2.92
1.00
0.99
2.87
3.14
9.0 8.5 8.0 7.5 7.0 6.5 6.0 5.5 5.0 4.5 4.0 3.5 3.0 2.5 2.0 1.5 1.0 0.5 0.0
f1 (ppm)

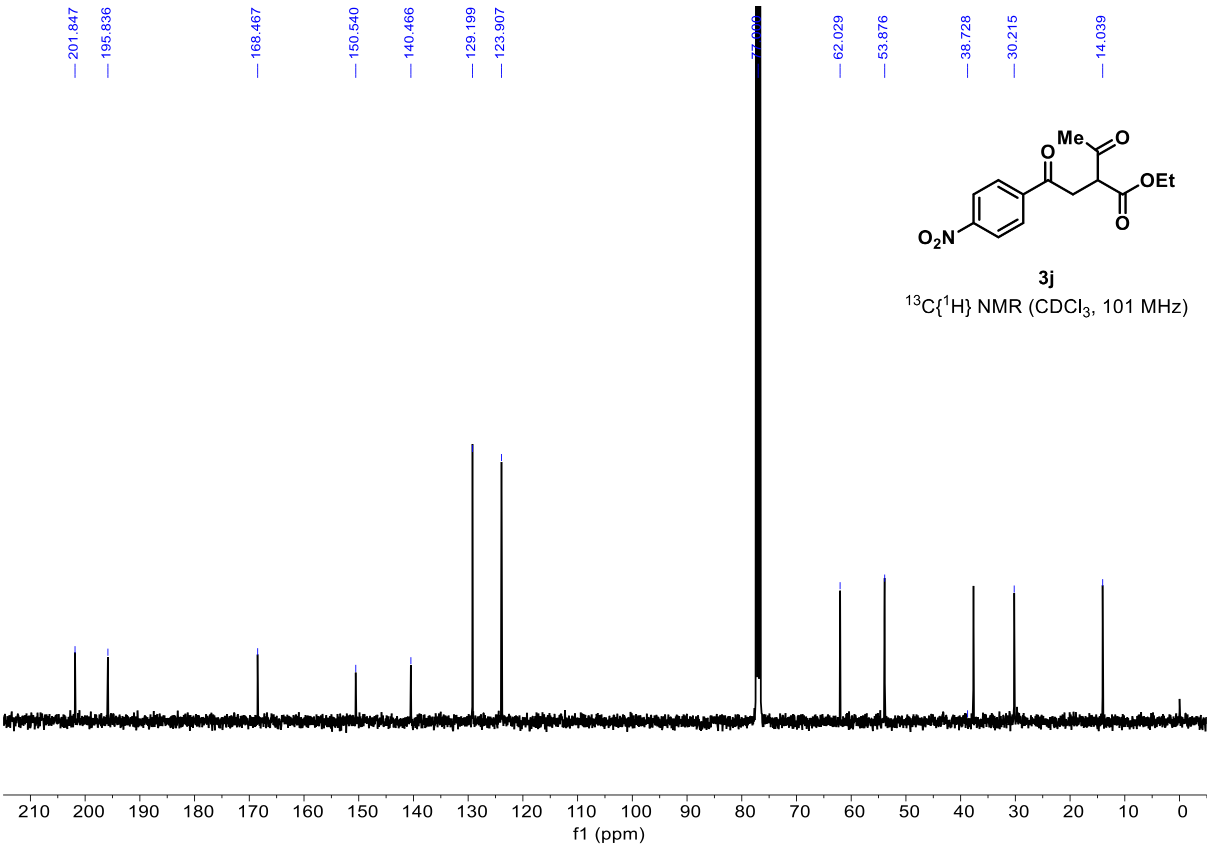
201.847
195.836
168.467
150.540
140.466
129.199
123.907
62.029
53.876
38.728
30.215
14.039
Me
O
O
OEt
O
O2N
3j
13C{1H} NMR (CDCl3, 101 MHz)
210 200 190 180 170 160 150 140 130 120 110 100 90 80 70 60 50 40 30 20 10 0
f1 (ppm)

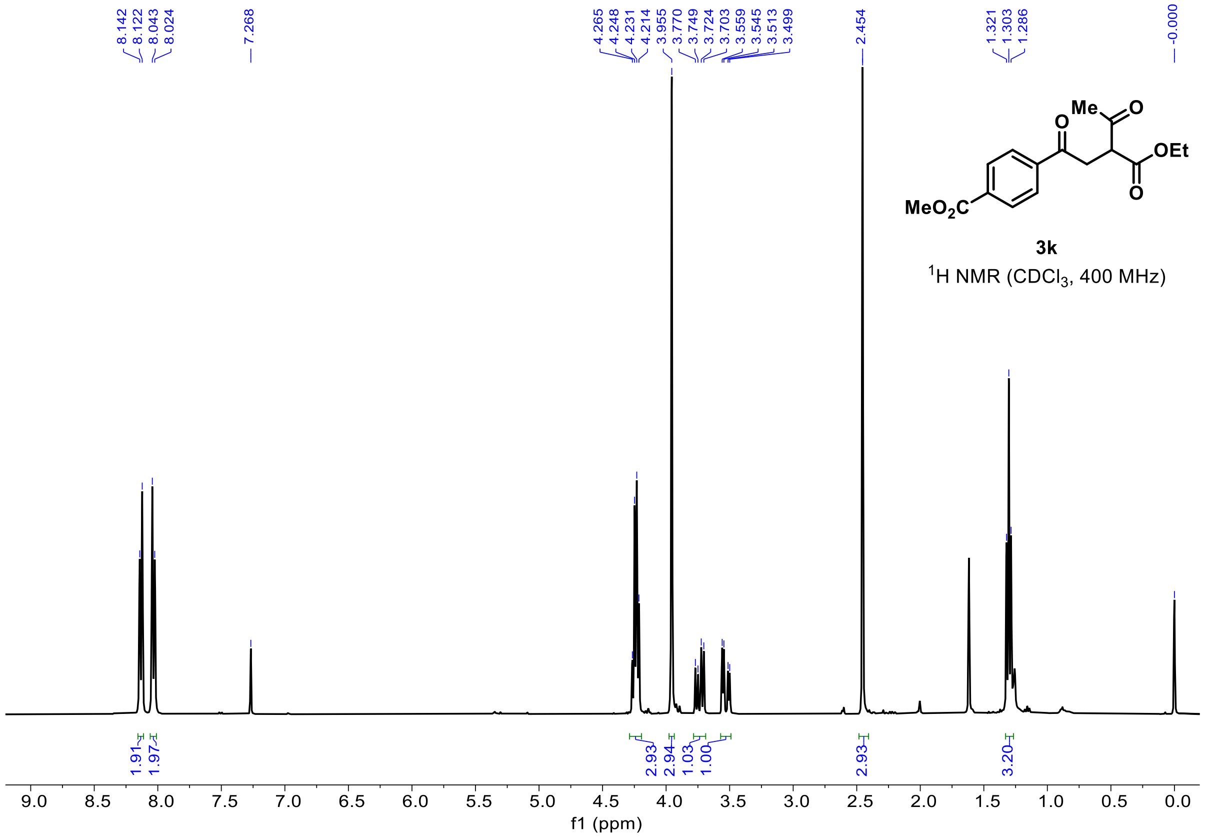
8.142
8.122
8.043
8.024
7.268
4.265
4.248
4.231
4.214
3.955
3.770
3.749
3.724
3.703
3.559
3.545
3.513
3.499
2.454
1.321
1.303
1.286
-0.000
Me
O
O
OEt
O
MeO2C
3k
1H NMR (CDCl3, 400 MHz)
1.91
1.97
2.93
2.94
1.03
1.00
2.93
3.20
9.0 8.5 8.0 7.5 7.0 6.5 6.0 5.5 5.0 4.5 4.0 3.5 3.0 2.5 2.0 1.5 1.0 0.5 0.0
f1 (ppm)

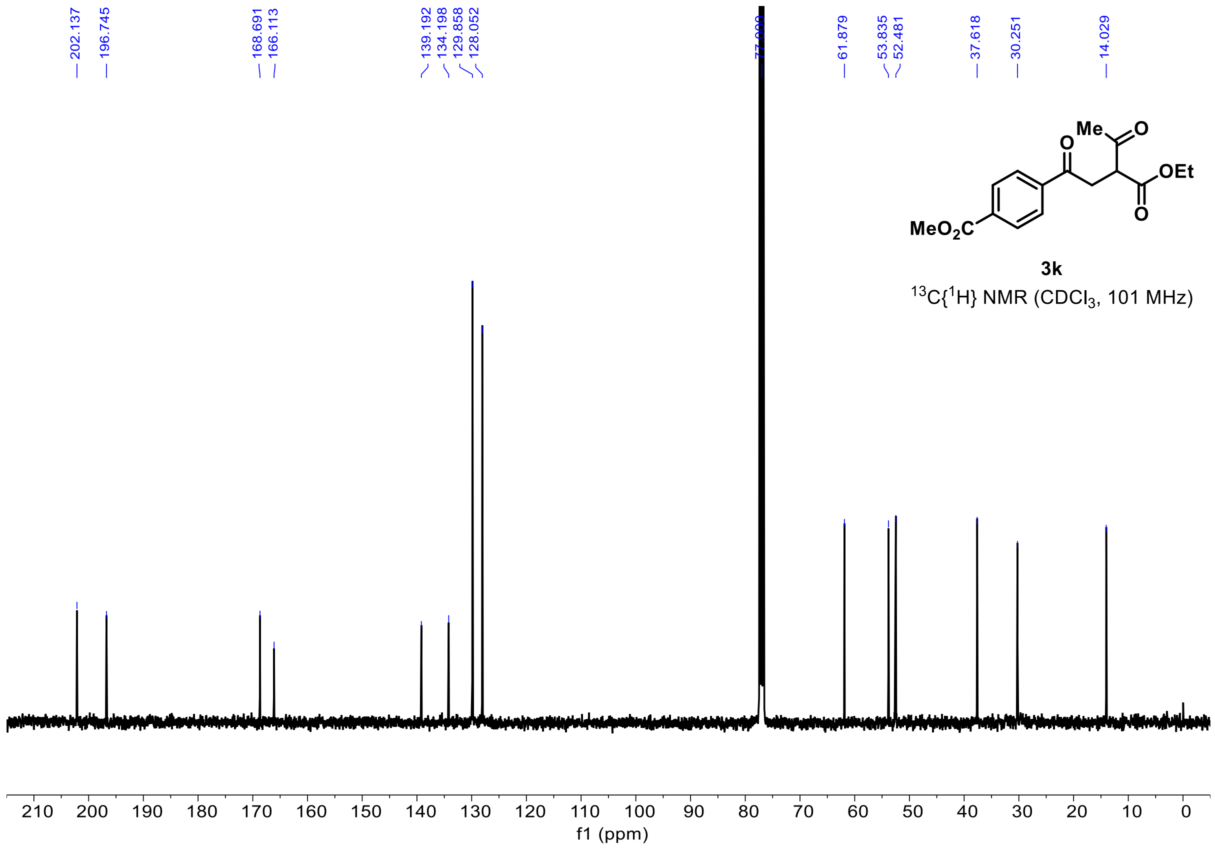
202.137
196.745
168.691
166.113
139.192
134.198
129.858
128.052
61.879
53.835
52.481
37.618
30.251
14.029
Me
O
O
OEt
O
MeO2C
3k
13C{1H} NMR (CDCl3, 101 MHz)
210 200 190 180 170 160 150 140 130 120 110 100 90 80 70 60 50 40 30 20 10 0
f1 (ppm)

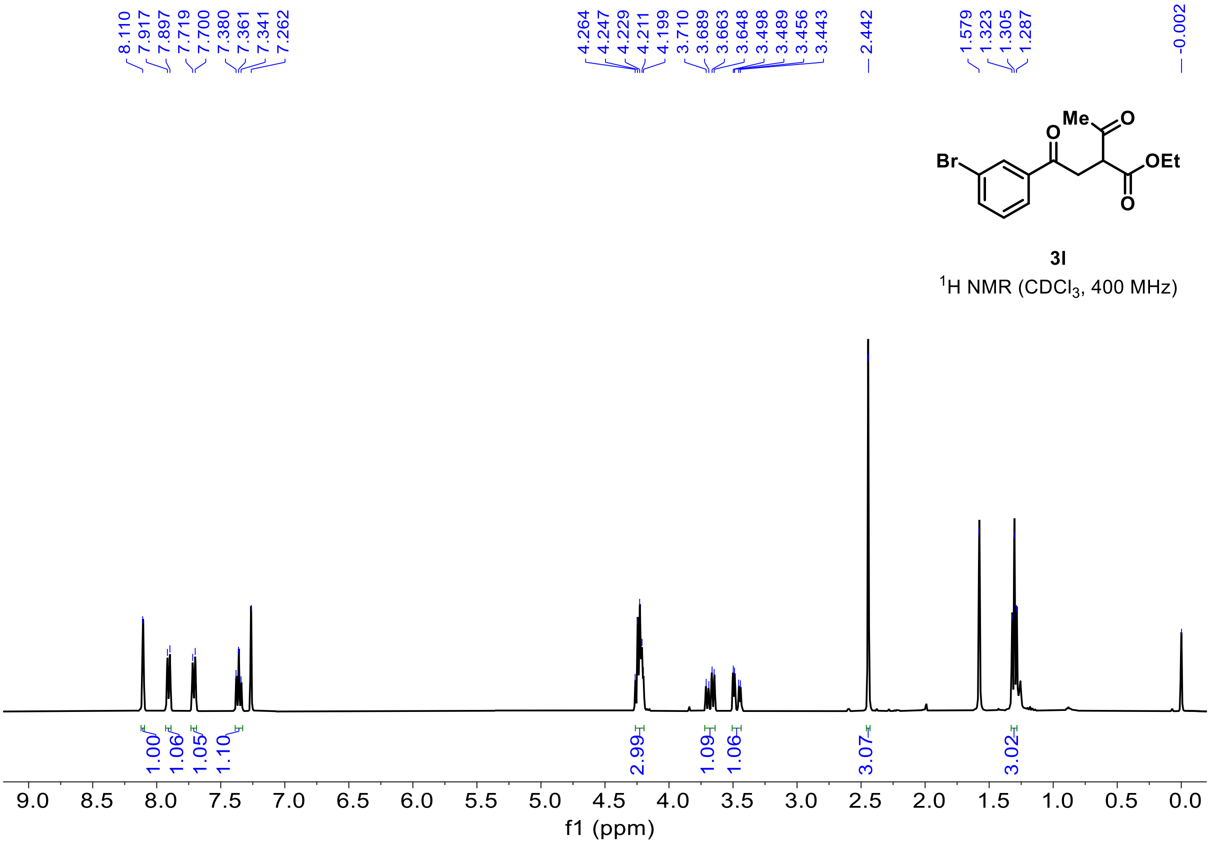

3l

^{1}H NMR ($CDCl_3$, 400 MHz)

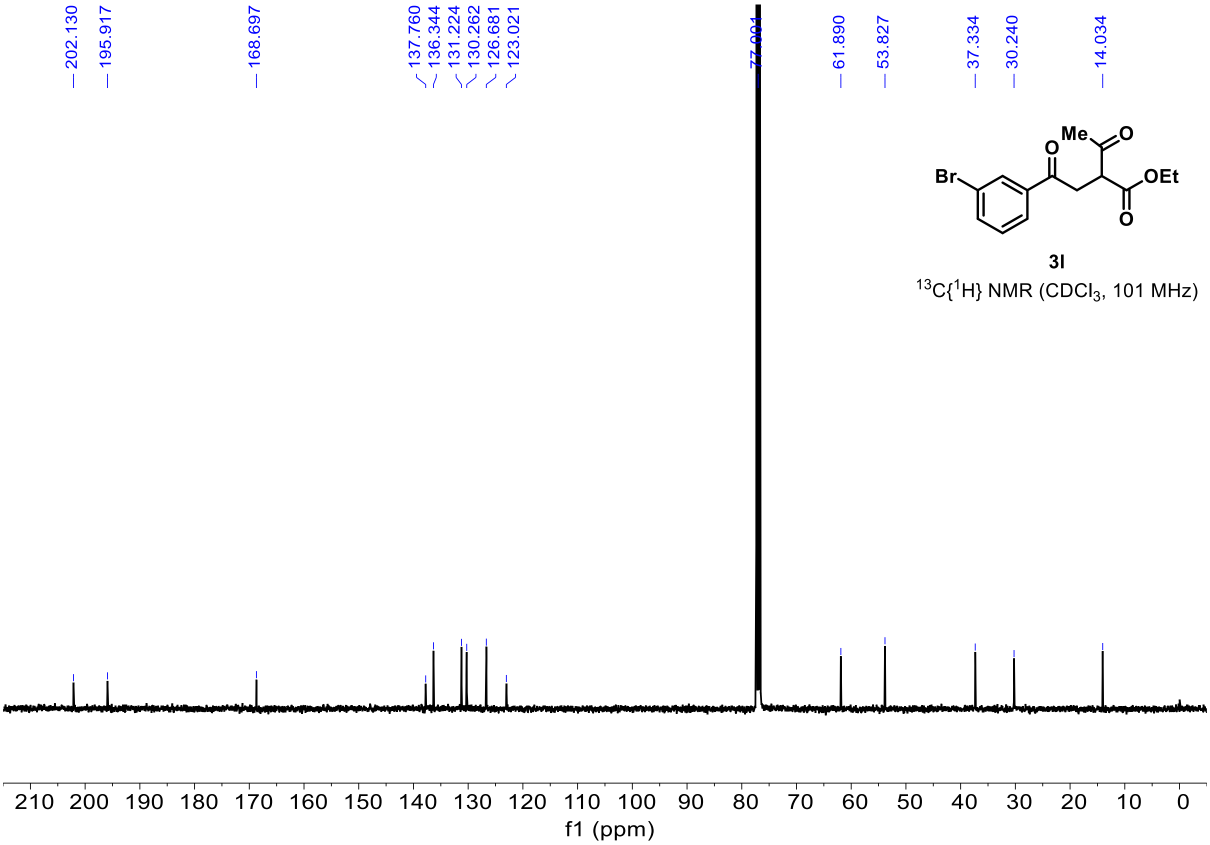

3l

$^{13}C\{^{1}H\}$ NMR ($CDCl_3$, 101 MHz)

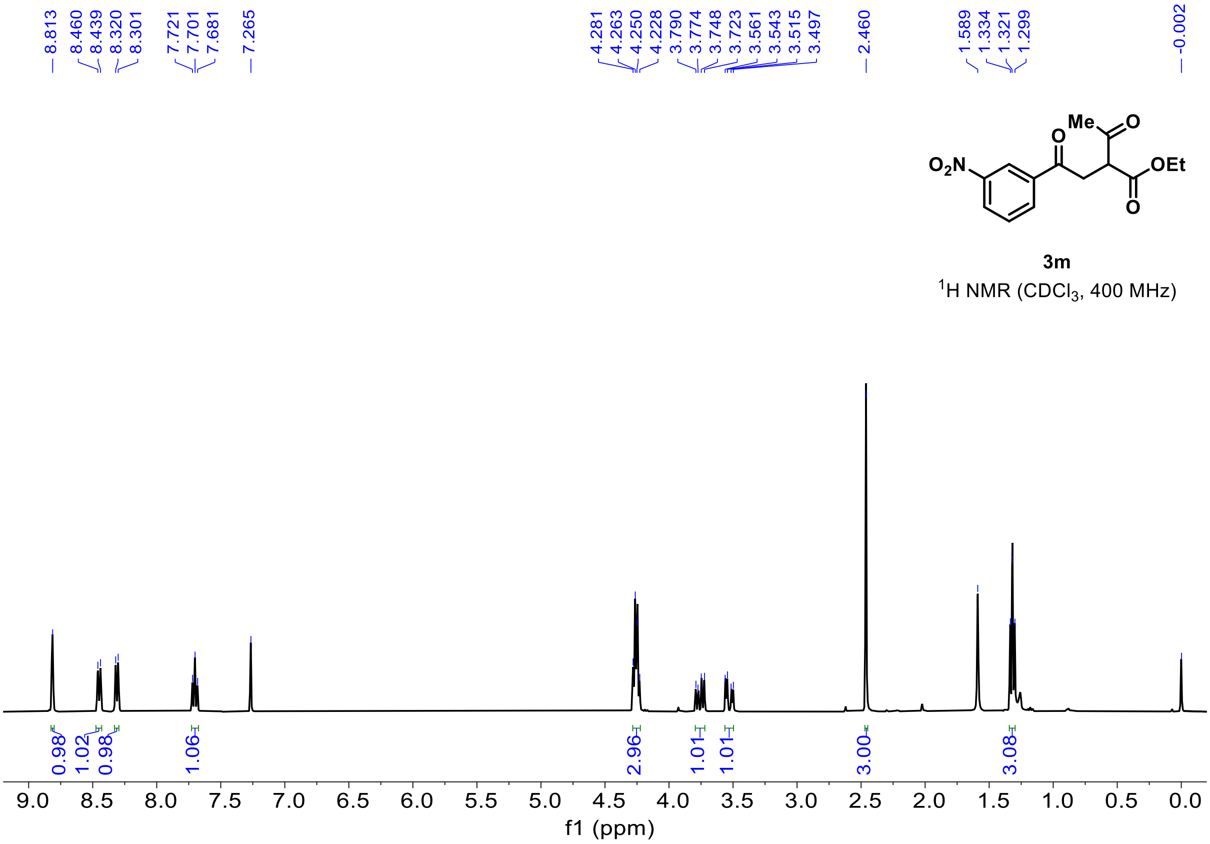

3m

^{1}H NMR ($CDCl_3$, 400 MHz)

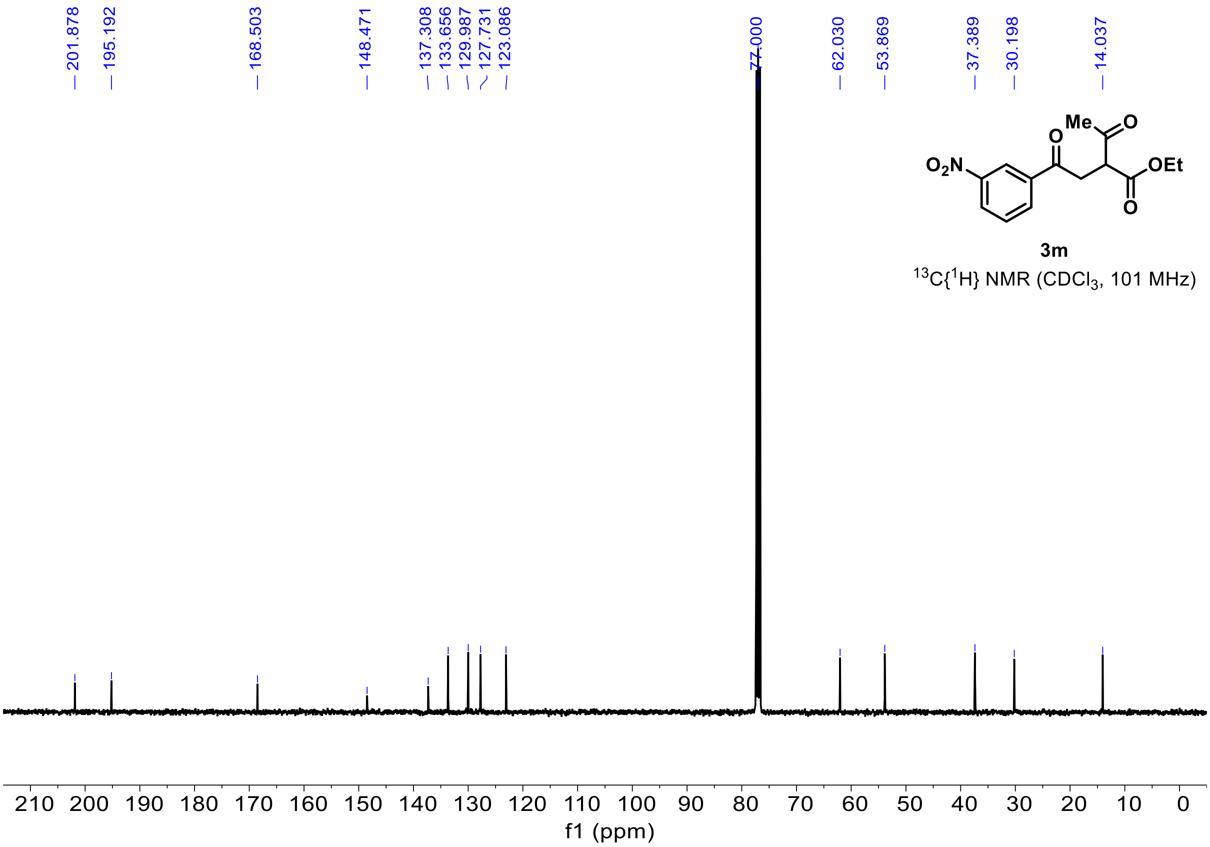

3m

$^{13}C\{^{1}H\}$ NMR ($CDCl_3$, 101 MHz)

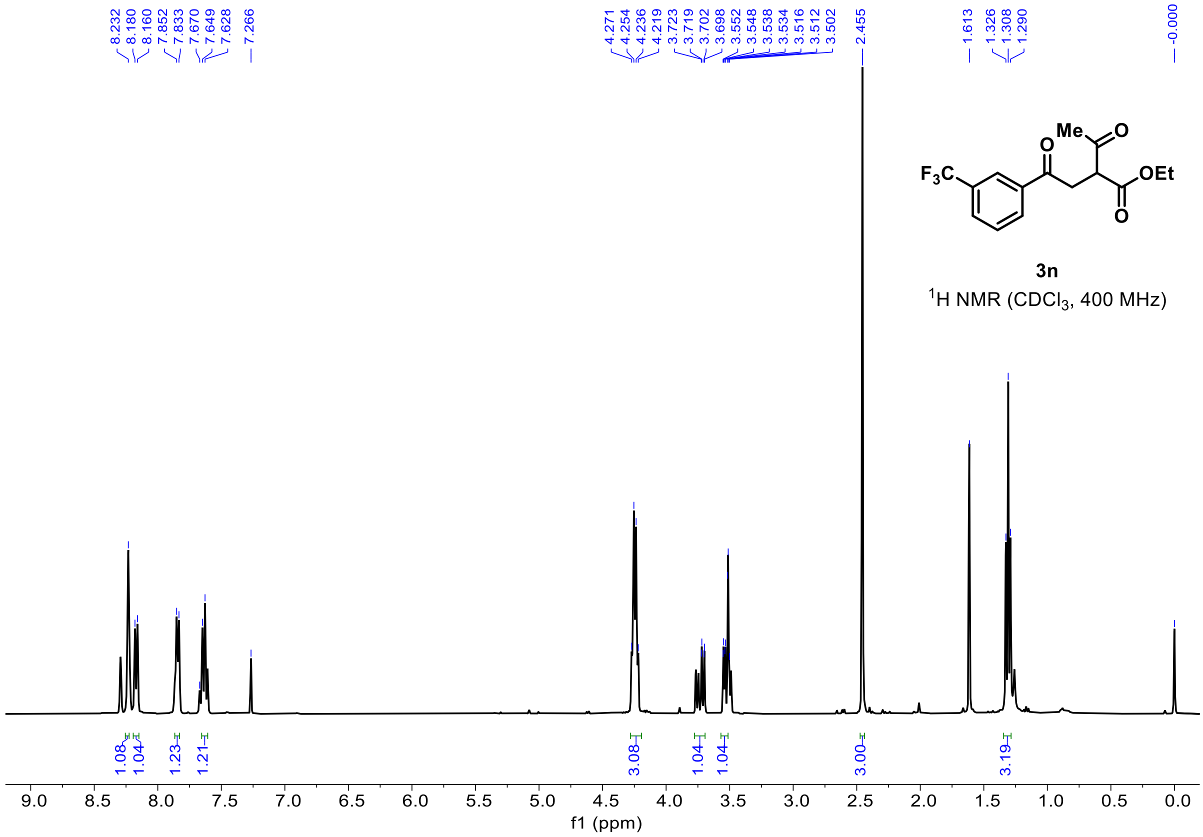
8.232 8.180 8.160 7.852 7.833 7.670 7.649 7.628 7.266
4.271 4.254 4.236 4.219 3.723 3.719 3.702 3.698 3.552 3.548 3.538 3.534 3.516 3.512 3.502
2.455
1.613 1.326 1.308 1.290
-0.000
Me
O
F3C
OEt
3n
1H NMR (CDCl3, 400 MHz)
1.08 1.04 1.23 1.21 3.08 1.04 1.04 3.00 3.19
f1 (ppm)

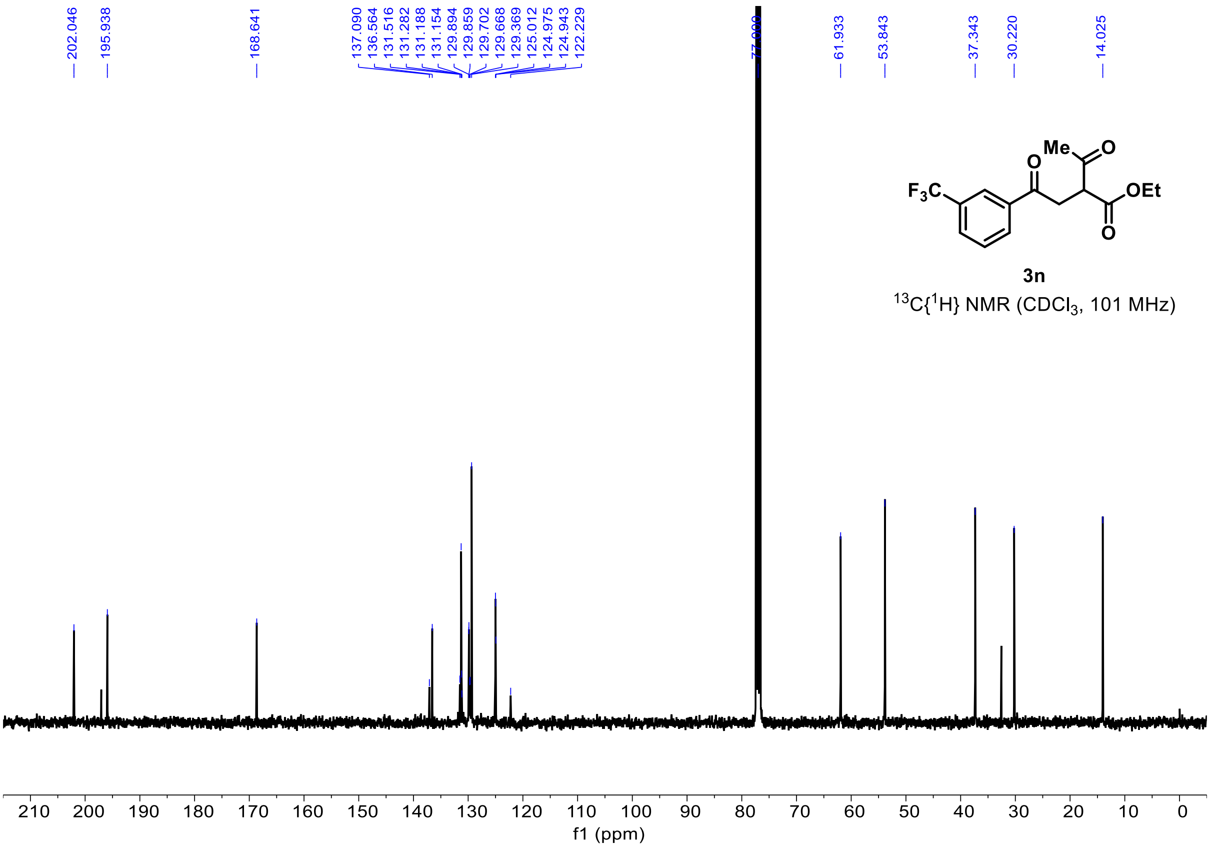
202.046 195.938 168.641
137.090 136.564 131.516 131.282 131.188 131.154 129.894 129.859 129.702 129.668 129.369 125.012 124.975 124.943 122.229
61.933 53.843 37.343 30.220 14.025
Me
O
F3C
OEt
3n
13C{1H} NMR (CDCl3, 101 MHz)
f1 (ppm)

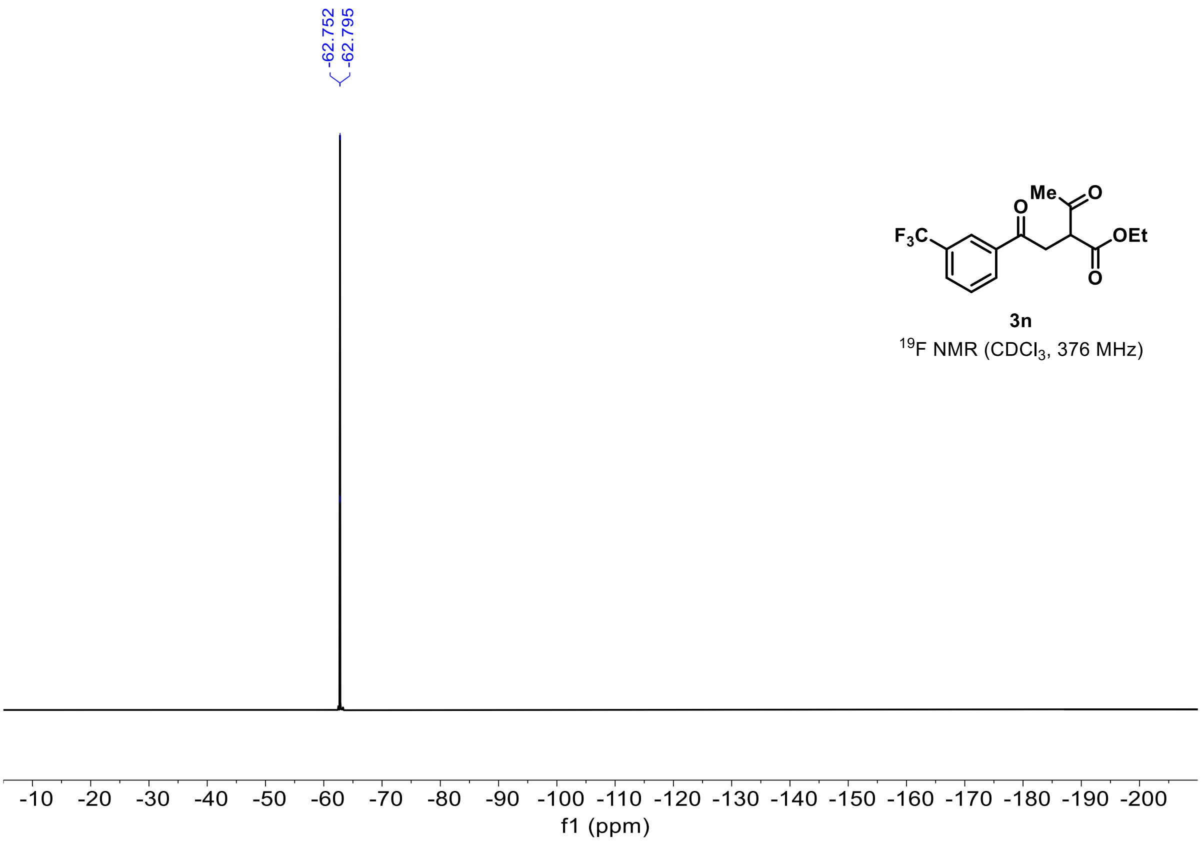

3n

^{19}F NMR ($CDCl_3$, 376 MHz)

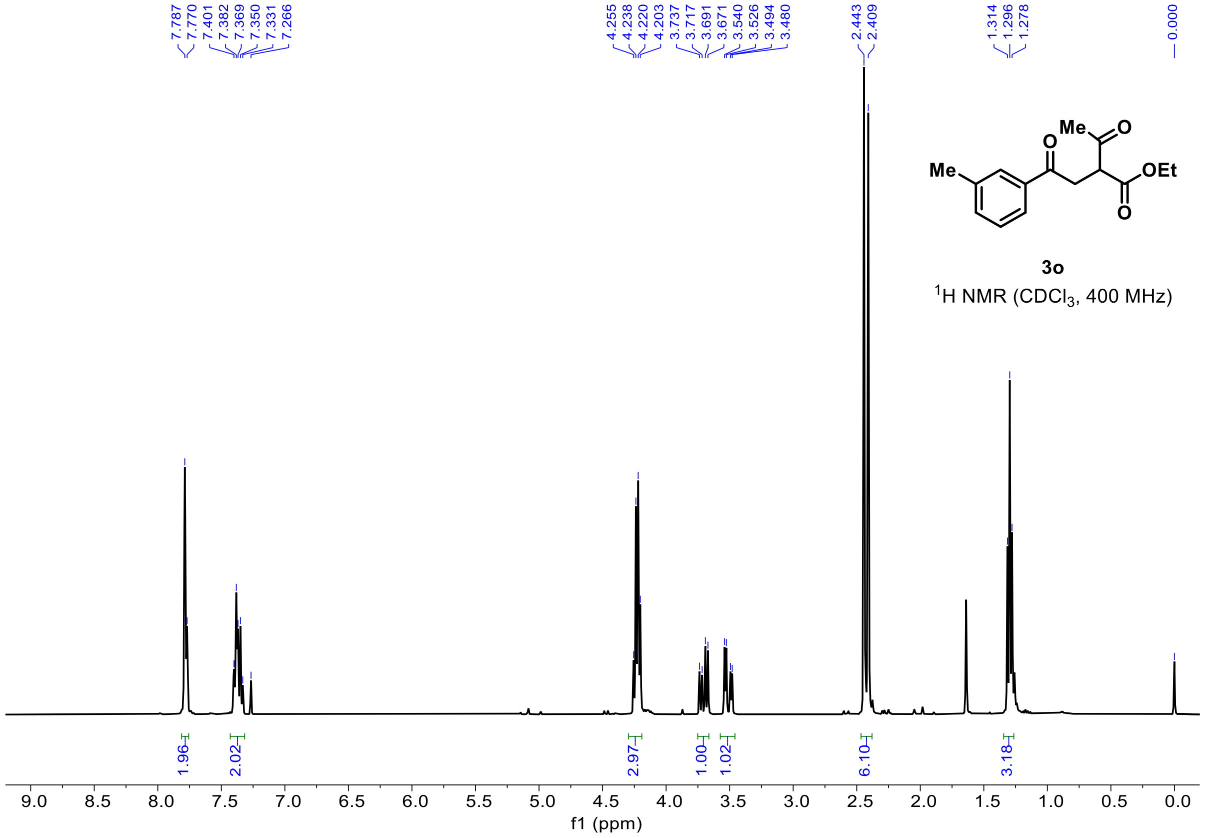

7.787
7.770
7.401
7.382
7.369
7.350
7.331
7.266
4.255
4.238
4.220
4.203
3.737
3.717
3.691
3.671
3.540
3.526
3.494
3.480
2.443
2.409
1.314
1.296
1.278
0.000
Me
O
OEt
3o
^{1}H NMR ($CDCl_3$, 400 MHz)
1.96
2.02
2.97
1.00
1.02
6.10
3.18
f1 (ppm)

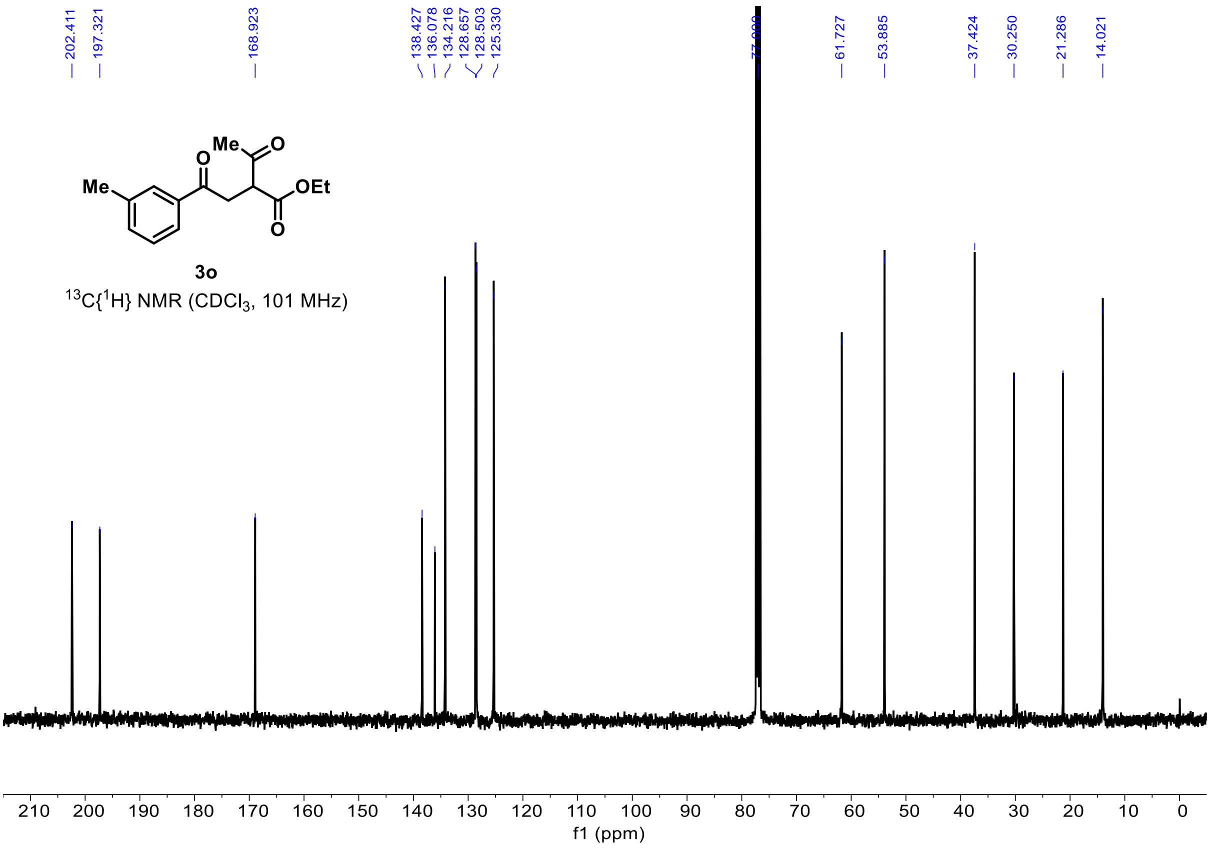

202.411
197.321
168.923
138.427
136.078
134.216
128.657
128.503
125.330
61.727
53.885
37.424
30.250
21.286
14.021
Me
O
OEt
3o
^{13}C{^{1}H} NMR ($CDCl_3$, 101 MHz)
f1 (ppm)

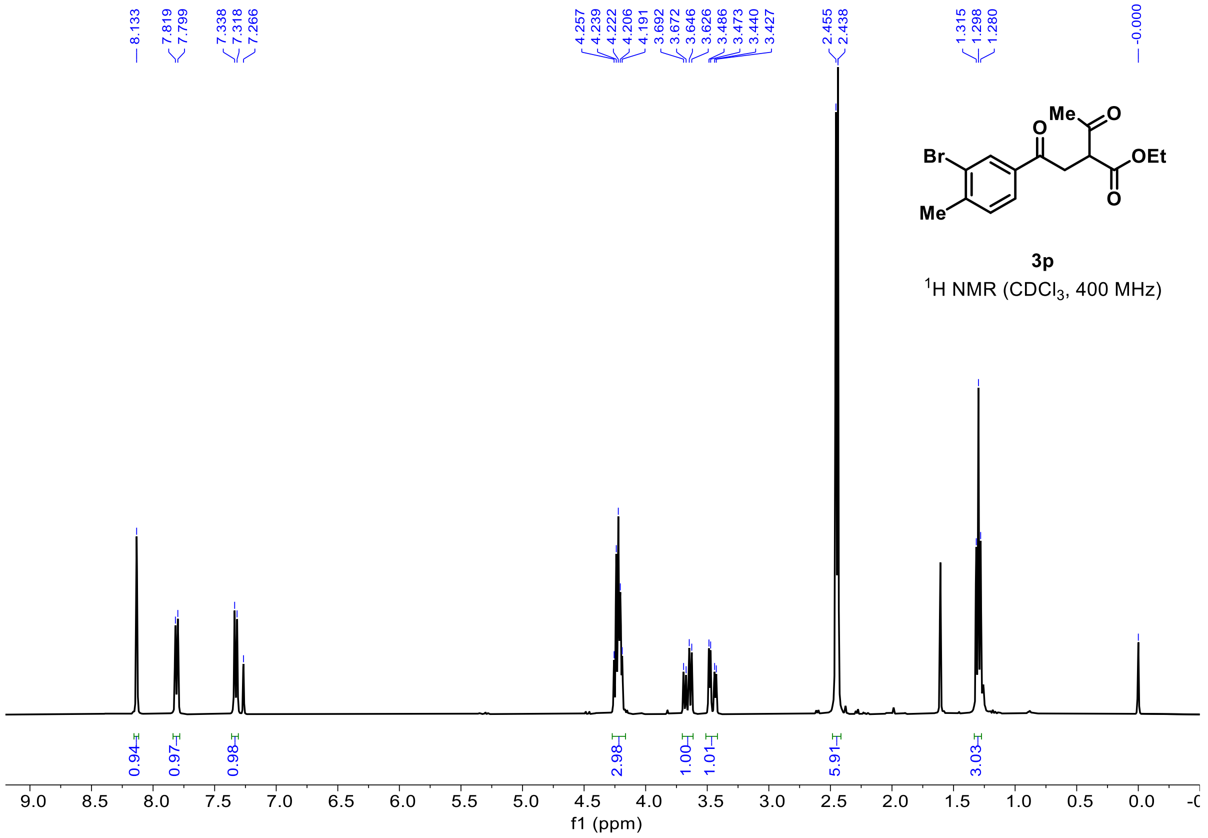

3p
^{1}H NMR (CDCl$_3$, 400 MHz)
f1 (ppm)

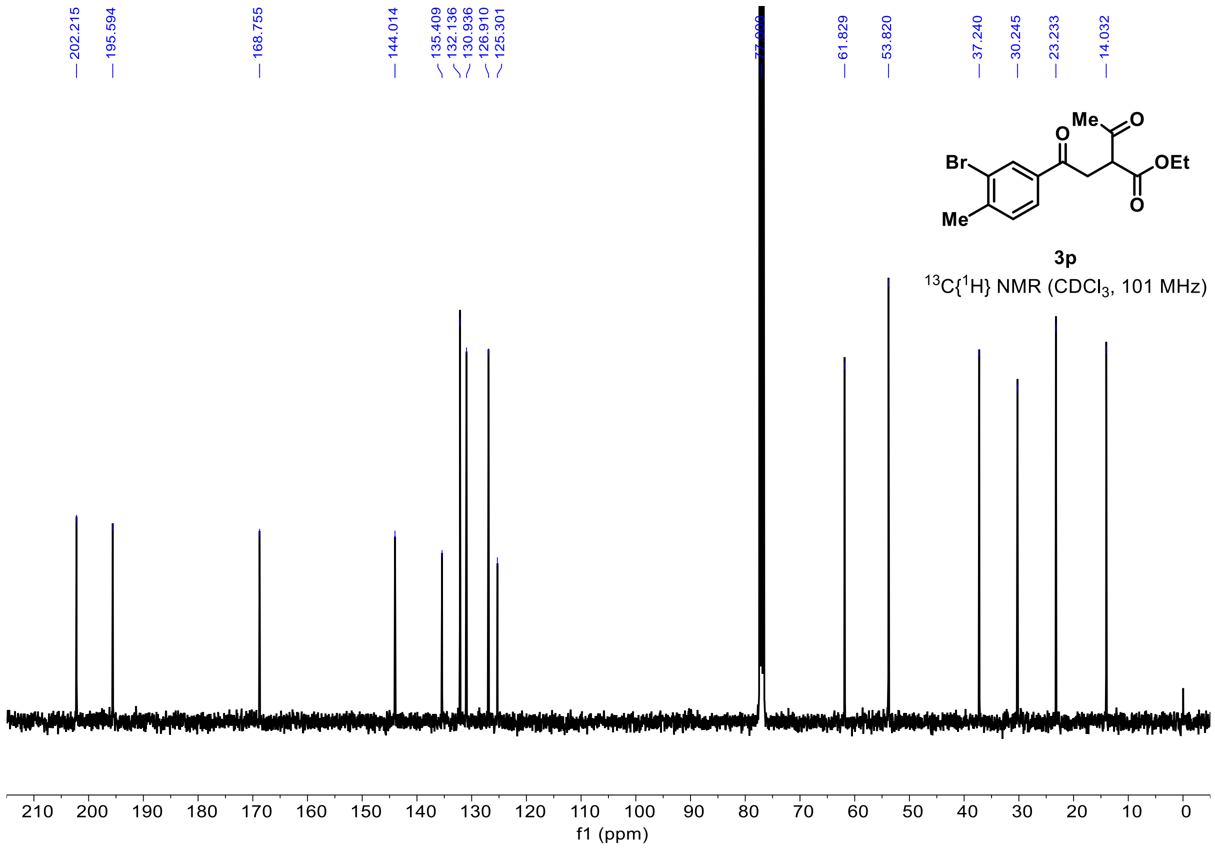

3p
^{13}C{^{1}H} NMR (CDCl$_3$, 101 MHz)
f1 (ppm)

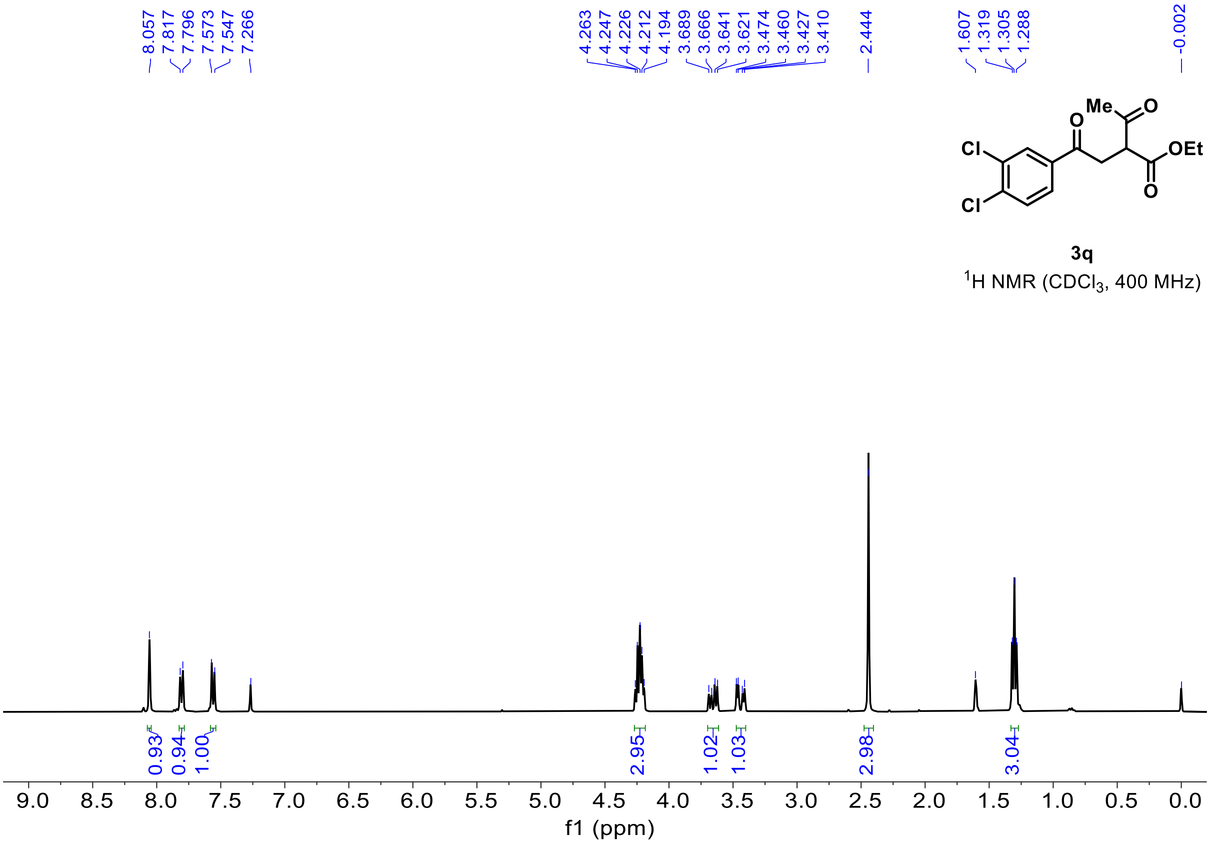

3q

^{1}H NMR ($CDCl_3$, 400 MHz)

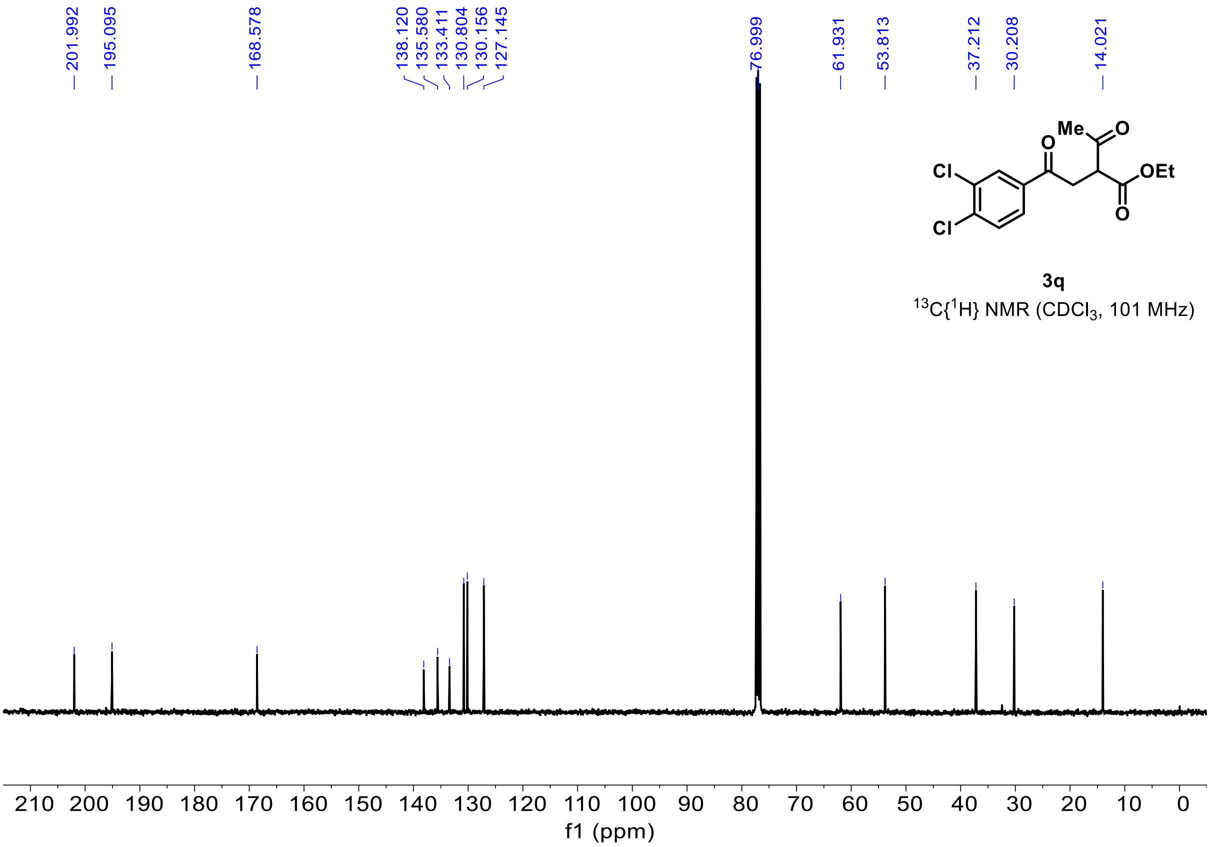

3q

$^{13}C\{^{1}H\}$ NMR ($CDCl_3$, 101 MHz)

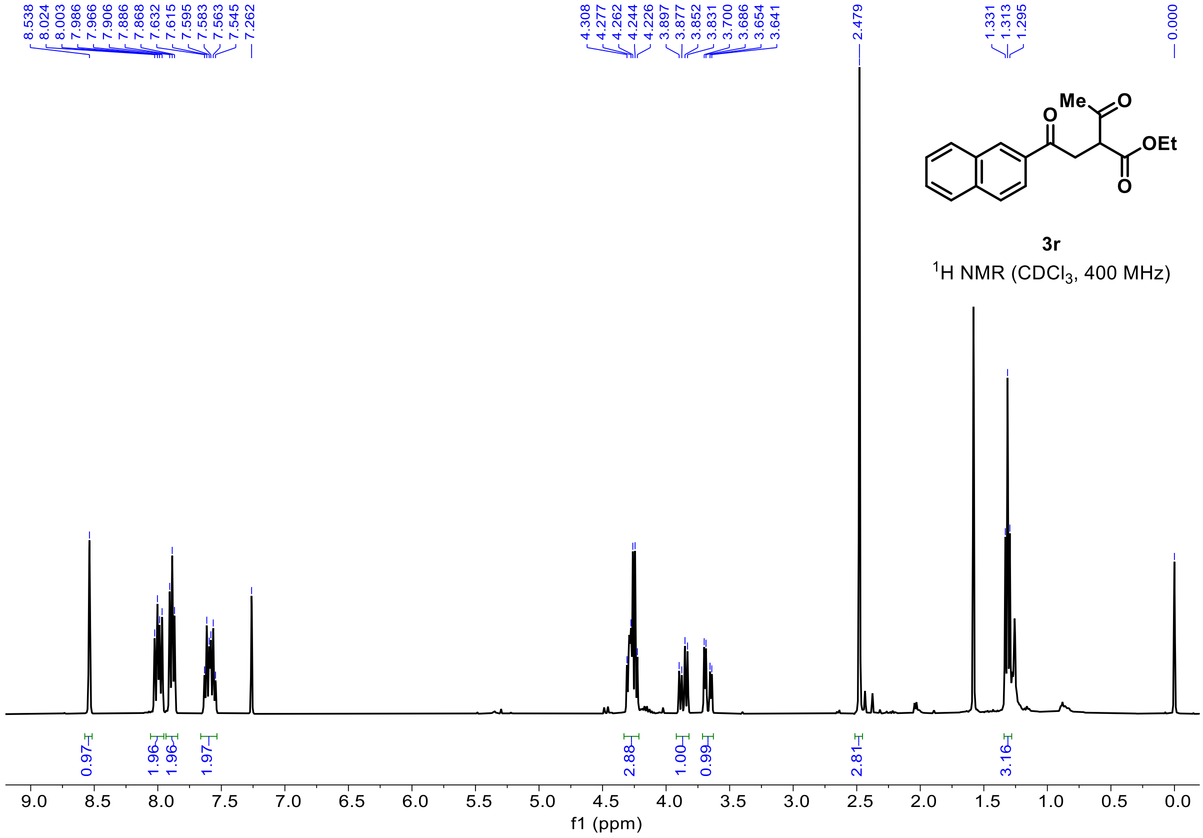
8.538
8.024
8.003
7.986
7.966
7.906
7.886
7.868
7.632
7.615
7.595
7.583
7.563
7.545
7.262
4.308
4.277
4.262
4.244
4.226
3.897
3.877
3.852
3.831
3.700
3.686
3.654
3.641
2.479
1.331
1.313
1.295
0.000
Me
O
O
OEt
O
3r
^{1}H NMR (CDCl$_3$, 400 MHz)
0.97
1.96
1.96
1.97
2.88
1.00
0.99
2.81
3.16
9.0 8.5 8.0 7.5 7.0 6.5 6.0 5.5 5.0 4.5 4.0 3.5 3.0 2.5 2.0 1.5 1.0 0.5 0.0
f1 (ppm)

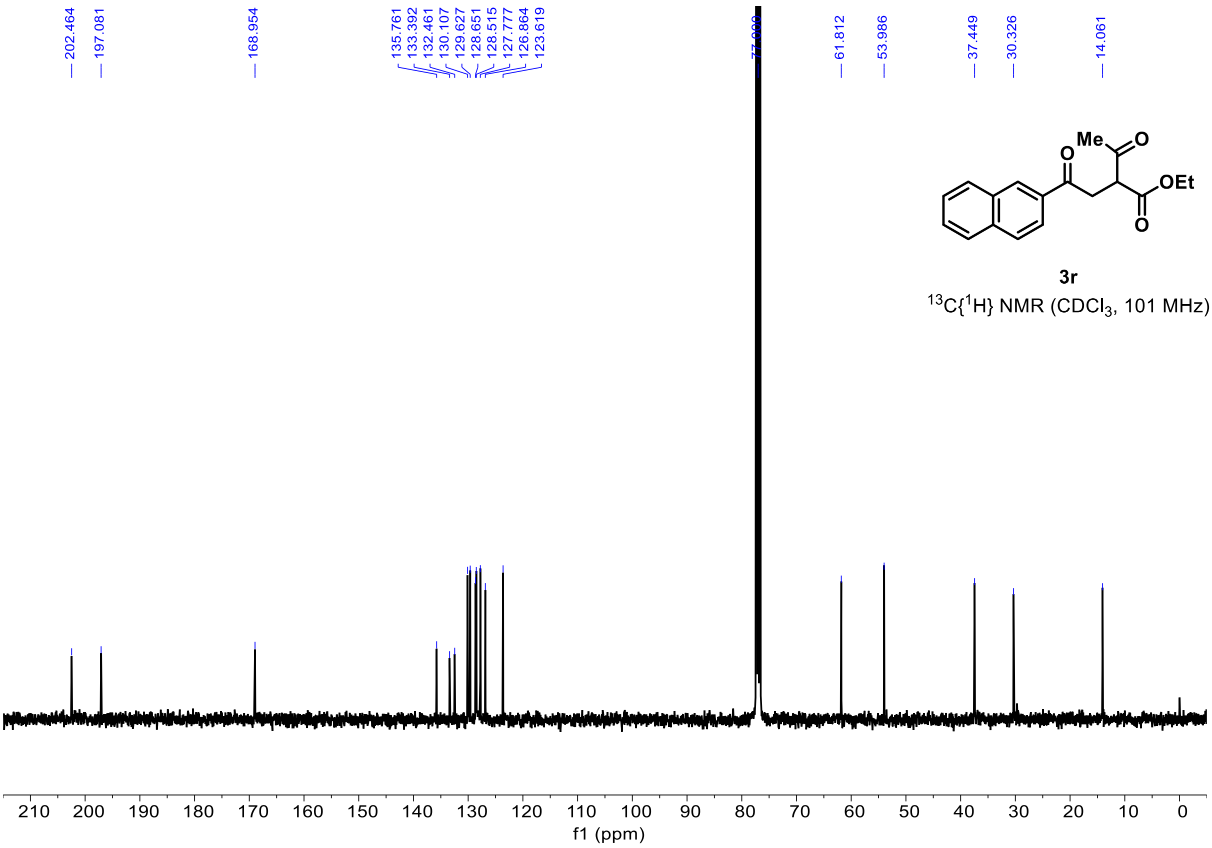
202.464
197.081
168.954
135.761
133.392
132.461
130.107
129.627
128.651
128.515
127.777
126.864
123.619
61.812
53.986
37.449
30.326
14.061
Me
O
O
OEt
O
3r
^{13}C{^{1}H} NMR (CDCl$_3$, 101 MHz)
210 200 190 180 170 160 150 140 130 120 110 100 90 80 70 60 50 40 30 20 10 0
f1 (ppm)

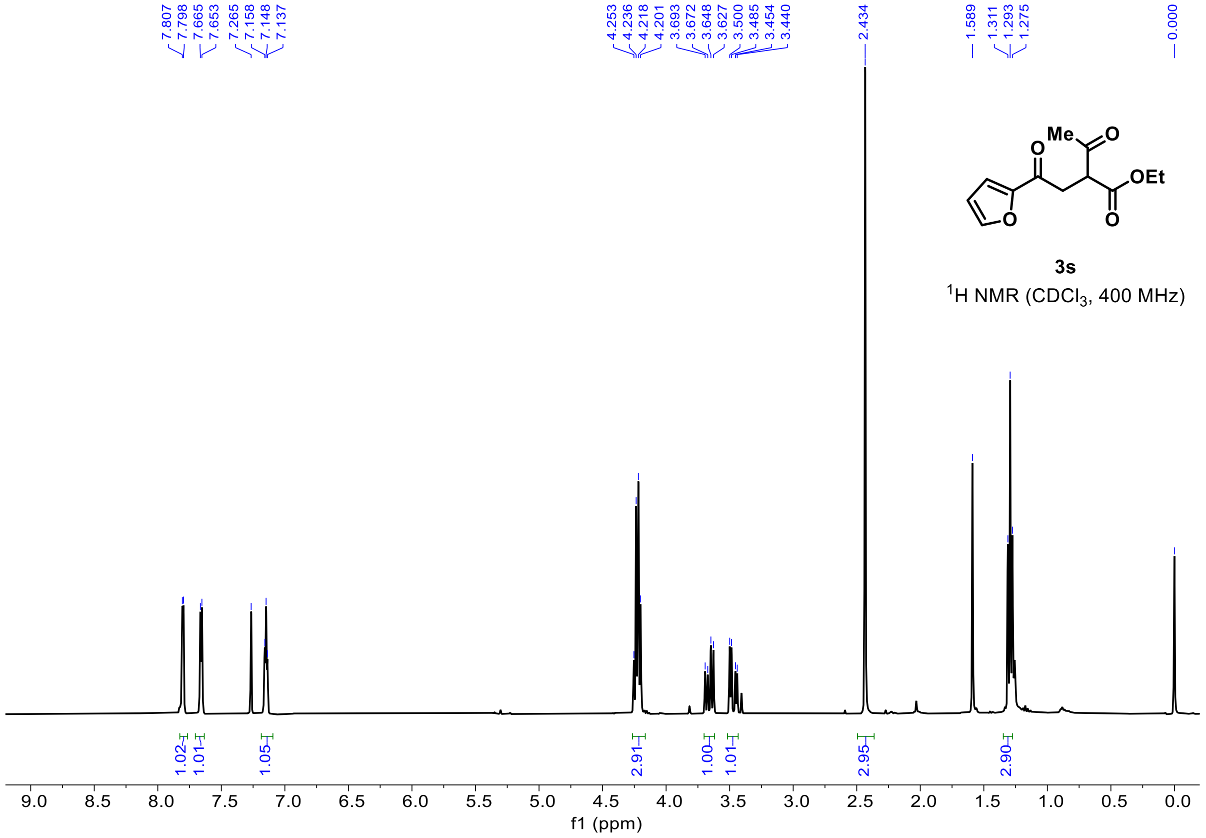
3s
1H NMR (CDCl3, 400 MHz)
f1 (ppm)

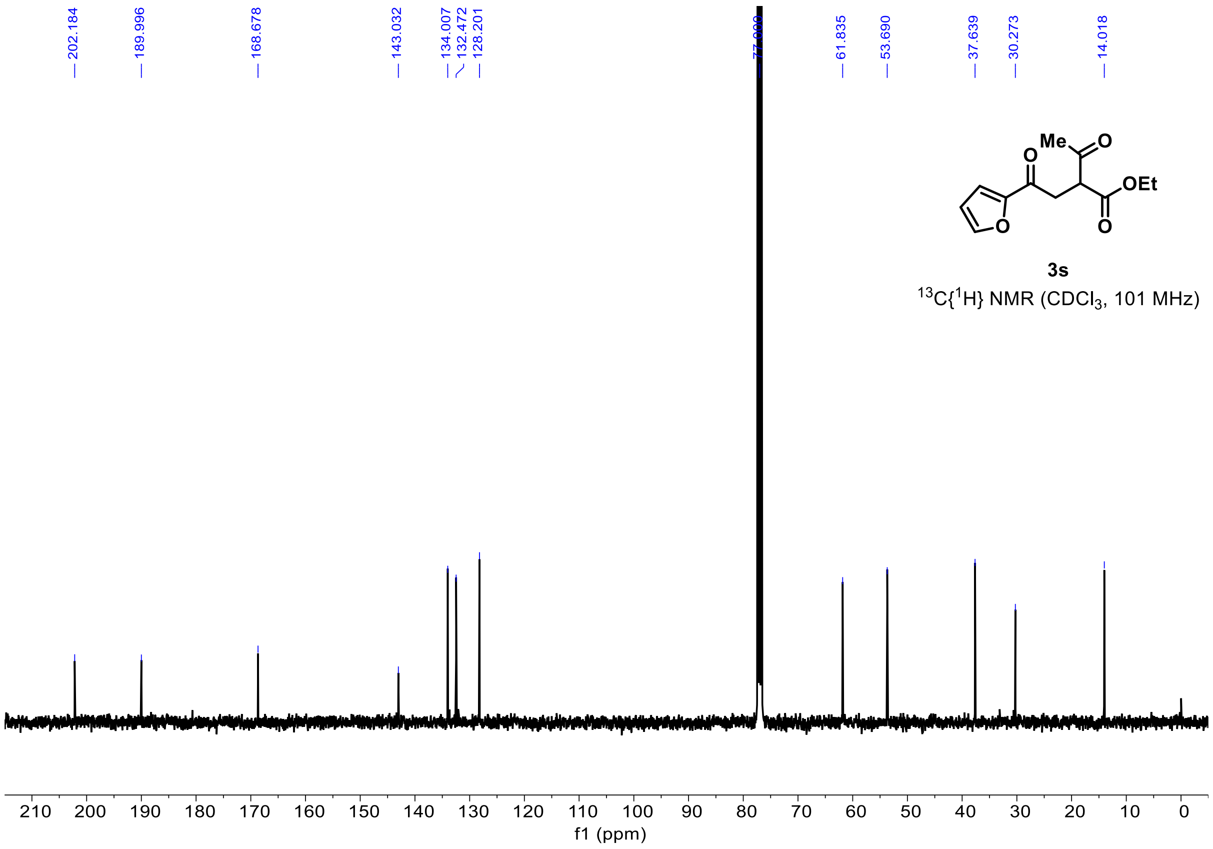
3s
13C{1H} NMR (CDCl3, 101 MHz)
f1 (ppm)

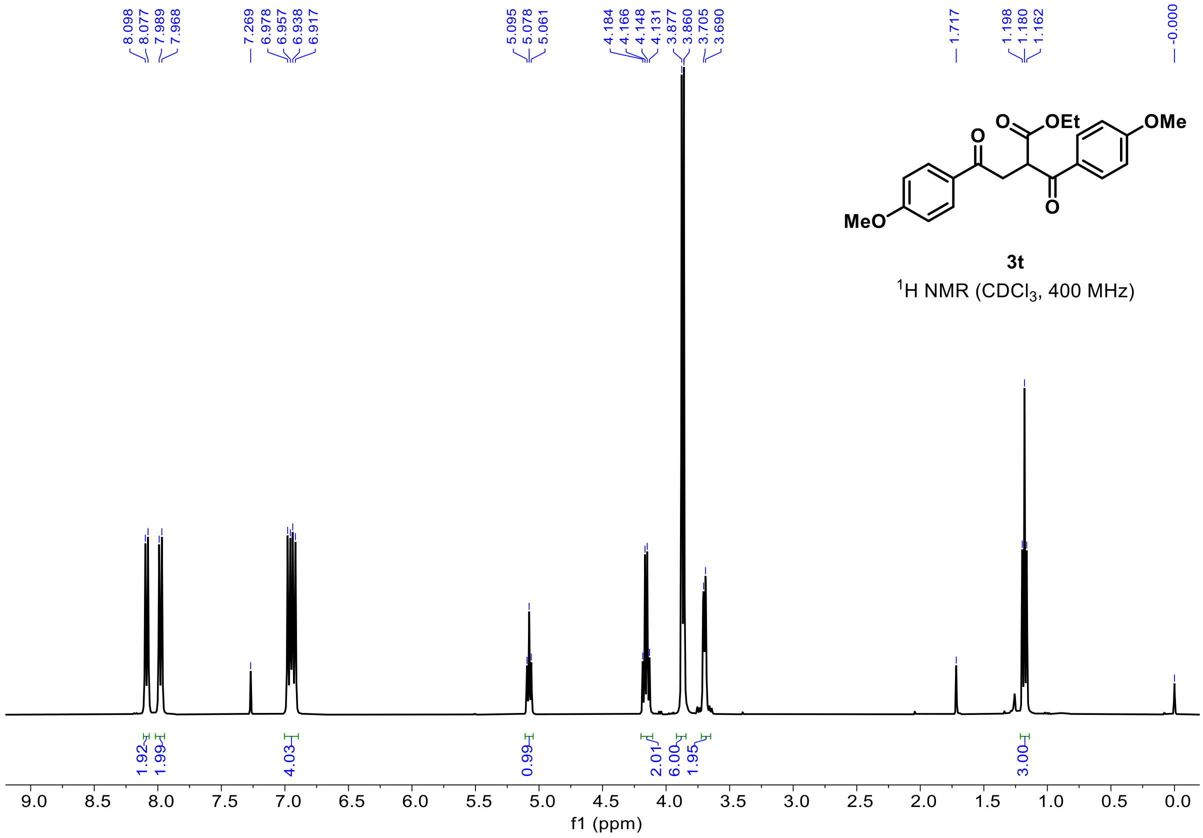
8.098
8.077
7.989
7.968
7.269
6.978
6.957
6.938
6.917
5.095
5.078
5.061
4.184
4.166
4.148
4.131
3.877
3.860
3.705
3.690
1.717
1.198
1.180
1.162
-0.000
OEt
OMe
MeO
3t
1H NMR (CDCl3, 400 MHz)
1.92
1.99
4.03
0.99
2.01
6.00
1.95
3.00
f1 (ppm)

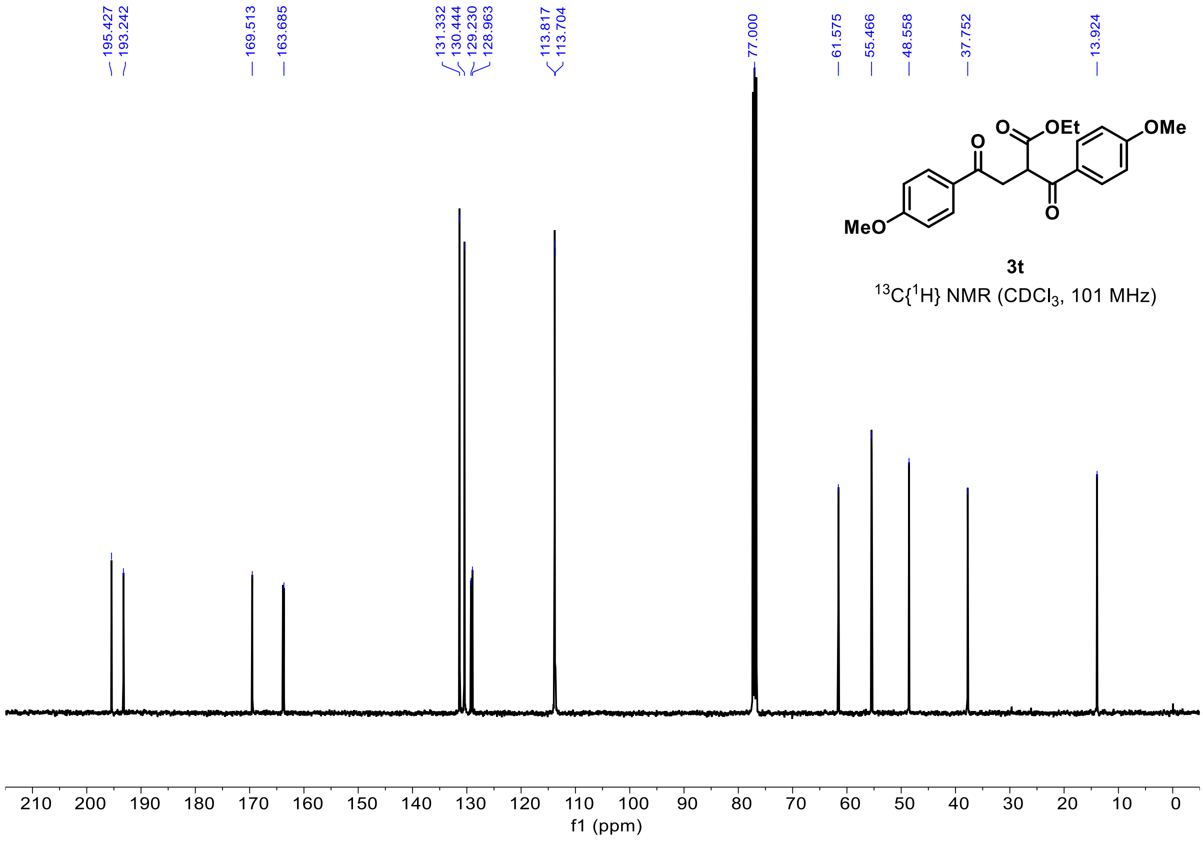
195.427
193.242
169.513
163.685
131.332
130.444
129.230
128.963
113.817
113.704
77.000
61.575
55.466
48.558
37.752
13.924
OEt
OMe
MeO
3t
13C{1H} NMR (CDCl3, 101 MHz)
f1 (ppm)

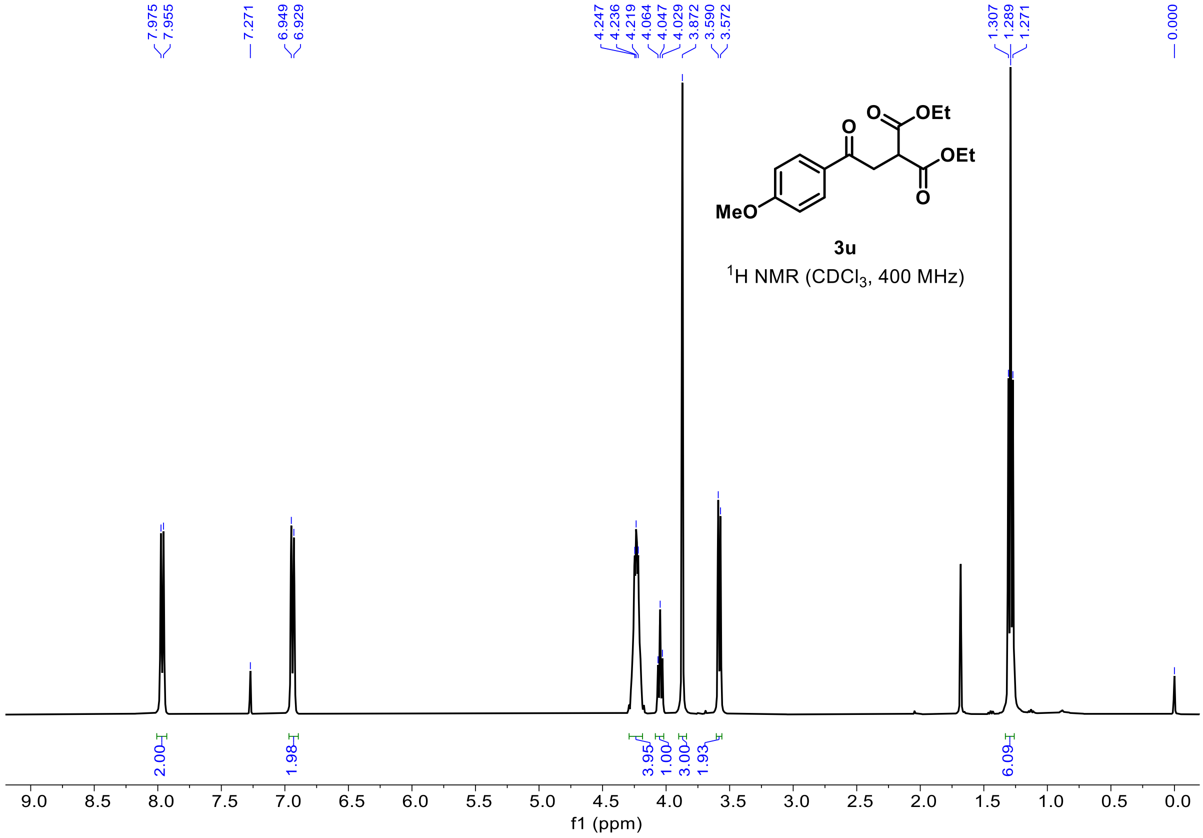
7.975
7.955
7.271
6.949
6.929
4.247
4.236
4.219
4.064
4.047
4.029
3.872
3.590
3.572
1.307
1.289
1.271
0.000
O
OEt
O
OEt
O
MeO
3u
1H NMR (CDCl3, 400 MHz)
2.00
1.98
3.95
1.00
3.00
1.93
6.09
9.0 8.5 8.0 7.5 7.0 6.5 6.0 5.5 5.0 4.5 4.0 3.5 3.0 2.5 2.0 1.5 1.0 0.5 0.0
f1 (ppm)

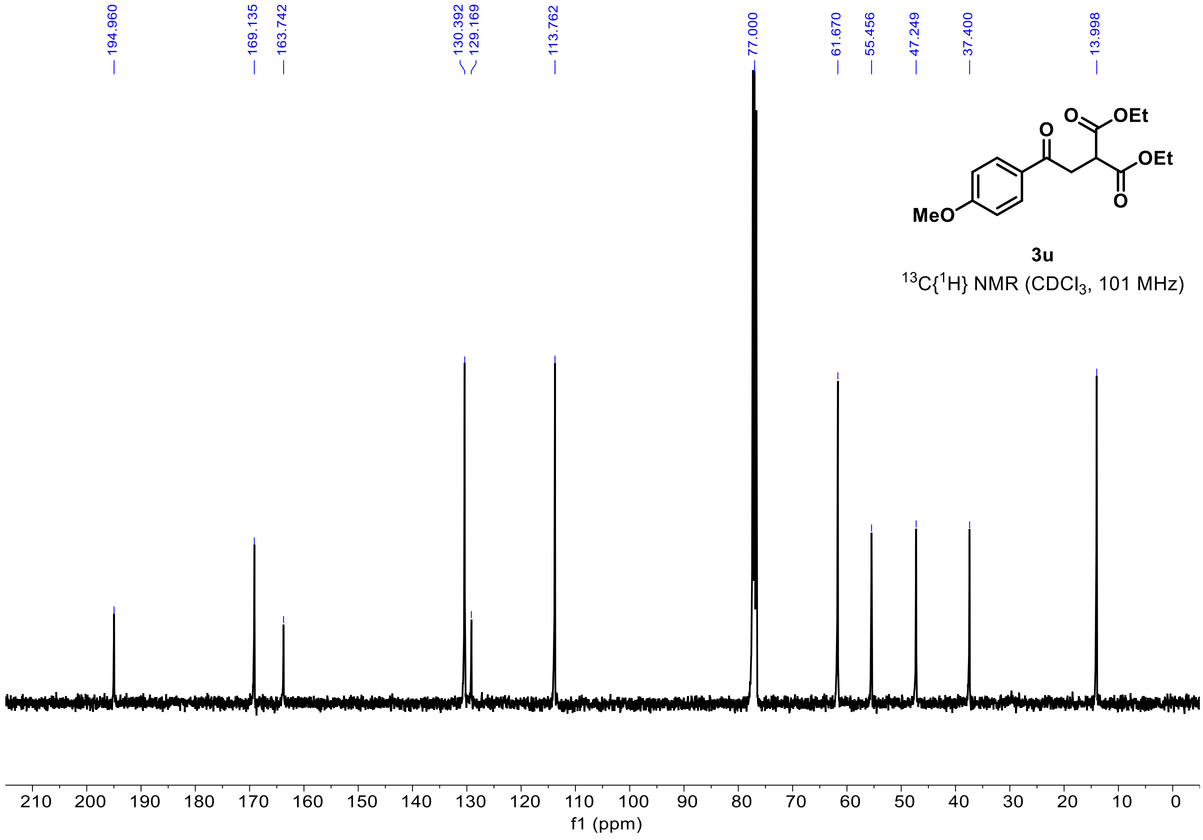
194.960
169.135
163.742
130.392
129.169
113.762
77.000
61.670
55.456
47.249
37.400
13.998
O
OEt
O
OEt
O
MeO
3u
13C{1H} NMR (CDCl3, 101 MHz)
210 200 190 180 170 160 150 140 130 120 110 100 90 80 70 60 50 40 30 20 10 0
f1 (ppm)

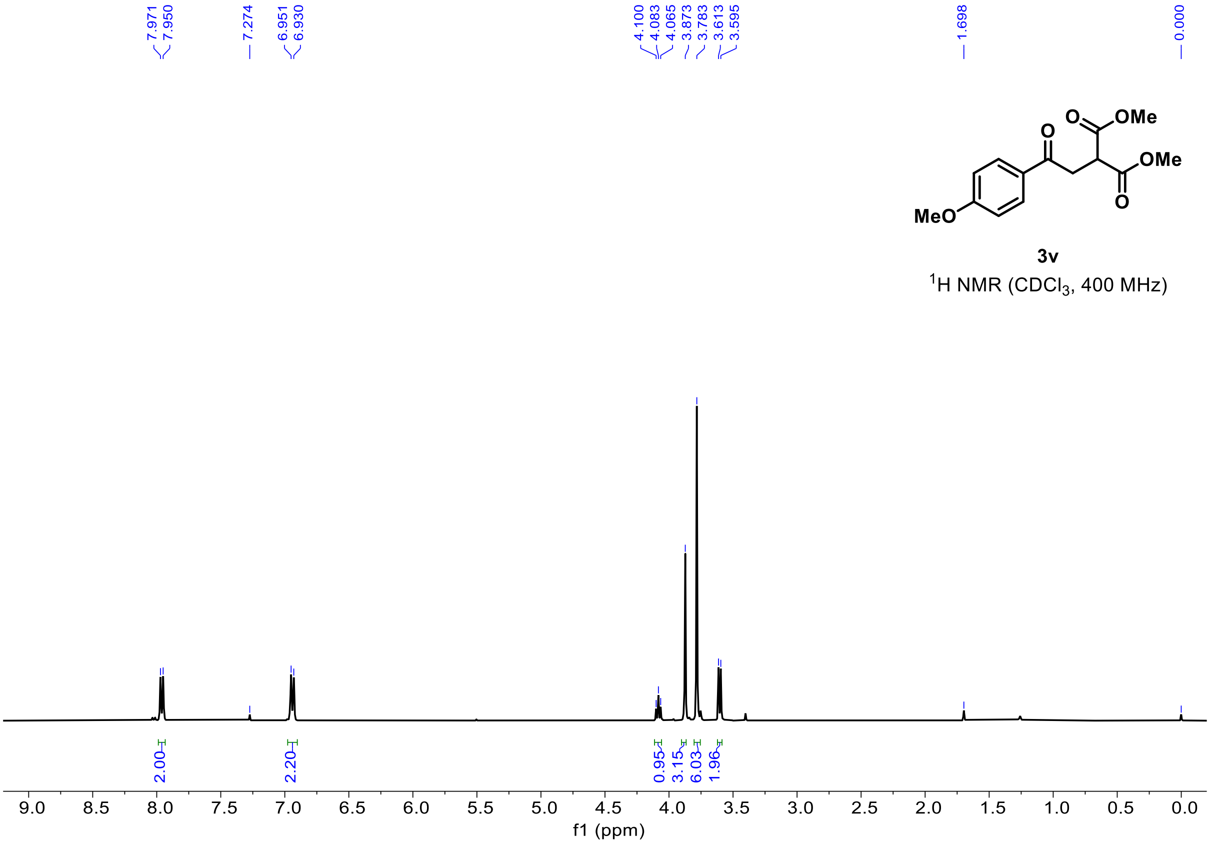
7.971
7.950
7.274
6.951
6.930
4.100
4.083
4.065
3.873
3.783
3.613
3.595
1.698
0.000
O
OMe
O
OMe
O
O
MeO
3v
^{1}H NMR (CDCl$_3$, 400 MHz)
2.00
2.20
0.95
3.15
6.03
1.96
9.0 8.5 8.0 7.5 7.0 6.5 6.0 5.5 5.0 4.5 4.0 3.5 3.0 2.5 2.0 1.5 1.0 0.5 0.0
f1 (ppm)

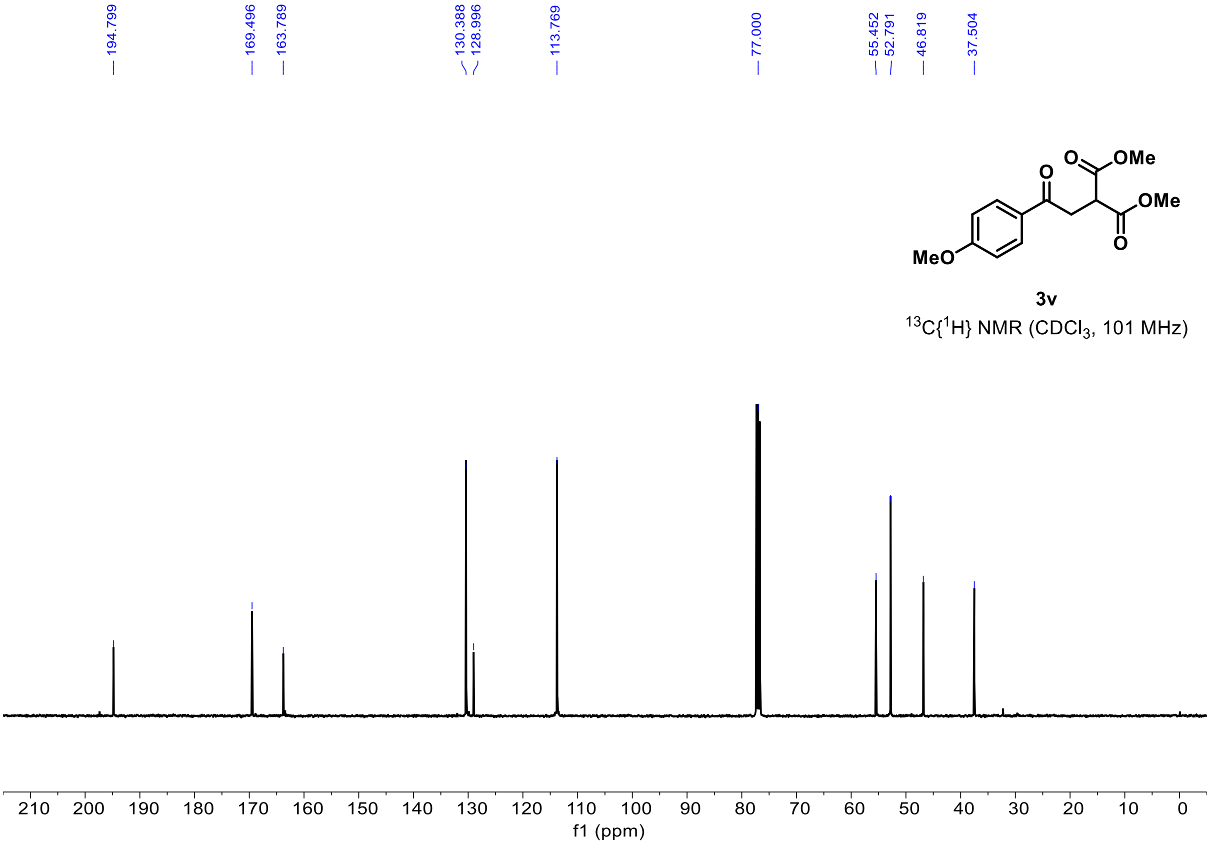
194.799
169.496
163.789
130.388
128.996
113.769
77.000
55.452
52.791
46.819
37.504
O
OMe
O
OMe
O
O
MeO
3v
^{13}C{^{1}H} NMR (CDCl$_3$, 101 MHz)
210 200 190 180 170 160 150 140 130 120 110 100 90 80 70 60 50 40 30 20 10 0
f1 (ppm)

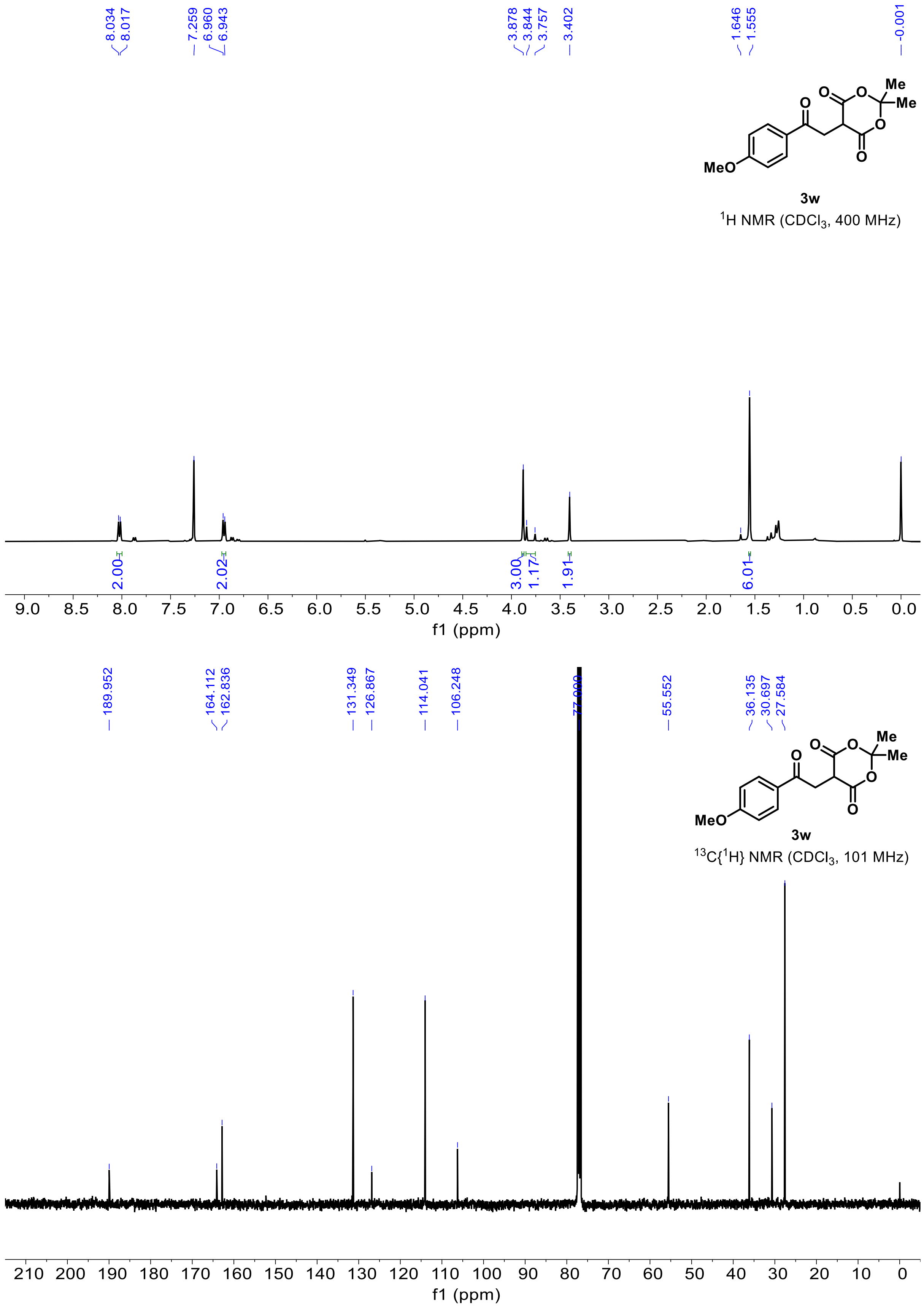
8.034
8.017
7.259
6.960
6.943
3.878
3.844
3.757
3.402
1.646
1.555
-0.001
3w
^{1}H NMR (CDCl$_3$, 400 MHz)
2.00
2.02
3.00
1.17
1.91
6.01
f1 (ppm)
189.952
164.112
162.836
131.349
126.867
114.041
106.248
77.000
55.552
36.135
30.697
27.584
3w
^{13}C{^{1}H} NMR (CDCl$_3$, 101 MHz)
f1 (ppm)

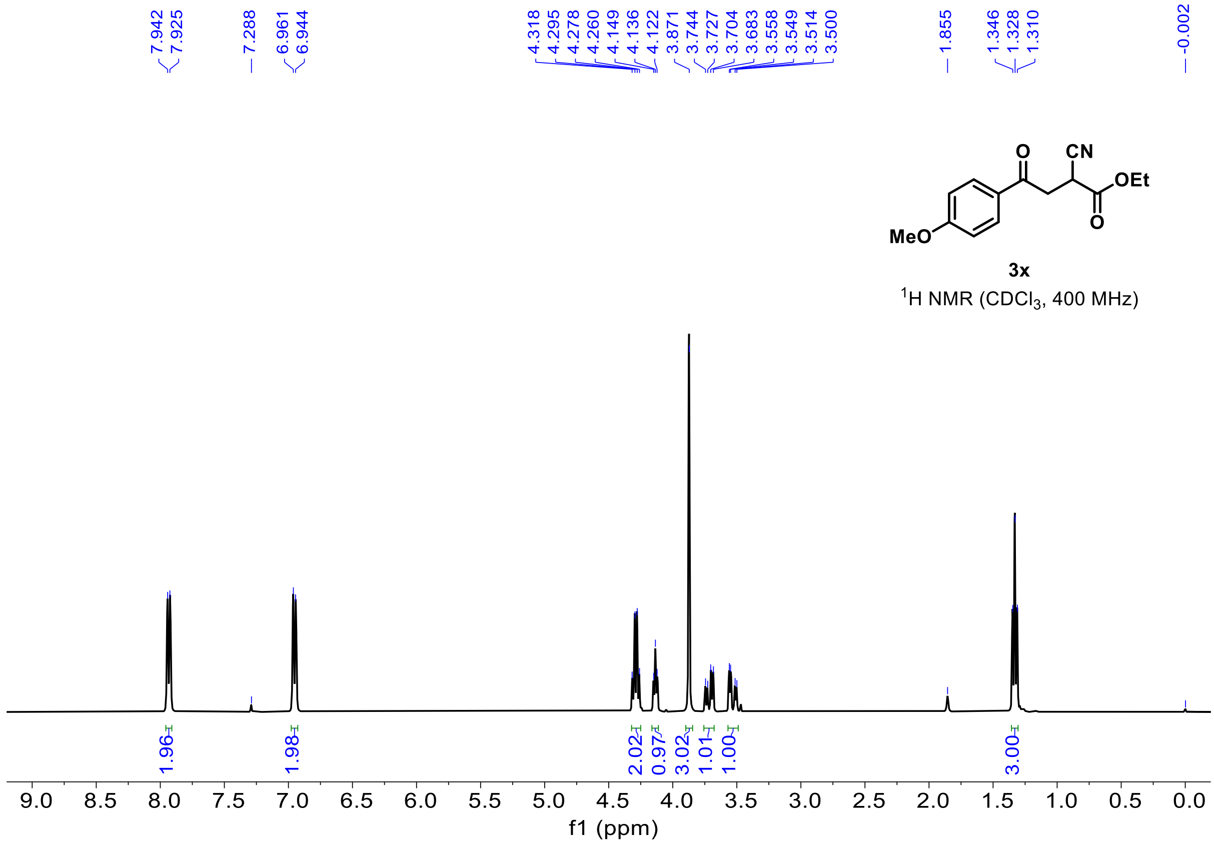
O
CN
OEt
O
MeO
3x
1H NMR (CDCl3, 400 MHz)
f1 (ppm)

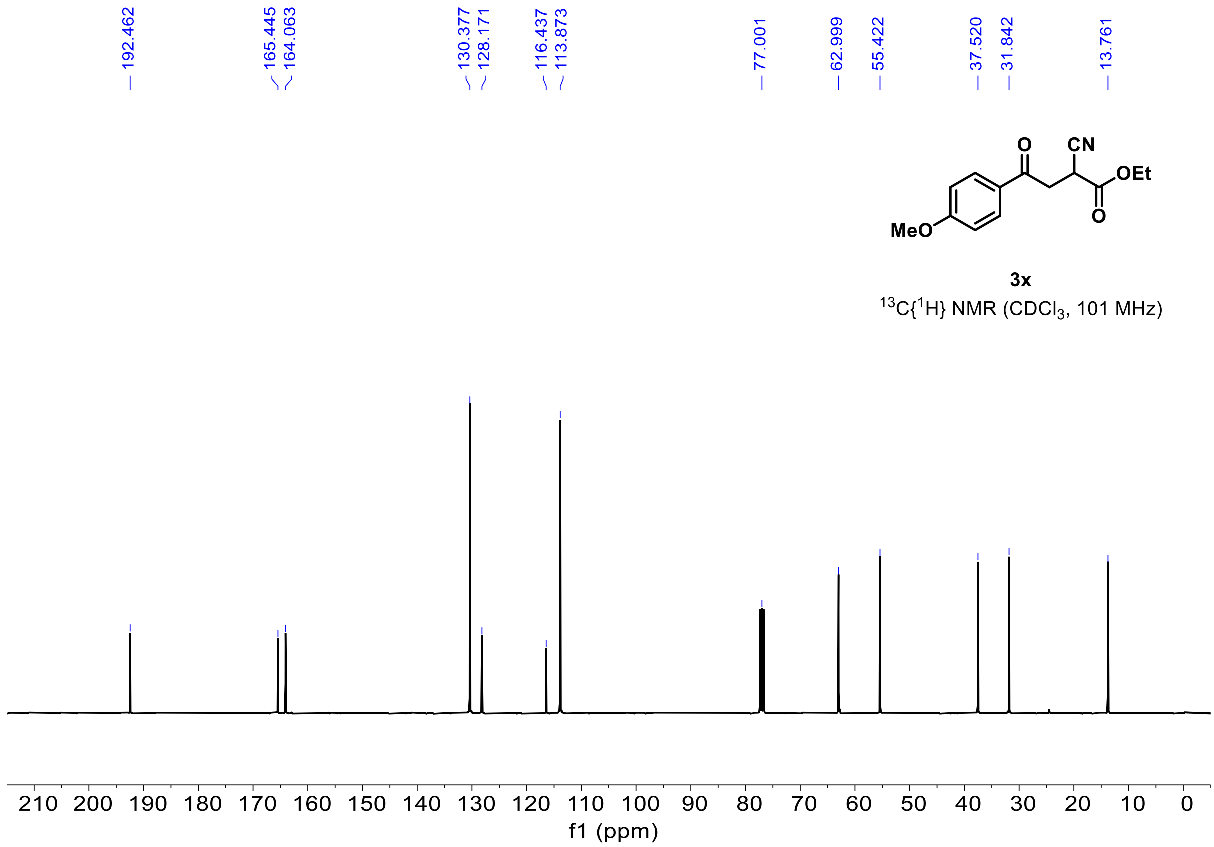
O
CN
OEt
O
MeO
3x
13C{1H} NMR (CDCl3, 101 MHz)
f1 (ppm)

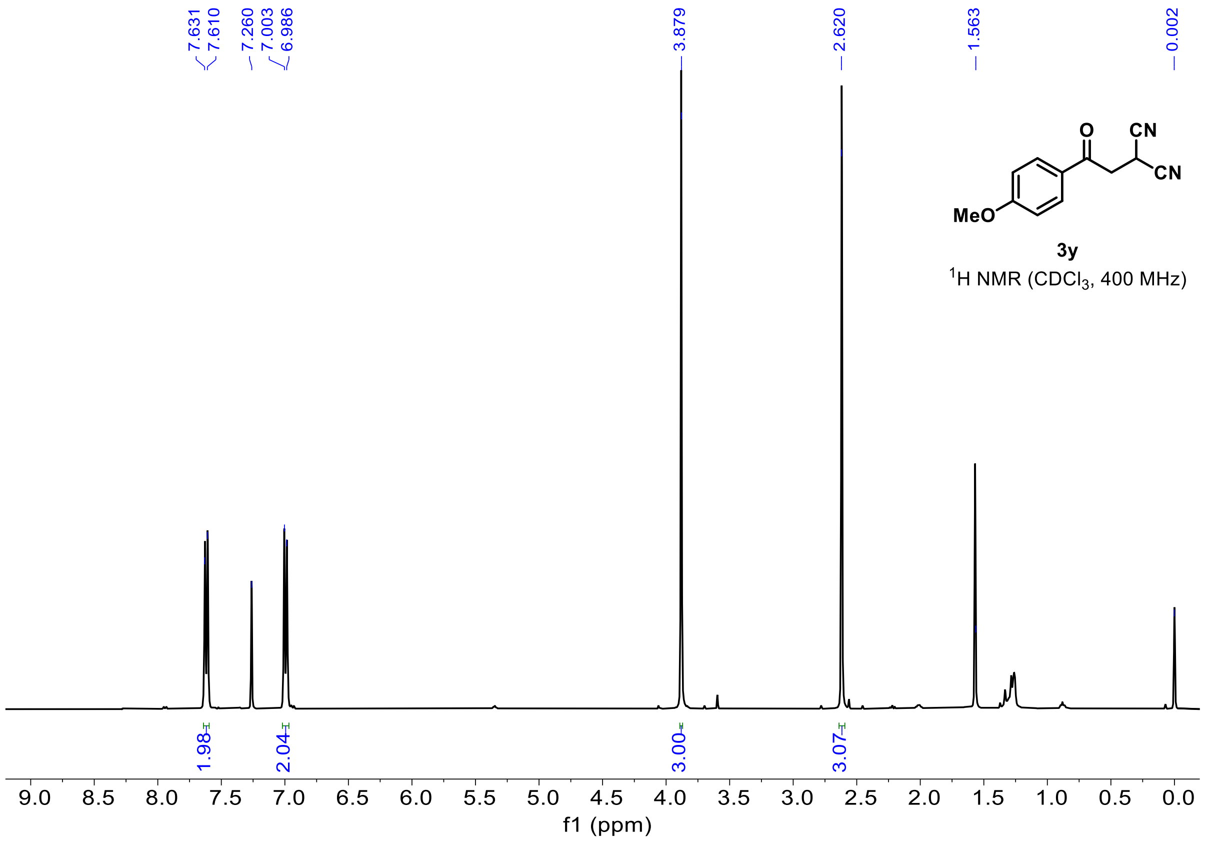
7.631
7.610
7.260
7.003
6.986
3.879
2.620
1.563
0.002
O
CN
CN
MeO
3y
^{1}H NMR ($CDCl_3$, 400 MHz)
1.98
2.04
3.00
3.07
9.0 8.5 8.0 7.5 7.0 6.5 6.0 5.5 5.0 4.5 4.0 3.5 3.0 2.5 2.0 1.5 1.0 0.5 0.0
f1 (ppm)

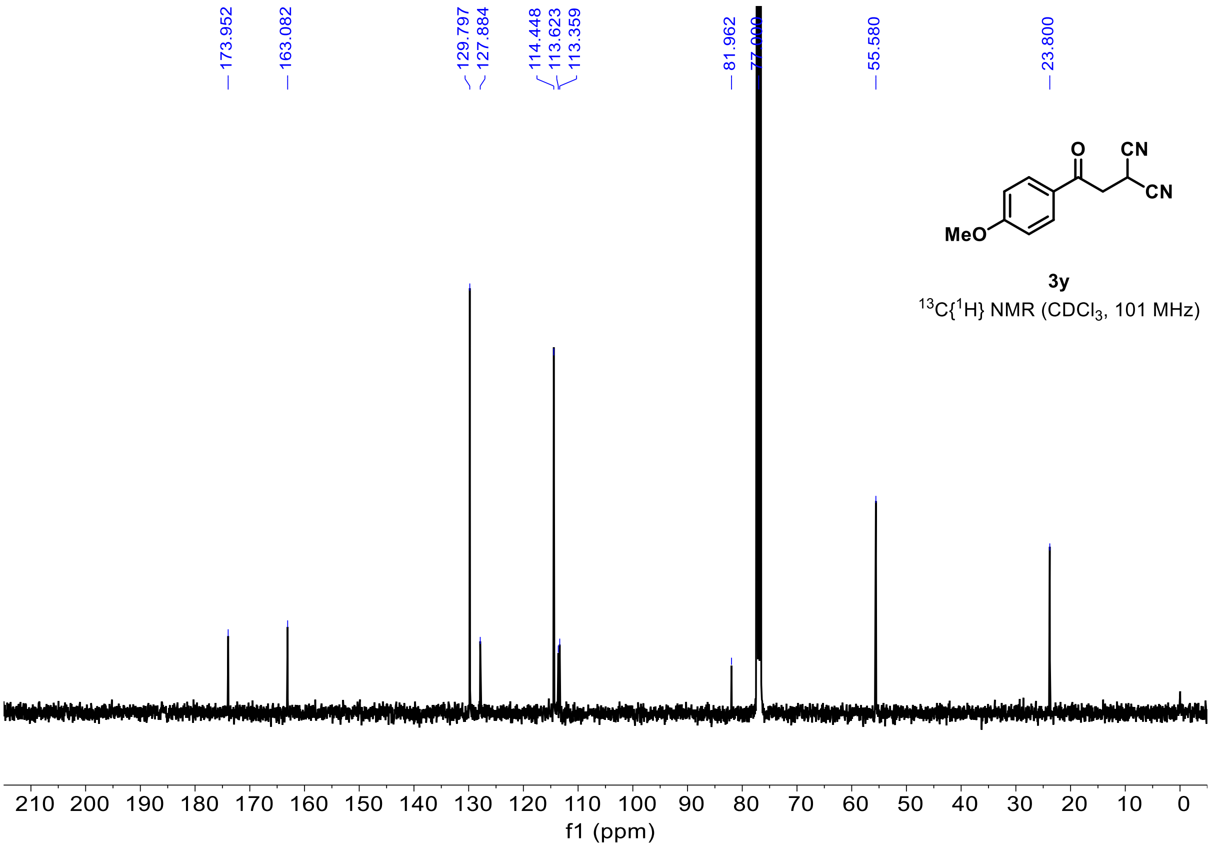
173.952
163.082
129.797
127.884
114.448
113.623
113.359
81.962
77.000
55.580
23.800
O
CN
CN
MeO
3y
$^{13}C\{^{1}H\}$ NMR ($CDCl_3$, 101 MHz)
210 200 190 180 170 160 150 140 130 120 110 100 90 80 70 60 50 40 30 20 10 0
f1 (ppm)

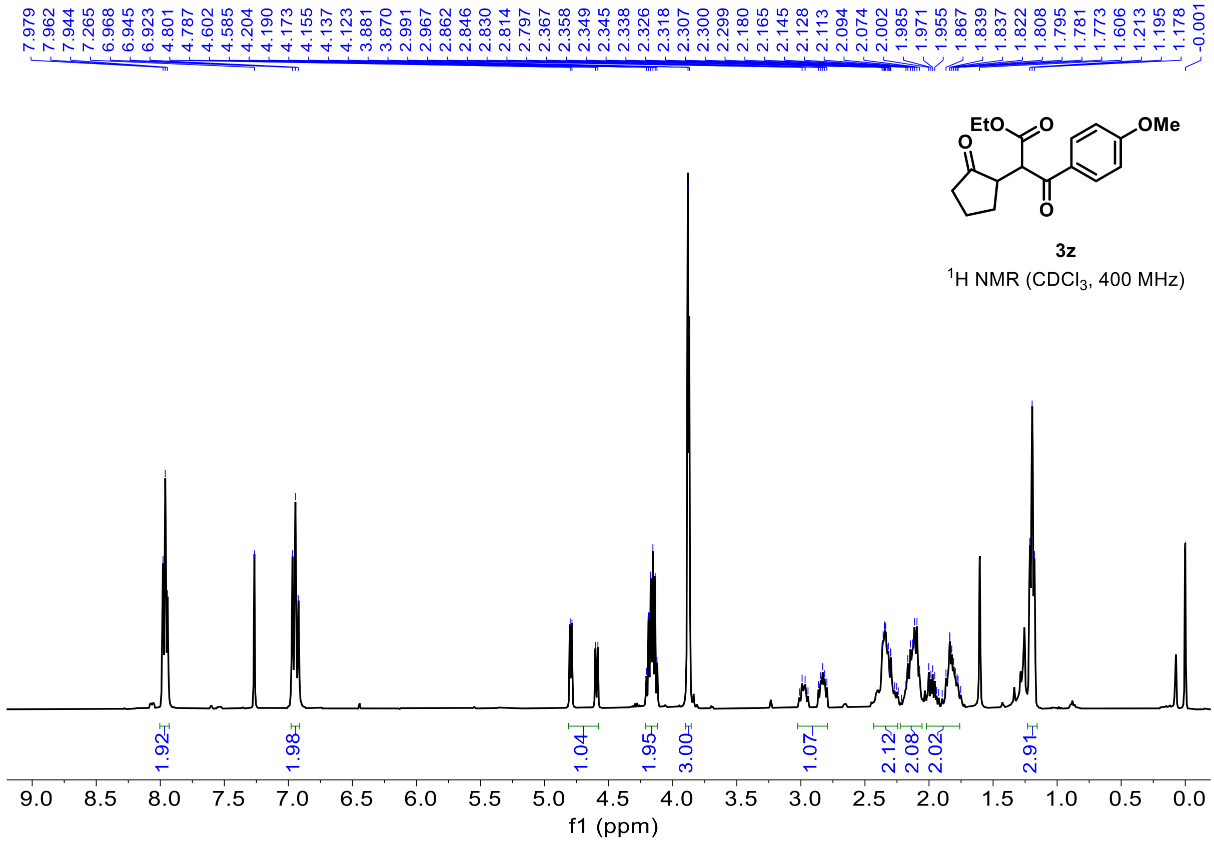

EtO
O
OMe
O
O
3z
^{1}H NMR (CDCl$_3$, 400 MHz)
f1 (ppm)

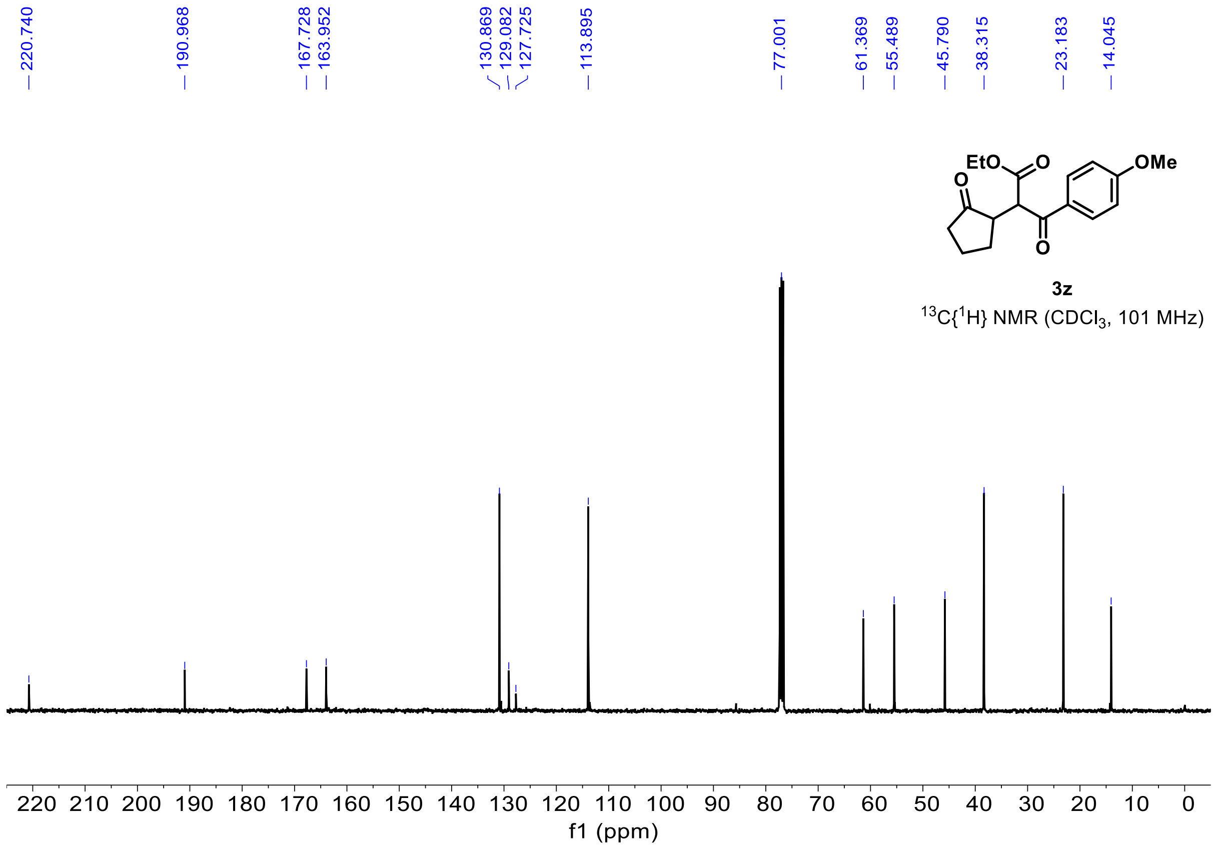

EtO
O
OMe
O
O
3z
^{13}C{^{1}H} NMR (CDCl$_3$, 101 MHz)
f1 (ppm)

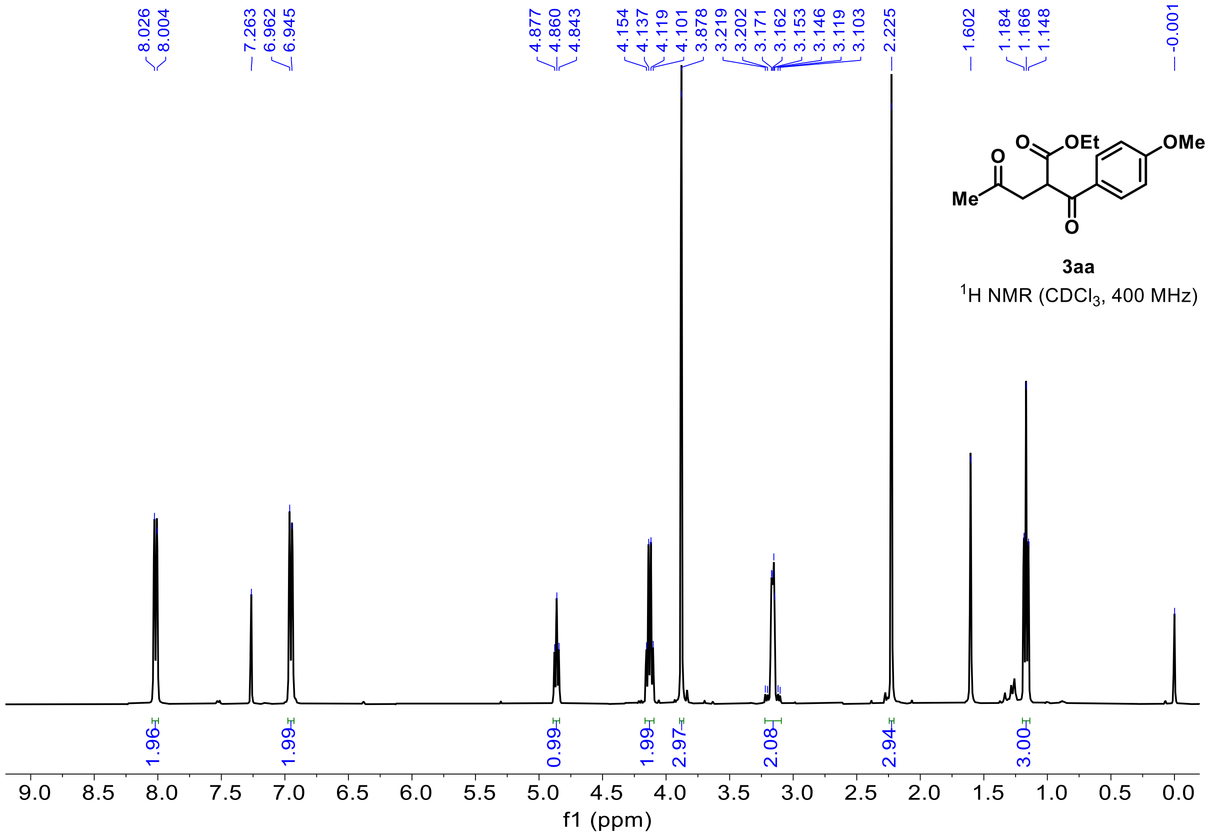
8.026
8.004
7.263
6.962
6.945
4.877
4.860
4.843
4.154
4.137
4.119
4.101
3.878
3.219
3.202
3.171
3.162
3.153
3.146
3.119
3.103
2.225
1.602
1.184
1.166
1.148
-0.001
O
OEt
OMe
O
Me
O
3aa
¹H NMR (CDCl₃, 400 MHz)
1.96
1.99
0.99
1.99
2.97
2.08
2.94
3.00
9.0 8.5 8.0 7.5 7.0 6.5 6.0 5.5 5.0 4.5 4.0 3.5 3.0 2.5 2.0 1.5 1.0 0.5 0.0
f1 (ppm)

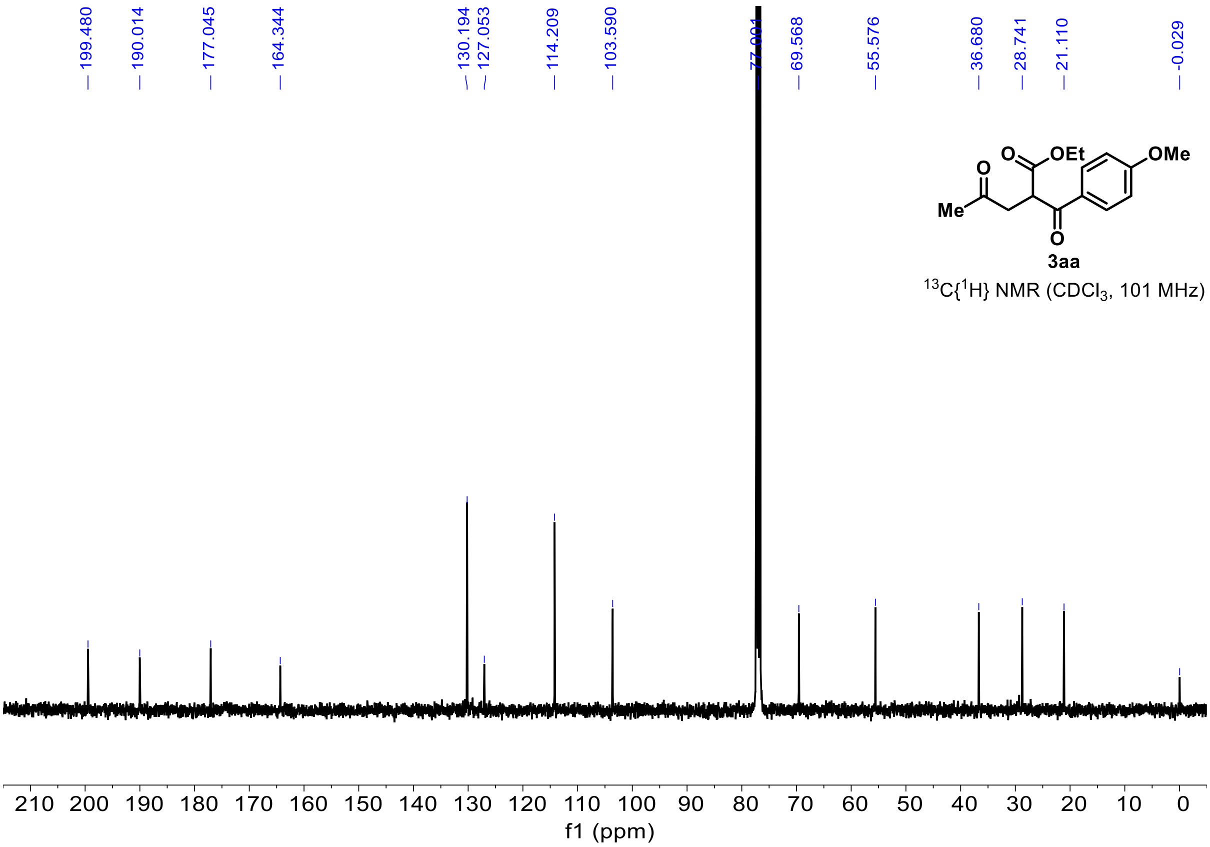
199.480
190.014
177.045
164.344
130.194
127.053
114.209
103.590
77.001
69.568
55.576
36.680
28.741
21.110
-0.029
O
OEt
OMe
O
Me
O
3aa
¹³C{¹H} NMR (CDCl₃, 101 MHz)
210 200 190 180 170 160 150 140 130 120 110 100 90 80 70 60 50 40 30 20 10 0
f1 (ppm)

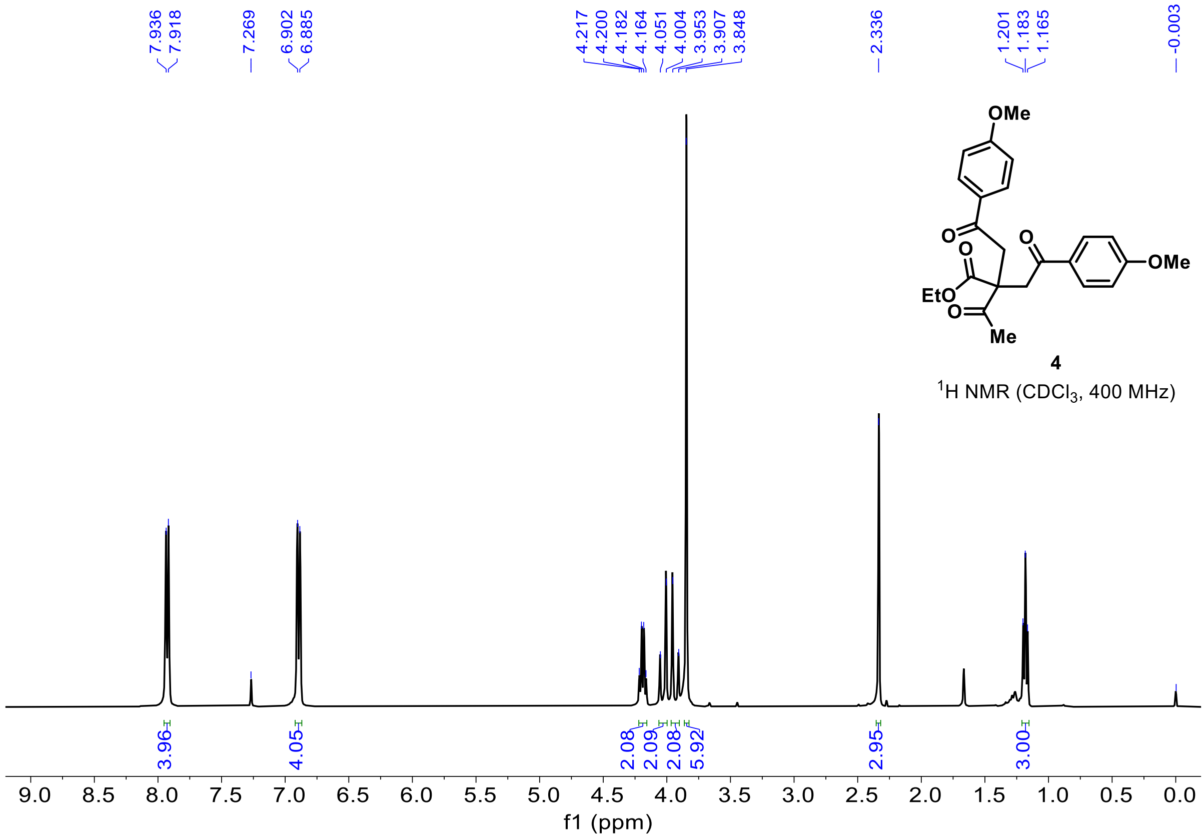
7.936
7.918
7.269
6.902
6.885
4.217
4.200
4.182
4.164
4.051
4.004
3.953
3.907
3.848
2.336
1.201
1.183
1.165
-0.003
OMe
O
O
O
OMe
EtO
O
Me
4
^{1}H NMR (CDCl$_3$, 400 MHz)
3.96
4.05
2.08
2.09
2.08
5.92
2.95
3.00
9.0 8.5 8.0 7.5 7.0 6.5 6.0 5.5 5.0 4.5 4.0 3.5 3.0 2.5 2.0 1.5 1.0 0.5 0.0
f1 (ppm)

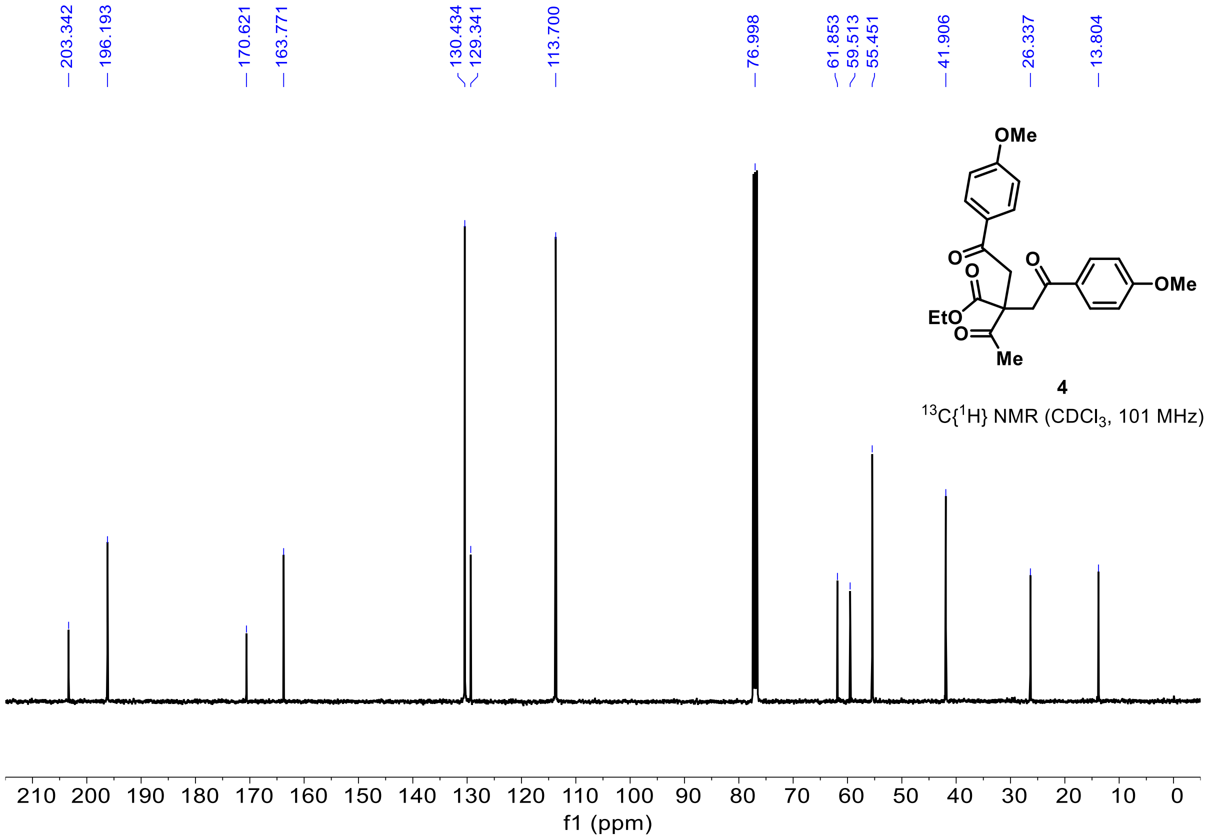
203.342
196.193
170.621
163.771
130.434
129.341
113.700
76.998
61.853
59.513
55.451
41.906
26.337
13.804
OMe
O
O
O
OMe
EtO
O
Me
4
^{13}C{^{1}H} NMR (CDCl$_3$, 101 MHz)
210 200 190 180 170 160 150 140 130 120 110 100 90 80 70 60 50 40 30 20 10 0
f1 (ppm)

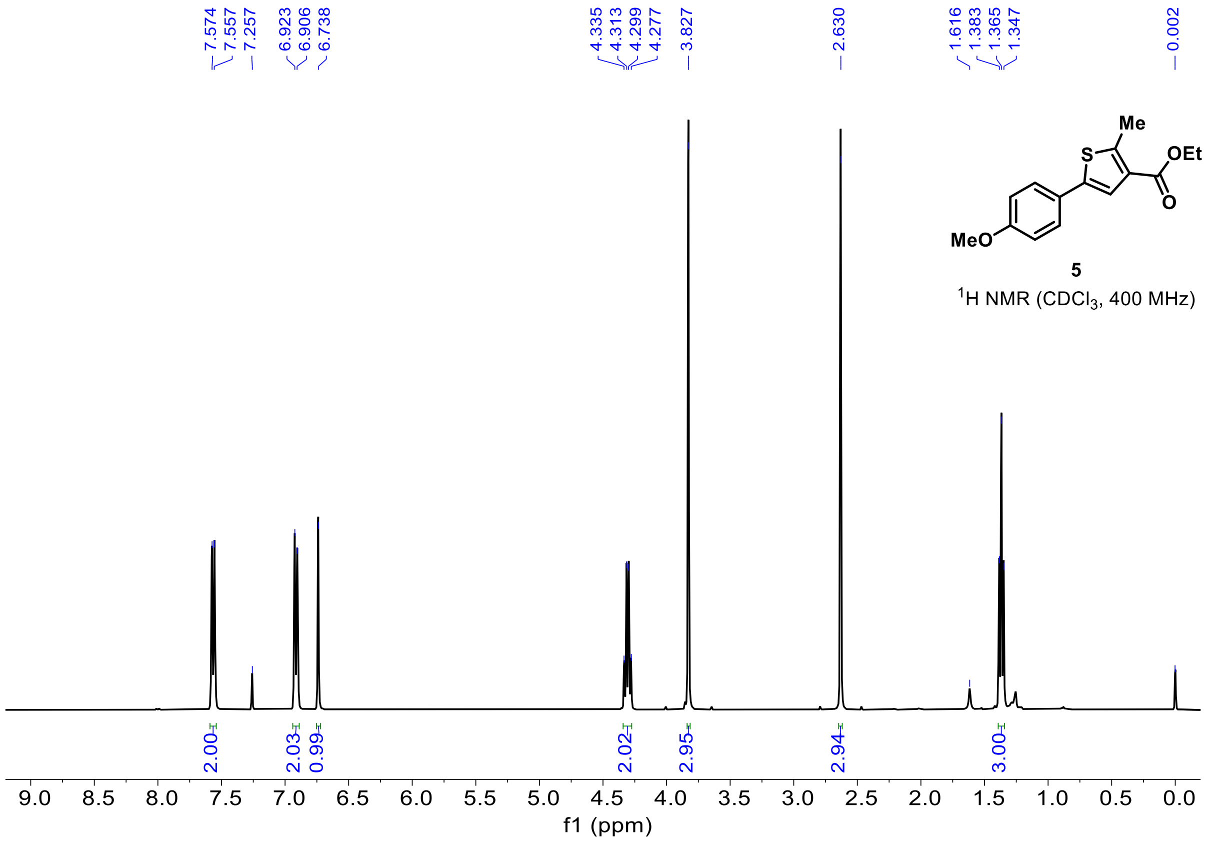

5

^{1}H NMR ($CDCl_3$, 400 MHz)

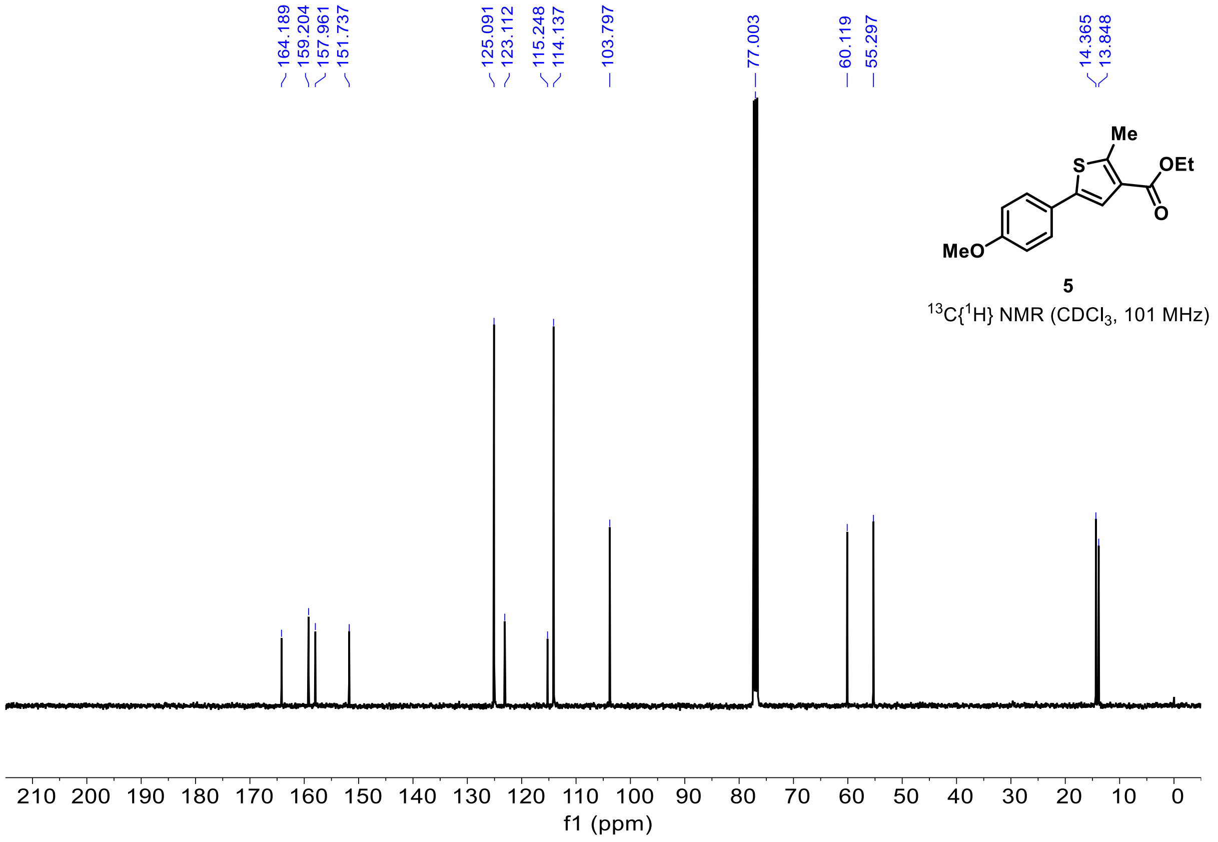

5

$^{13}C\{^{1}H\}$ NMR ($CDCl_3$, 101 MHz)

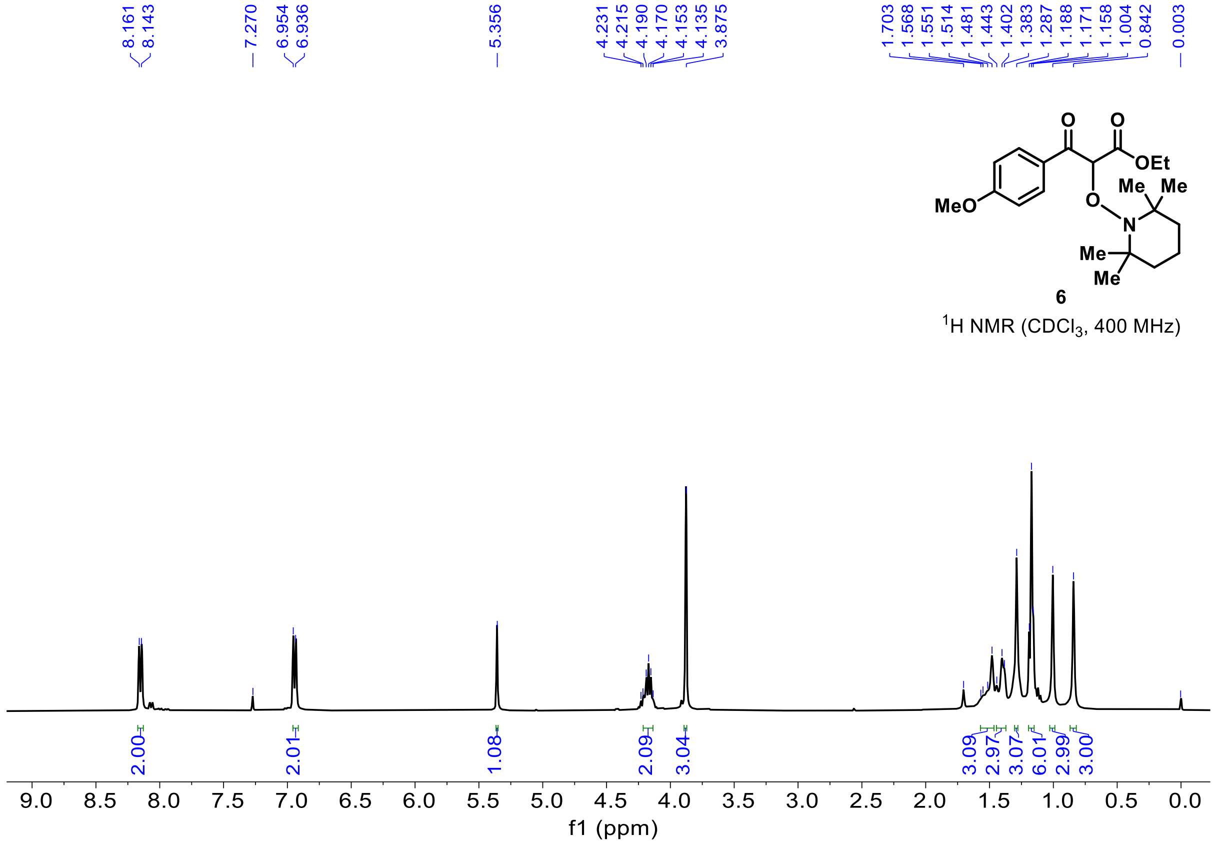
8.161
8.143
7.270
6.954
6.936
5.356
4.231
4.215
4.190
4.170
4.153
4.135
3.875
1.703
1.568
1.551
1.514
1.481
1.443
1.402
1.383
1.287
1.188
1.171
1.158
1.004
0.842
0.003
O
O
OEt
MeO
O
N
Me
Me
Me
Me
6
¹H NMR (CDCl₃, 400 MHz)
2.00
2.01
1.08
2.09
3.04
3.09
2.97
3.07
6.01
2.99
3.00
9.0 8.5 8.0 7.5 7.0 6.5 6.0 5.5 5.0 4.5 4.0 3.5 3.0 2.5 2.0 1.5 1.0 0.5 0.0
f1 (ppm)

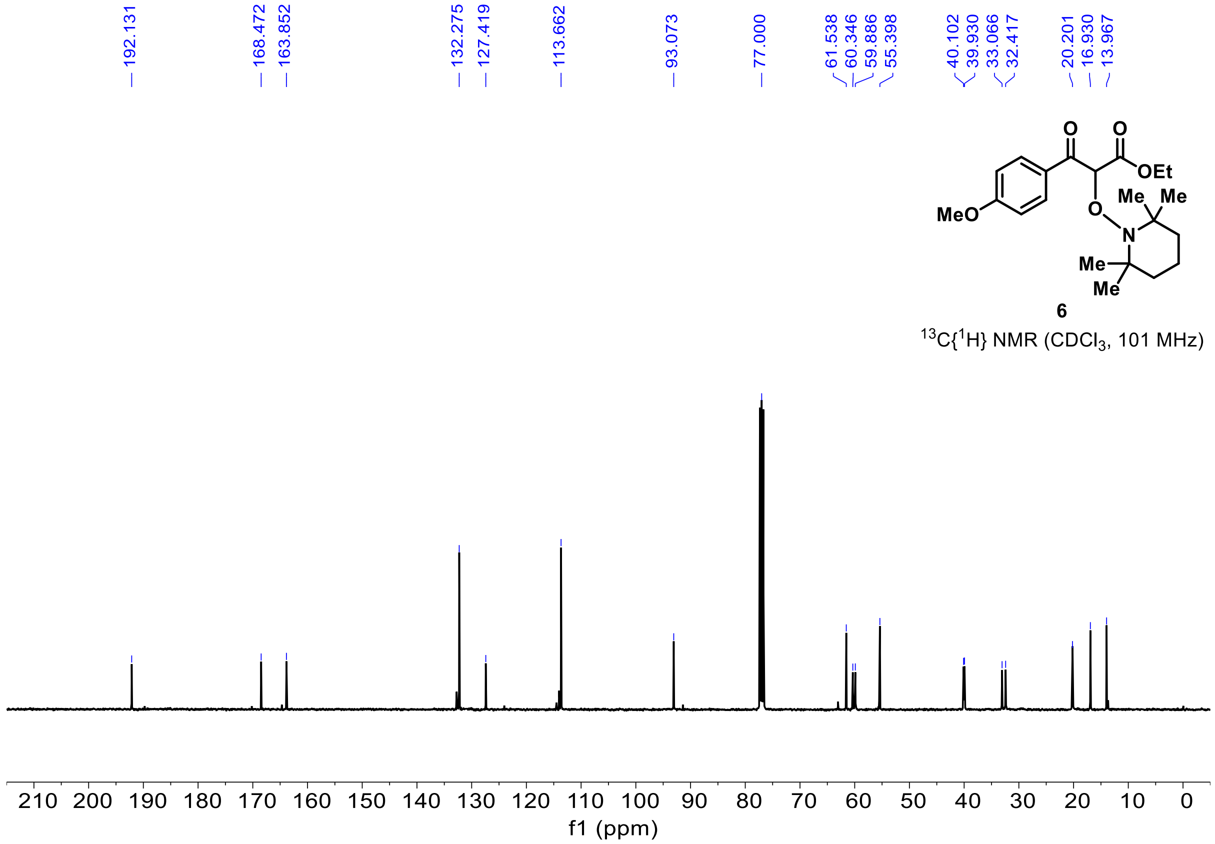
192.131
168.472
163.852
132.275
127.419
113.662
93.073
77.000
61.538
60.346
59.886
55.398
40.102
39.930
33.066
32.417
20.201
16.930
13.967
O
O
OEt
MeO
O
N
Me
Me
Me
Me
6
¹³C{¹H} NMR (CDCl₃, 101 MHz)
210 200 190 180 170 160 150 140 130 120 110 100 90 80 70 60 50 40 30 20 10 0
f1 (ppm)

V. References

(1) Fedorov, O. V.; Kosobokov, M. D.; Levin, V. V.; Struchkova, M. I.; Dilman, A. D. Halogenative Difluorohomologation of Ketones. *J. Org. Chem.* **2015**, *80*, 5870–5876. https://doi.org/10.1021/acs.joc.5b00904

(2) Zhang, B.-S.; Jia, W.-Y.; Gou, X.-Y.; Yang, Y.-H.; Wang, F.; Wang, Y.-M.; Wang, X.-C.; Quan, Z.-J. Synthesis of C8-Aminated Pyrrolo-Phenanthridines or -Indoles via Series C(sp^2 or sp^3)–H Activation and Fluorescence Study. *Org. Lett.* **2022**, *24*, 2104–2109. https://doi.org/10.1021/acs.orglett.2c00318

(3) Wang, X.-M.; Zhang, P.; Xu, Q.; Guo, C.-Q.; Zhang, D.-B.; Lu, C.-J.; Liu, R.-R. Enantioselective Synthesis of Nitrogen–Nitrogen Biaryl Atropisomers via Copper-Catalyzed Friedel–Crafts Alkylation Reaction. *J. Am. Chem. Soc.* **2021**, *143*, 15005–15010. https://doi.org/10.1021/jacs.1c07741

(4) Cunningham, I. D.; McMurry, T. B. H.; Napier, M. P.; Rao, S. N. Photochemistry of Substituted Cyclic Enones. Part 5. 3-Aryl-5-(3-phenylprop-2-enyl)cyclopent-2-enones. *J. Chem. Soc., Perkin Trans. 1* **1986**, 1235–1242. https://doi.org/10.1039/P19860001235

(5) Hu, W.; Zhang, C.; Huang, J.; Guo, Y.; Fu, Z.; Huang, W. Access to Highly Functionalized Indanes from Arynes and α,γ-Diketo Esters. *Org. Lett.* **2019**, *21*, 941–945. https://doi.org/10.1021/acs.orglett.8b03919

(6) Katta, N.; Zhao, Q.-Q.; Mandal, T.; Reiser, O. Divergent and Synergistic Photocatalysis: Hydro- and Oxoalkylation of Vinyl Arenes for the Stereoselective Synthesis of Cyclopentanols via a Formal [4+1]-Annulation of 1,3-Dicarbonyls. *ACS Catal.* **2022**, *12*, 14398–14407. https://doi.org/10.1021/acscatal.2c04736

(7) Chen, M.; Wang, J.; Kan, Y.; Jia, X.; Huang, B.; Li, T.; Zhao, X. Electrocatalytic [3 + 2] Annulation for the Synthesis of Polysubstituted Furans. *Org. Lett.* **2023**, *25*, 4540–4545. https://doi.org/10.1021/acs.orglett.3c01582

(8) Song, B.; Nie, L.; Bozorov, K.; Kuryazov, R.; Zhao, J.; Aisa, H. A. Design, Combinatorial Synthesis and Cytotoxic Activity of 2-Substituted Furo[2,3-*d*]pyrimidinone and Pyrrolo[2,3-*d*]pyrimidinone Library. *Mol. Divers.* **2023**, *27*, 1767–1783. https://doi.org/10.1007/s11030-022-10529-y

(9) Xiong, B.; Shi, C.; Xu, W.; Liu, Y.; Zhu, L.; Tang, K.-W.; Yin, S.-F.; Wong, W.-Y. Visible-Light-Induced Cascade Phosphinoylation/Cyclization of Phenacylmalononitriles with Secondary *H*-phosphine Oxides. *Adv. Synth. Catal.* **2022**, *364*, 4392–4401. https://doi.org/10.1002/adsc.202201055

(10) Wang, L.; Sun, J.; Xia, J.; Li, M.; Zhang, L.; Ma, R.; Zheng, G.; Zhang, Q. Visible Light-Mediated NHCs and Photoredox Co-Catalyzed Radical 1,2-Dicarbonylation of Alkenes for 1,4-Diketones. *Sci. China. Chem.* **2022**, *65*, 1938–1944. https://doi.org/10.1007/s11426-022-1328-5

(11) Zhang, M.-N.; Zhao, M.-N.; Chen, M.; Ren, Z.-H.; Wang, Y.-Y.; Guan, Z.-H. Copper-Catalyzed Radical Coupling of 1,3-Dicarbonyl Compounds with Terminal Alkenes for the Synthesis of Tetracarbonyl Compounds. *Chem. Commun.* **2016**, *52*, 6127–6130. https://doi.org/10.1039/C6CC01942K

(12) Wang, Z.; Qu, Z.; Xiao, F.; Huang, H.; Deng, G.-J. One-Pot Synthesis of 2,3,5-Trisubstituted Thiophenes through Three-Component Assembly of Arylacetaldehydes, Elemental Sulfur, and 1,3-Dicarbonyls. *Adv. Synth. Catal.* **2018**, *360*, 796–800. https://doi.org/10.1002/adsc.201701332
